# Identification of Genetic Variants for Risk Prediction and Early Diagnosis of Age-Related Macular Degeneration in the Taiwanese Population

**DOI:** 10.3390/ijms25063230

**Published:** 2024-03-12

**Authors:** Yu-Chuen Huang, Wen-Ling Liao, Hui-Ju Lin, Yu-Te Huang, Ya-Wen Chang, Ting-Yuan Liu, Yu-Chia Chen, Angel L. Weng, Fuu-Jen Tsai

**Affiliations:** 1 School of Chinese Medicine, China Medical University, Taichung 404328, Taiwan; yuchuen@mail.cmu.edu.tw; 2 Genetic Center, Department of Medical Research, China Medical University Hospital, Taichung 404327, Taiwan; windy87518@gmail.com; 3 Center for Personalized Medicine, China Medical University Hospital, Taichung 404327, Taiwan; wl0129@mail.cmu.edu.tw; 4 Graduate Institute of Integrated Medicine, China Medical University, Taichung 404328, Taiwan; 5 Department of Ophthalmology, China Medical University Hospital, Taichung 404327, Taiwan; tonyhuang791112@gmail.com; 6 Million-Person Precision Medicine Initiative, Department of Medical Research, China Medical University Hospital, Taichung 404327, Taiwan; 092990@tool.caaumed.org.tw (T.-Y.L.); 092989@tool.caaumed.org.tw (Y.-C.C.); 7 American School in Taichung, Taichung 406051, Taiwan; 25angelw@ast.tc.edu.tw; 8 Department of Medical Genetics, China Medical University Hospital, Taichung 404327, Taiwan; 9 Children’s Hospital of China Medical University, Taichung 404327, Taiwan

**Keywords:** age-related macular degeneration, genome-wide association study, polygenic risk score

## Abstract

Age-related macular degeneration (AMD) is the leading cause of blindness in the elderly worldwide. The prevalence and phenotypes of AMD differ among populations, including between people in Taiwan and other regions. We performed a genome-wide association study to identify genetic variants and to develop genetic models to predict the risk of AMD development and progression in the Taiwanese population. In total, 4039 patients with AMD and 16,488 non-AMD controls (aged ≥ 65 years) were included. We identified 31 AMD-associated variants (*p* < 5 × 10^−8^) on chromosome 10q26, surrounding *PLEKHA1-ARMS2-HTRA1*. Two genetic models were constructed using the clump and threshold method. Model 1 included the single nucleotide polymorphism rs11200630 and showed a 1.31-fold increase in the risk of AMD per risk allele (95% confidence interval (CI) = 1.20–1.43, *p* < 0.001). In model 2, 1412 single-nucleotide polymorphisms were selected to construct a polygenic risk score (PRS). Individuals with the top 5% PRS had a 1.40-fold higher AMD risk compared with that of individuals with a PRS in the bottom quartile (95% CI = 1.04–1.89, *p* = 0.025). Moreover, the PRS in the upper quartile was related to a decreased age at AMD diagnosis by 0.62 years (95% CI = −1.15, −0.09, *p* = 0.023). Both genetic models provide useful predictive power for populations at high risk of AMD, affording a basis for identifying patients requiring close follow-up and early intervention.

## 1. Introduction

Age-related macular degeneration (AMD) is the leading cause of visual impairment and blindness in the older adult population worldwide [1]. AMD is a prevalent degenerative disease affecting the retina and is influenced by a complex interplay of genetic, environmental, demographic, and geographical factors [2,3]. Late-stage AMD is classified into two clinical forms: dry or non-exudative and wet or exudative (neovascular AMD). An initial sign of AMD is the build-up of protein–lipid deposits known as drusen on the Bruch’s membrane beneath the retinal pigment epithelium (RPE). This accumulation disrupts RPE function and leads to the loss of photoreceptors in the macula [4]. 

Aging remains the most significant risk factor for AMD. Additional evidence suggests that sex, racial, and ethnic differences affect the prevalence of AMD [5,6,7,8]. A recent meta-analysis of populations in mainland China, Taiwan, Singapore, and the United States showed that the prevalence of early and late AMD in the Chinese population is lower than that in Caucasian populations [5]. However, the prevalence of exudative AMD in the Taiwanese population differs from those in other ethnicities and is higher than that in the Chinese population in Beijing [9]. Moreover, a higher body mass index and smoking history are modifiable risk factors for AMD development and progression [10,11,12].

AMD is considered as a highly heritable disease; results from a US twin study showed that genetic factors play a substantial role in the etiology of AMD and associated macular characteristics, with genetic variations making a 46–71% contribution to the overall severity of AMD [13]. In addition, the greater concordance rate for AMD in monozygotic twin pairs than in dizygotic twin pairs suggests a genetic predisposition to AMD [14,15]. A recent comprehensive genome-wide association study (GWAS) of subjects of predominantly European ancestry identified genetic variants across 34 loci, accounting for over 50% of AMD heritability [16]. Although AMD exhibits a complex genetic landscape involving various common and rare variants, *ARMS2/HTRA1* and *CFH* significantly contribute to the overall risk. A highly predictive model for people from European countries consisting of 26 AMD-associated single nucleotide polymorphisms (SNPs) as well as age and sex demonstrated good performance in predicting late-stage AMD with an area under the curve (AUC) of 0.82 (95% confidence interval (CI): 0.79–0.86), surpassing the performance of non-genetic risk models (AUC: 0.78; 95% CI: 0.74–0.82) [17]. In another study, additional AMD risk variants were identified in a GWAS involving 16,144 patients with AMD, leading to the development of an AMD polygenic risk score (PRS) based on 52 AMD-associated SNPs, including seven rare variants with a minor allele frequency below 1% [16]. A recently constructed AMD PRS based on 63 risk loci showed potential for predicting risk in East Asian, South Asian, and Latin populations [18]; the AUC for the East Asian population (116 patients with AMD and 4006 control participants) was 0.60 (95% CI: 0.54–0.65). In a study of the largest East Asian sample to date (6345 exudative AMD cases and 15,980 controls), some genetic loci conferring AMD susceptibility detected in East Asians were shared with Europeans; however, AMD in East Asians may also have a distinct genetic signature [19]. 

Asian populations appear to have a distinct clinical presentation of exudative AMD and different responses to vascular endothelial growth factor treatment [19,20]. Particularly, the prevalence and phenotypes of AMD differ between European populations and the Taiwanese population. Therefore, we conducted a large-scale study of the genetic factors associated with AMD and developed genetic models to predict the AMD risk in a Taiwanese population, integrating other demographic data. We aimed to narrow the gap in underrepresented populations relative to European individuals and provide accurate prediction models of AMD risk. 

## 2. Results

### 2.1. Characteristics of the Study Population

**Figure 1:**
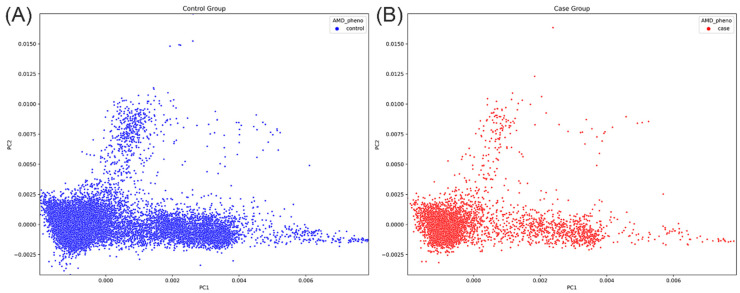
Principal component analyses of genetic data between (**A**) 16,448 non-AMD controls and (**B**) 4039 AMD cases in the present study.

A total of 20,489 individuals, including 4039 patients with AMD and 16,448 non-AMD controls (age ≥ 65 years), were included in the study. For GWAS discovery, individuals were randomly divided at a ratio of 7:3 into a cohort of 14,341 individuals (2827 with AMD and 11,514 non-AMD controls) and another cohort of 6146 individuals (1212 and 4938 individuals with AMD and non-AMD controls) for replication. Among these patients, 1980 and 865 patients were identified as having dry-type AMD in the discovery and replication groups, and 847 and 347 patients were identified as having wet-type AMD in the discovery and replication groups, respectively. The mean ages at AMD diagnosis were 69.8 ± 9.7 and 69.6 ± 10.0 years in the discovery and replication groups, respectively. In the discovery group, the sex ratios (female/male) were 1.05 and 1.25 in AMD cases and non-AMD controls, respectively. In the replication group, the sex ratios (female/male) were 1.01 and 1.26 in AMD cases and controls, respectively. The proportions of males among patients with AMD were significantly higher than those in control participants; 48.7% vs. 44.5% (*p* < 0.001) and 49.8% vs. 44.2% (*p* < 0.05) in the discovery and replication groups, respectively. A body mass index (BMI) > 25 kg/m^2^ and smoking status rates did not differ significantly between patients with AMD and non-AMD controls in either cohort. Characteristics of the study population are summarized in Table 1. In addition, principal component analysis revealed a similar genetic variation structure between AMD cases and non-AMD controls, as shown in Figure 1.

### 2.2. GWAS for Identifying AMD-Associated SNPs

**Figure 2:**
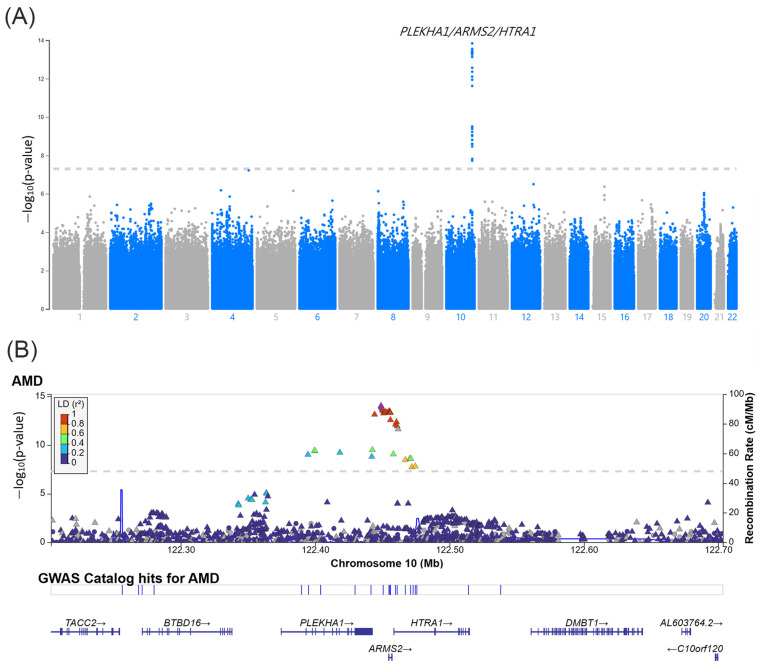
(**A**) Manhattan plot of the GWAS in the discovery cohort which included 2824 AMD patients and 11,514 non-AMD controls. The bottom gray dashed line indicates the genome-wide significance threshold (*p* < 5.0 × 10^−8^). (**B**) Regional information of the AMD-associated SNPs at the *PLEKHA1/ARMS2/HTRA1* locus.

To identify AMD-associated SNPs, we performed a GWAS of the discovery cohort. As shown in the Manhattan plot in Figure 2A, the GWAS revealed 31 AMD-associated SNPs reaching genome-wide significance (*p* < 5 × 10^−8^) on chromosome 10q26. Detailed information on the 31 SNPs is shown in Supplementary Table S1. These SNPs are shown surrounding the *PLEKHA1*, *LOC105378525*, *ARMS2*, and *HTRA1* (Figure 2B).

### 2.3. Linkage Disequilibrium and Haplotype Block Analyses

**Figure 3:**
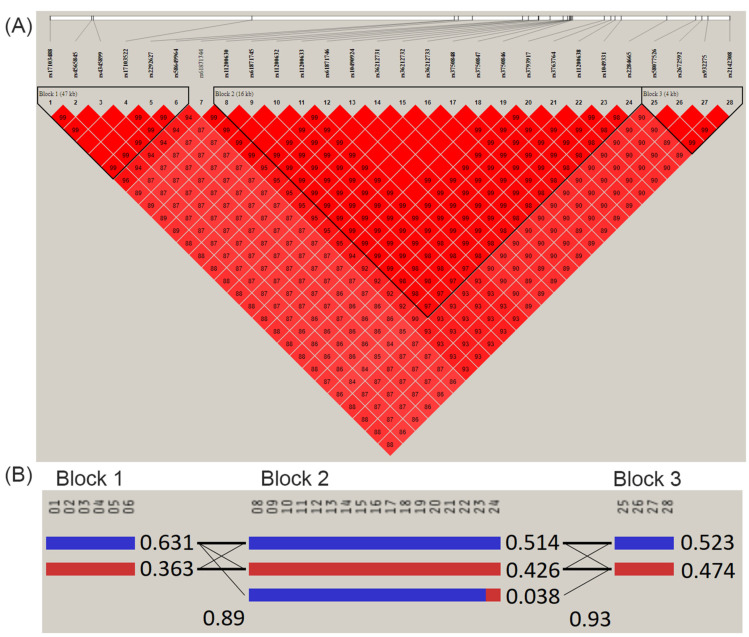
(**A**) Linkage disequilibrium (LD) plot of the AMD-associated SNPs on chromosome 10 around 122.40 to 122.50 Mb, consisting of three haplotype block patterns. The LD pattern was derived from the all-study population and the color intensity decreases from red to white according to D’ value. (**B**) Patterns of haplotype blocks and haplotype frequencies. The order of SNPs is as follows: Block 1 (47 Kb), 01: rs17103488; 02: rs4565845; 03: rs4345899; 04: rs17103522; 05: rs2292627; 06: rs58649964. Block 2 (16 Kb), 08: rs11200630; 09: rs61871745; 10: rs11200632; 11: rs11200633; 12: rs61871746; 13: rs10490924; 14: rs36212731; 15: rs36212732; 16: rs36212733; 17: rs3750848; 18: rs3750847; 19: rs3750846; 20: rs3793917; 21: rs3763764; 22: rs11200638; 23: rs1049331; 24: rs2284665. Block 3 (4 Kb), 25: rs58077526; 26: rs2672592; 27: rs932275; 28: rs2142308.

Pairwise linkage disequilibrium (LD) analysis revealed AMD-associated SNPs in 10q26 with high LD (D’ > 0.977) and consisted of three haplotype block patterns on chromosome 10 around 122.40–122.50 Mb (Figure 3). The frequencies of haplotype CCTCGA in block 1, haplotype CAGTCTTGCGTCGGATT in block 2, and haplotype CTAC in block 3 were significantly higher in AMD cases than in non-AMD controls, as shown in Table 2.

### 2.4. PRS for AMD Construction

A PRS was generated using GWAS summary statistics from our discovery cohort and implemented using PRSice-2. The best-fit PRS for AMD, including one SNP (rs11200630), was selected at a *p*-value threshold of 5 × 10^−8^, at which the model fit exhibited the highest R^2^ score (0.009, model 1). The second-best-fit PRS for AMD with the second-highest R^2^ score (0.001, model 2), based on 1412 SNPs, was selected at a *p*-value threshold of 3.5 × 10^−4^. The 1412 SNPs in model 2 are listed in Supplementary Table S2. 

### 2.5. Genetic Factors and PRS Predict the Risk of AMD Development

Two prediction models were evaluated in the replication cohort, which included 1212 AMD cases and 4934 non-AMD controls. According to model 1, the increase per risk allele C in rs11200630 was 1.31-fold (95% CI = 1.20–1.43, *p* < 0.001), adjusted for age at diagnosis and sex. Furthermore, the increases per risk allele C in rs11200630 in the adjusted odds ratio (OR) were 1.22-fold (95% CI = 1.10–1.35, *p* < 0.001) and 1.55-fold (95% CI = 1.33–1.81, *p* < 0.001) for dry and wet AMD, respectively (Table 3). In terms of the AMD PRS constructed from 1412 SNPs (model 2), individuals with the top 5% of PRS had adjusted ORs that were 1.40-fold (95% CI = 1.04–1.89, *p* = 0.025) and 1.46-fold (95% CI = 1.04–2.04, *p* = 0.028) higher than those of individuals in the bottom quartile with respect to the PRS in AMD and dry AMD, respectively. However, the PRS did not significantly differ in individuals in the top 5% compared with individuals in the bottom quartile in wet AMD (OR = 1.28, 95% CI = 0.76–2.13, *p* = 0.353), as shown in Table 3. 

### 2.6. Performance of AMD Prediction Models

We evaluated the performance of the two prediction models using the replication cohort. The AUCs for rs11200630 (model 1) and PRS based on 1412 SNPs (model 2), including the covariates age, sex, BMI, and smoking status, were 0.662 (95% CI = 0.641–0.683, *p* < 0.001) and 0.648 (95% CI = 0.627–0.670, *p* < 0.001) for overall AMD and 0.654 (95% CI = 0.629–0.679, *p* < 0.001) and 0.646 (95% CI = 0.621–0.671, *p* < 0.001) for dry AMD; these values were 0.692 (95% CI = 0.656–0.728, *p* < 0.001) and 0.659 (95% CI = 0.620–0.698, *p* < 0.001) for wet AMD (Table 4).

### 2.7. Association between PRS and Age at AMD Diagnosis

In the multivariable regression model, for individuals with AMD, the PRS in the upper quartile was associated with a significantly decreased age at diagnosis by 0.62 years (beta: −0.62 (95% CI = −1.15, −0.09); *p* = 0.023). Smoking significantly decreased the age at diagnosis of AMD by 6.95 years (beta: −6.95 (95% CI = −1.15, −0.39); *p* = 0.039). However, age at the time of AMD diagnosis was not significantly affected by sex. Age at AMD diagnosis was significantly negatively correlated with the PRS quartile and smoking status, as shown in Table 5.

## 3. Discussion

We performed a GWAS of AMD in a Taiwanese population including 4039 cases and 16,448 controls to identify genetic loci related to AMD. We generated two prediction models: model 1 based on rs11200630 and model 2 based on 1412 SNPs. A cluster of SNPs with high LD surrounding the *PLEKHA1*, *LOC105378525*, *ARMS2*, and *HTRA1* loci on chromosome 10q26 was identified in the GWAS. The rs11200630 was the most significant SNP in LD after rs59616332 and showed high LD with rs10490924 (*r*^2^ = 0.941), which is associated with an elevated risk of AMD in other Taiwanese populations [21]. The rs11200630 is a non-coding SNP and was related to an elevated AMD risk in the present study; this SNP was located near *ARMS2* and *HTRA1*, which are strong genetic risk factors for AMD according to a GWAS of European and East Asian populations [16,18,19,22,23]. A previous study of human-derived RPE tissues demonstrated that HTRA1 levels were reduced by chromosome 10q26 risk-associated variants (diplotypes of rs11200630/rs10490924) and that HTRA1 levels are reduced within the RPE–Bruch’s membrane interface during aging [24]. Genetic variants within the 10q26 and 1q32 regions contain *CFH* and *CFHR1–5*, which together account for more than 50% of the AMD risk in European–American populations [24,25,26]. CFH is also involved in chronic inflammatory responses and drusen formation [25]. Among the most well-studied and replicated *CFH* variants associated with AMD risk is rs1061170, which is a substitution at amino acid 402 (Y402H); the frequency of the C allele is much lower in Chinese patients than in Caucasian patients with or without AMD [27,28]. In our cohort, we did not identify any SNPs in *CFH* that reached GWAS significance (*p* < 5 × 10^−8^), despite the reported association between this locus and AMD in East Asian populations [19]. Consistent with these findings, variants in *CFH* did not reach GWAS significance for AMD in another Taiwanese population [21]. This gene may play a less important role in AMD development in the Taiwanese population than in other populations.

After removing SNPs that were in LD, the best-fit model only included rs11200630 (model 1). However, the second-best-fit model was based on 1412 SNPs, including rs11200630 (model 2). When including age at diagnosis, sex, BMI, and smoking status, the predictive performance was similar between models 1 and 2. The AUC for AMD prediction in model 1 (AUC_model1_: 0.662) was slightly higher than that in model 2 (AUC_model2_: 0.659). This result suggests that one SNP, rs11200630, had a similar AMD prediction performance as the PRS comprising 1412 SNPs in the Taiwanese population. Both models were suitable for predicting dry AMD (AUC: 0.654 for model 1 and 0.646 for model 2) and wet AMD (AUC: 0.692 for model 1 and 0.659 for model 2). Previously reported AUCs for genetic models for AMD prediction in Taiwan were 0.651–0.693 [21], which are similar to those in our study. A recently constructed AMD PRS for individuals of European ancestry showed an AUC of 0.60 (95% CI: 0.54–0.65) for East Asians [18]. A predictive model constructed in the Three Continent AMD Consortium study of populations of European ancestry consisting of 26 AMD-associated SNPs as well as age and sex demonstrated superior performance in predicting late-stage AMD with an AUC of 0.82 (95% CI: 0.79–0.86) [17]. Notably, the achieved AUC values were relatively low for Asians, even when the prediction model was constructed for the Taiwanese population. This observation suggests that the disease is influenced by a complex interplay of genetic, environmental, demographic, and geographical factors, in contrast to Western contexts where AMD is a highly heritable disease. Higher AUC values may be obtained in further studies and from replication sets with larger sample sizes. However, genetic prediction methods can still be used to identify high-risk groups that require close follow-up and early intervention. We found that patients with AMD with a higher PRS in model 2 may have an earlier diagnosis of AMD. Considering that AMD can remain asymptomatic for a long duration or exhibit subtle symptoms that may not attract the patient’s attention, thorough risk assessment is crucial. Such assessment can aid in determining the appropriate frequency of ophthalmic follow-up visits and identifying individuals who would benefit greatly from self-monitoring [29]. Model 1 showed slightly higher predictive performance for the AMD risk and a relatively simple genetic profile compared with those of model 2.

This study had several strengths. First, this is the largest AMD GWAS performed on a Han Chinese population in Taiwan, providing insight into differences in the genetic basis of AMD between Asian and European populations. Second, we included information on smoking status and BMI as risk factors for AMD in our predictive model to increase the prediction accuracy. One limitation of this study is that only a small proportion of the heritability can be explained by the observed genetic variants in GWAS. Using the PRS is not superior to a single SNP for predicting AMD risk. Additionally, our sample size was small and we used a cross-sectional design. Further prospective longitudinal studies incorporating more comprehensive variables are necessary to establish a more accurate PRS model. 

## 4. Materials and Methods

### 4.1. Data Source

Genetic information and electronic medical records of the study population were obtained from the Precision Medicine Project, which was established at the China Medical University Hospital (CMUH) and has enrolled more than 300,000 participants. An informed consent form was signed by each participant before the collection of blood samples for genome-wide genotyping and clinical information from their electronic medical records at the CMUH. Detailed information regarding the project has been reported previously [30]. The recruitment and sample collection procedures were approved by the ethics committees of the CMUH (CMUH109-REC2-185 and CMUH111-REC1-176).

### 4.2. Study Population

The study population included subjects of Taiwanese Han-Chinese ancestry [31]. Patients with AMD were identified according to the International Classification of Diseases Ninth Revision (ICD-9) codes 362.50, 362.51, and 362.57 and the International Classification of Diseases Tenth Revision (ICD-10) codes H35.30, H35.31, H35.36 (nonexudative or dry AMD), ICD-9 code 362.52, and ICD-10 code H35.32 (exudative or wet AMD). The non-AMD control cohort included patients who visited ophthalmologists and were aged ≥ 65 years without any AMD diagnosis codes. Individual-level data for AMD risk factors, including sex, age at diagnosis, BMI, and smoking status, were also collected from the Precision Medicine Project at the CMUH.

### 4.3. Genotyping and Discovery GWAS 

Genotyping was performed using the Axiom Taiwan Precision Medicine version 1 customized SNP array (Thermo Fisher Scientific, Waltham, MA, USA), which was produced to obtain the maximum amount of genetic information from the samples of the Taiwanese population. This array comprises approximately 740 K SNPs across the whole human genome. Information on the genotyping, quality control, and imputation methods for all study participants has been described previously [32,33,34,35]. 

For the discovery GWAS, patients with and without AMD were randomly divided at a ratio of 7:3 into a cohort of 14,343 participants (2827 individuals with AMD and 11,514 control individuals without AMD) and another cohort of 6146 participants (1212 and 4934 individuals with AMD and without AMD, respectively); patients in the second group were used for further replication.

### 4.4. PRS Construction 

For PRS model construction, we used summary statistics from the discovery AMD GWAS using the clumping and thresholding (C+T) method in PRSice-2 version 2.3.5 [36]. After LD clumping, one SNP and 1412 SNPs at *p*-value thresholds of 5 × 10^−8^ and 3.5 × 10^−4^, respectively, and the corresponding estimated β-coefficient for their effect alleles as weights were included in the PRS calculation using PLINK version 2.0 [37]. Two candidate PRS models were created for further replication.

### 4.5. Statistical Analysis

Student’s *t*-tests for continuous variables and chi-square tests for categorical variables were used to compare characteristics and clinical data between the AMD and non-AMD groups. ORs and 95% CIs were determined using logistic regression analyses. Receiver operating characteristic curves were generated to quantify the predictive accuracy of the models and the AUC was used to assess the discriminatory ability. A general linear model was used to analyze the association between the PRS and age at AMD diagnosis. Genetic differences between the AMD and non-AMD groups were analyzed using principal component analysis performed in Python with the Pandas, NumPy, Seaborn, and Matplotlib packages. Haploview software (version 4.2) was used to estimate the frequencies of haplotypes and to calculate the LD between any two loci [38]. Other statistical analyses were performed using SPSS version 22 (IBM Co., Armonk, NY, USA) and R Statistical Software (version 3.6.1, R Core Team, 2019-07-05). Statistical significance was set at *p* < 0.05. 

## 5. Conclusions

Both genetic models show predictive power for populations at high risk of AMD, providing a basis for identifying patients requiring close follow-up and early intervention. Compared with model 2, model 1 showed a slightly higher predictive performance for the AMD risk and a relatively simple genetic profile.

## Figures and Tables

**Table 1:** Characteristics of the participants in the discovery and replication cohorts.

	Discovery Cohort (n = 14,343)		Replication Cohort (n = 6146)	
	Control Participants	AMD Patients	Control Participants	AMD Patients
Sample size		2827		1212
Dry type (%) Wet type (%)	11,514	1980 (70.0) 847 (30.0)	4934	865 (71.4) 347 (28.6)
Age (yrs, mean ± SD)	74.3 ± 7.3	74.7 ± 10.1 69.8 ± 9.7 ^1^	74.3 ± 7.3	74.9 ± 10.4 69.6 ± 10.0 ^1^
Sex				
Male (%) Female (%)	5121 (44.5) 6393 (55.5)	1376 (48.7) ** 1451 (51.3)	2183 (44.2) 2751 (55.8)	603 (49.8) * 609 (50.2)
BMI (kg/m^2^) ^2^				
≤25 (%) >25 (%)	5719 (55.6) 4573 (44.4)	1446 (56.3) 1124 (43.7)	2390 (53.8) 2054 (46.2)	617 (56.6) 473 (43.3)
Smoking status ^2^				
Smoking (%) Non-smoking (%)	894 (9.2) 8815 (90.8)	246 (10.1) 2193 (89.9)	392 (9.3) 3813 (90.7)	102 (9.9) 928 (90.1)

^1^ Age of diagnosis; ^2^ data are not available for all of the study population; * *p* < 0.05, ** *p* < 0.001 compared with control participants.

**Table 2:** The frequencies of the major haplotypes for each haplotype block.

Haplotype Block Pattern	Haplotype Frequency		*p* Value
	AMD Cases	Non-AMD Controls	
**Block 1 ^1^**			
TACTTT	0.590	0.641	2.26 × 10^−16^
CCTCGA	0.404	0.353	2.23 × 10^−16^
**Block 2 ^2^**			
TGACTGGATTCTCAGCG	0.473	0.524	1.42 × 10^−16^
CAGTCTTGCGTCGGATT	0.475	0.415	4.60 × 10^−22^
TGACTGGATTCTCAGCT	0.033	0.039	0.009
**Block 3 ^3^**			
AGGG	0.482	0.533	3.87 × 10^−15^
CTAC	0.515	0.463	1.75 × 10^−15^

^1^ Block 1 (47 Kb): rs17103488/rs4565845/rs4345899/rs17103522/rs2292627/rs58649964. ^2^ Block 2 (16 Kb): rs11200630/rs61871745/rs11200632/rs11200633/rs61871746/rs10490924/rs36212731/rs36212732/rs36212733/rs3750848/rs3750847/rs3750846/rs3793917/rs3763764/rs11200638/rs1049331/rs2284665. ^3^ Block 3 (4 Kb): rs58077526/rs2672592/rs932275/rs2142308.

**Table 3:** Association between genetic factors and the risk of AMD.

	Adjusted Odds Ratio (95% CI), *p* Value ^1^		
	Overall AMD	Dry AMD	Wet AMD
Model 1 rs11200630 (per risk allele C)	1.31 (1.20–1.43) *p* < 0.001	1.22 (1.10–1.35) *p* < 0.001	1.55 (1.33–1.81) *p* < 0.001
Model 2 PRS_1412 SNP (The top 5% vs. the bottom quartile)	1.40 (1.04–1.89) *p* = 0.025	1.46 (1.04–2.04) *p* = 0.028	1.28 (0.76–2.13) *p* = 0.353

^1^ Adjusted for age at diagnosis and sex.

**Table 4:** Performance in two AMD prediction models in the replication cohort.

	AUC (95% CI), *p*-Value		
	Overall AMD	Dry AMD	Wet AMD
Covariates ^1^	0.646 (0.625–0.668), *p* <0.001)	0.646 (0.621–0.670), *p* < 0.001	0.656 (0.617–0.695), *p* < 0.001
Model 1 (rs11200630)			
SNP only	0.549 (0.531–0.568), *p* <0.001)	0.537 (0.516–0.558), *p* = 0.001	0.580 (0.547–0.613), *p* < 0.001
SNP + covariates	0.662 (0.641–0.683), *p* <0.001)	0.654 (0.629–0.679), *p* < 0.001	0.692 (0.656–0.728), *p* < 0.001
Model 2 (PRS_1412 SNP)			
PRS only	0.511 (0.493–0.529), *p* = 0.225)	0.507 (0.486–0.528), *p* = 0.499	0.521 (0.490–0.552), *p* = 0.183
PRS + covariates	0.648 (0.627–0.670), *p* < 0.001	0.648 (0.627–0.670), *p* < 0.001	0.659 (0.620–0.698), *p* < 0.001

^1^ Covariates including age, sex, BMI, and smoking status.

**Table 5:** Effects of polygenic risk scores and other risk factors on the age of AMD diagnosis.

Multivariable Analysis	Beta (95% CI) ^1^	*p* Value ^1^
Variable		
PRS quartile	−0.62 (−1.15, −0.09)	0.023
Smoking status Smoking vs. non-smoking	−6.95 (−13.6, −0.34)	0.039
Sex Male vs. female	0.24 (−1.01, 1.48)	0.706

^1^ Adjusted for other covariates.

## Data Availability

The data presented in this study are available on request from the corresponding author. The data are not publicly available due to participant privacy.

## Supplementary Materials

The following supporting information can be downloaded at: https://www.mdpi.com/article/10.3390/ijms25063230/s1.
