# Supplementary material for: Identification of Genetic Variants for Risk Prediction and Early Diagnosis of Age-Related Macular Degeneration in the Taiwanese Population
Source: Int J Mol Sci. 2024 Mar 12;25(6):3230. doi: 10.3390/ijms25063230 (PMC10970312; doi:10.3390/ijms25063230)
Supplement: Supplementary file 1 [file ijms-25-03230-s001.zip › sTable 1_R2.pdf]

Supplementary Table 1. List of 31 AMD-associated SNPs reaching the genome-wide significance level (p < 5.0E-08)

Chromosome	Position	SNP ID	Gene		MAF	REF	ALT	A1	OR	LOG(OR)_SE	L95	U95	P
10	122394653	rs17103488	PLEKHA1		0.3614	T	C	C	1.2180	0.0322	1.1434	1.2975	9.61E-10
10	122399534	rs4565845	PLEKHA1		0.3648	A	C	C	1.2226	0.0322	1.1480	1.3022	4.10E-10
10	122399727	rs4345899	PLEKHA1		0.3647	C	T	T	1.2235	0.0322	1.1487	1.3031	3.64E-10
10	122418363	rs17103522	PLEKHA1		0.3660	T	C	C	1.2197	0.0321	1.1454	1.2988	5.86E-10
10	122442149	rs2292627	PLEKHA1		0.3687	T	G	G	1.2130	0.0320	1.1393	1.2914	1.54E-09
10	122442610	rs58649964	PLEKHA1		0.3704	T	A	A	1.2224	0.0319	1.1483	1.3012	3.11E-10
10	122444271	rs61871744	None		0.4460	T	C	C	1.2588	0.0308	1.1852	1.3371	7.46E-14
10	122449046	rs59616332	LOC105378525		0.4273	GATAAAC	G	G	1.2639	0.0305	1.1907	1.3417	1.46E-14
10	122450168	rs11200630	LOC105378525		0.4312	T	C	C	1.2573	0.0301	1.1853	1.3338	2.81E-14
10	122450853	rs61871745	LOC105378525		0.4307	G	A	A	1.2534	0.0300	1.1818	1.3295	5.56E-14
10	122452020	rs11200632	LOC105378525		0.4293	A	G	G	1.2562	0.0301	1.1842	1.3327	3.73E-14
10	122452080	rs11200633	LOC105378525		0.4299	C	T	T	1.2557	0.0301	1.1837	1.3320	4.02E-14
10	122453397	rs61871746	ARMS2	LOC105378525	0.4414	T	C	C	1.2525	0.0298	1.1814	1.3280	4.50E-14
10	122454932	rs10490924	ARMS2	LOC105378525	0.4415	G	T	T	1.2521	0.0298	1.1810	1.3275	4.88E-14
10	122455084	rs61544945	ARMS2	LOC105378525	0.4408	G	GGT	GGT	1.2544	0.0299	1.1830	1.3300	3.31E-14
10	122455460	rs36212731	ARMS2	LOC105378525	0.4412	G	T	T	1.2526	0.0299	1.1814	1.3281	4.57E-14
10	122455682	rs36212732	ARMS2	LOC105378525	0.4414	A	G	G	1.2525	0.0298	1.1814	1.3280	4.50E-14
10	122455695	rs36212733	ARMS2	LOC105378525	0.4414	T	C	C	1.2525	0.0298	1.1814	1.3280	4.50E-14
10	122455799	rs3750848	ARMS2	LOC105378525	0.4414	T	G	G	1.2526	0.0298	1.1814	1.3280	4.43E-14
10	122455905	rs3750847	ARMS2	LOC105378525	0.4413	C	T	T	1.2521	0.0298	1.1809	1.3275	5.02E-14
10	122456049	rs3750846	ARMS2	LOC105378525	0.4448	T	C	C	1.2438	0.0299	1.1731	1.3187	2.72E-13
10	122458241	rs5788557	LOC105378525		0.4816	G	GC	G	0.8315	0.0301	0.7839	0.8820	8.73E-10
10	122459759	rs3793917	HTRA1	LOC105378525	0.4491	C	G	G	1.2378	0.0298	1.1676	1.3121	7.90E-13
10	122460545	rs3763764	HTRA1	LOC105378525	0.4486	A	G	G	1.2414	0.0299	1.1709	1.3162	4.36E-13
10	122461028	rs11200638	HTRA1	LOC105378525	0.4485	G	A	A	1.2362	0.0298	1.1661	1.3106	1.14E-12
10	122461754	rs1049331	HTRA1	LOC105378525	0.4463	C	T	T	1.2340	0.0300	1.1636	1.3088	2.40E-12
10	122467114	rs2284665	HTRA1		0.4826	G	T	T	1.1923	0.0297	1.1247	1.2638	3.39E-09
10	122470508	rs58077526	HTRA1		0.4718	A	C	C	1.2052	0.0313	1.1335	1.2815	2.53E-09
10	122471234	rs2672592	HTRA1		0.4717	G	T	T	1.2053	0.0313	1.1335	1.2817	2.50E-09
10	122471948	rs932275	HTRA1		0.4753	G	A	A	1.1927	0.0313	1.1217	1.2683	1.86E-08
10	122474521	rs2142308	HTRA1		0.4704	G	C	C	1.1966	0.0317	1.1245	1.2733	1.51E-08
