# Supplementary material for: Identification of Genetic Variants for Risk Prediction and Early Diagnosis of Age-Related Macular Degeneration in the Taiwanese Population
Source: Int J Mol Sci. 2024 Mar 12;25(6):3230. doi: 10.3390/ijms25063230 (PMC10970312; doi:10.3390/ijms25063230)
Supplement: Supplementary file 1 [file ijms-25-03230-s001.zip › sTable 2_R2.pdf]

Supplementary Table 2. List of the 1,412 SNPs in model 2

SNP	Gene	Chromosome	Position	MAF	REF allele	OR (95% CI)	P
rs10864268	CAMTA1	1	7037762	0.0852	C	0.81 (0.73, 0.91)	2.71E-04
rs374249490	PRAMEF12	1	12778685	0.0033	C	2.27 (1.48, 3.5)	1.93E-04
rs370129192	PDPN;PRDM2	1	13670726	0.0034	G	2.27 (1.5, 3.44)	1.15E-04
rs1376680374	KAZN	1	14598924	0.0007	T	5.6 (2.35, 13.34)	9.95E-05
rs188540402	IGSF21	1	18266275	0.0021	C	2.7 (1.58, 4.62)	2.79E-04
rs191054859	IGSF21	1	18374849	0.0007	C	5.36 (2.2, 13.04)	2.13E-04
rs184153530	IGSF21;KLHDC7A	1	18465571	0.002	G	2.98 (1.75, 5.07)	5.69E-05
rs10914812	CSMD2	1	33906522	0.2569	T	0.87 (0.81, 0.93)	8.90E-05
rs556307125	LINC01685;RRAGC	1	38758279	0.0008	A	4.65 (2.02, 10.72)	3.13E-04
rs577630151	CLDN19	1	42740486	0.0015	G	3.61 (1.95, 6.67)	4.26E-05
rs1040176899	KIF2C	1	44741558	0.0016	G	3.06 (1.69, 5.54)	2.35E-04
rs1230535468	ZSWIM5	1	45022031	0.0016	A	3.16 (1.76, 5.68)	1.22E-04
rs145201929	GPBP1L1	1	45644819	0.0023	A	2.68 (1.62, 4.42)	1.17E-04
rs998726	LINC01398;DMBX1	1	46488618	0.0031	G	2.23 (1.44, 3.46)	3.42E-04
rs12408243	DMBX1;MKNK1-AS1	1	46524372	0.0016	G	3.04 (1.69, 5.47)	2.00E-04
rs557222502	ATPAF1	1	46659059	0.0027	C	2.5 (1.57, 3.97)	1.04E-04
rs537242305	NRDC	1	51792693	0.0009	C	4.27 (1.99, 9.14)	1.89E-04
rs148882441	GLIS1	1	53707615	0.0104	A	1.62 (1.25, 2.09)	2.42E-04
rs372925430	DHCR24;TMEM61	1	54966794	0.0007	G	6.45 (2.54, 16.37)	8.87E-05
rs997838858	PLPP3	1	56508339	0.0023	C	2.54 (1.55, 4.15)	2.01E-04
rs79251419	PLPP3	1	56554831	0.048	T	0.76 (0.65, 0.88)	2.40E-04
rs1003329891	DAB1	1	57759829	0.0011	A	4.04 (2, 8.18)	1.03E-04
rs1179571417	KANK4	1	62311353	0.0005	T	7.38 (2.47, 22.03)	3.38E-04
rs965003793	PGM1	1	63618906	0.003	T	2.28 (1.45, 3.57)	3.30E-04
rs143212365	MIER1	1	66948850	0.0047	G	2 (1.39, 2.89)	2.09E-04
rs527501881	GNG12-AS1	1	67903472	0.0065	C	1.8 (1.31, 2.46)	2.66E-04
rs1036400128	LRRC40	1	70161379	0.0006	C	6.02 (2.28, 15.89)	2.85E-04
rs79278276	PIGK	1	77132301	0.0411	T	1.32 (1.15, 1.52)	1.20E-04
rs974540608	AK5	1	77347129	0.0013	C	3.75 (1.94, 7.25)	8.85E-05
rs1383885905		1	78543522	0.0021	TG	2.69 (1.58, 4.56)	2.52E-04
rs1035178231	IFI44;ADGRL4	1	78801318	0.0004	C	12.15 (3.27, 45.12)	1.91E-04
rs150351343	LOC124904203	1	78875400	0.0004	C	11.26 (3.11, 40.74)	2.24E-04
rs993015029	ADGRL4;LINC01781	1	80078460	0.0008	T	5.49 (2.3, 13.09)	1.23E-04
rs975928498	LOC101927434;ADGRL2	1	81626186	0.0013	C	3.61 (1.87, 6.98)	1.30E-04
rs143135395	LOC101927844	1	87211824	0.0006	ATCT	6.18 (2.32, 16.48)	2.74E-04

rs1447221807	DPYD	1	97293979	0.0006	C	6.84 (2.49, 18.74)	1.87E-04
rs376102760	LINC01307;OLFM3	1	101724153	0.0144	A	0.57 (0.42, 0.77)	2.45E-04
rs192454591	AMY1C;LOC100129138	1	103961904	0.0105	T	1.71 (1.33, 2.21)	3.52E-05
rs5776715	LOC100129138;LINC01676	1	104244550	0.461	A	1.12 (1.06, 1.19)	2.03E-04
rs372311996	LOC100129138;LINC01676	1	104564612	0.0012	T	3.68 (1.86, 7.29)	1.77E-04
rs76046454	LINC01661;PRMT6	1	106963916	0.1178	G	0.83 (0.75, 0.91)	9.88E-05
rs1395877988	VAV3	1	107603995	0.0023	G	2.67 (1.62, 4.39)	1.14E-04
rs567770705	VAV3	1	107702734	0.0103	C	1.66 (1.28, 2.14)	1.10E-04
rs139605722	SLC6A17	1	110161076	0.0027	G	2.48 (1.55, 3.98)	1.59E-04
rs779388073	ADORA3	1	111500568	0.002	AAG	2.83 (1.64, 4.88)	1.84E-04
rs550905253	SLC22A15	1	116039540	0.0015	A	3.23 (1.76, 5.91)	1.49E-04
chr1:116171457_CT_C		1	116171457	0.0013	CT	4.13 (2.17, 7.88)	1.64E-05
rs113594400	VTCN1	1	117199776	0.0122	C	1.63 (1.29, 2.07)	5.17E-05
rs79906727	NBPF10;NBPF19;NBPF20	1	146014328	0.1282	G	1.18 (1.08, 1.29)	2.76E-04
rs61838383	BCL9;NBPF19	1	147581640	0.0489	T	1.28 (1.12, 1.46)	2.46E-04
rs775872302	TCHH	1	152107890	0.0006	T	6.95 (2.51, 19.27)	1.92E-04
rs184271681	S100A8	1	153403428	0.0021	T	2.91 (1.72, 4.9)	6.36E-05
rs370693008	S100A7A;S100A7L2	1	153429190	0.0018	C	2.82 (1.6, 4.96)	3.20E-04
rs7416852	ETV3;FCRL5	1	157154991	0.0874	C	0.81 (0.72, 0.9)	1.52E-04
rs5778138	TAGLN2	1	159925227	0.0329	TCTATG	1.4 (1.21, 1.63)	1.18E-05
rs7543462	IGSF9	1	159929123	0.2849	A	0.88 (0.83, 0.95)	3.42E-04
rs142246009	ITLN2;F11R	1	160980122	0.0026	G	2.59 (1.6, 4.18)	1.05E-04
rs16844951	DDR2	1	162776911	0.0158	A	1.59 (1.29, 1.96)	1.32E-05
rs575167842	LOC100422212;PBX1	1	164026923	0.0028	G	2.44 (1.54, 3.86)	1.31E-04
rs182241756	LOC100422212;PBX1	1	164329756	0.0029	G	2.49 (1.59, 3.91)	6.93E-05
rs943361204	MPZL1	1	167753801	0.0008	C	6.52 (2.75, 15.42)	1.99E-05
rs139709403	ADCY10	1	167863117	0.0013	T	3.59 (1.85, 6.99)	1.66E-04
rs369335390	TBX19;MIR557	1	168327957	0.0007	C	5.78 (2.4, 13.9)	8.97E-05
rs141782354	DNM3	1	172145864	0.0173	A	1.45 (1.19, 1.78)	2.90E-04
rs894453717	LOC100506023	1	173413269	0.0012	G	3.86 (1.99, 7.51)	6.71E-05
rs1195976562	ANKRD45	1	173707347	0.0013	T	3.36 (1.77, 6.38)	2.13E-04
rs1238859241	RABGAP1L	1	174216986	0.0013	G	3.39 (1.78, 6.43)	1.90E-04
rs191227463	RABGAP1L	1	174563101	0.0013	A	3.38 (1.78, 6.41)	1.99E-04
rs182501051	RABGAP1L	1	174850265	0.0013	G	3.35 (1.77, 6.37)	2.17E-04
rs975444687	TNR	1	175632508	0.0019	A	3.09 (1.77, 5.4)	7.58E-05
rs505189	TNR;LINC01657	1	175874349	0.172	T	1.16 (1.07, 1.25)	1.49E-04

rs546384550	LINC01657;RFWD2	1	175884344	0.0026	C	2.92 (1.81, 4.72)	1.17E-05
rs532789969	RFWD2;PAPPA2	1	176248923	0.0029	A	2.8 (1.79, 4.39)	7.21E-06
rs190653087	PAPPA2	1	176613810	0.0011	G	4.23 (2.08, 8.63)	7.15E-05
rs781672360	LINC01645;LINC01741	1	177504129	0.0009	AAAAAT	4.32 (1.99, 9.37)	2.08E-04
rs2149184	LINC01741;SEC16B	1	177874453	0.1671	A	0.86 (0.79, 0.93)	2.38E-04
rs146248695	LINC01720;LINC01680	1	190939347	0.0078	T	1.77 (1.33, 2.37)	1.13E-04
rs930736020	LINC01724	1	196050632	0.0051	T	1.9 (1.34, 2.7)	3.17E-04
rs559350	CFH	1	196673403	0.4166	T	0.89 (0.84, 0.94)	1.29E-04
rs193058059	LHX9;NEK7	1	197987886	0.0032	G	2.23 (1.44, 3.44)	3.28E-04
rs117442436	MIR181A1HG	1	198843512	0.005	T	2.11 (1.49, 3)	3.18E-05
chr1:205573248_TA_T		1	205573248	0.0192	TA	1.46 (1.2, 1.78)	1.52E-04
rs1204676	C1orf132;CD34	1	207877502	0.3666	C	0.88 (0.82, 0.93)	4.91E-05
rs151291037	LOC105372892	1	208682820	0.1851	T	1.15 (1.07, 1.24)	1.83E-04
rs59300965	FLVCR1;VASH2	1	212922142	0.092	A	1.2 (1.09, 1.32)	2.41E-04
rs574681965	LINC01655;HHIPL2	1	221962431	0.0049	G	1.99 (1.39, 2.85)	1.78E-04
rs535293320	CNIH3	1	224714516	0.0013	G	3.42 (1.78, 6.58)	2.20E-04
chr1:226689567_A_AT T		1	226689567	0.0154	A	1.5 (1.21, 1.87)	2.74E-04
rs191412950	PRSS38;WNT9A	1	227908170	0.0048	C	2.02 (1.41, 2.9)	1.29E-04
rs3795777	OBSCN	1	228216967	0.3791	T	1.12 (1.06, 1.19)	1.61E-04
rs117999377	FAM89A;TRIM67	1	231065795	0.0355	C	1.34 (1.16, 1.56)	1.24E-04
rs117946086	KCNK1;SLC35F3	1	233844297	0.0066	C	1.83 (1.33, 2.5)	1.72E-04
rs1370442201	LINC02768	1	235948707	0.0026	C	2.7 (1.69, 4.31)	3.03E-05
rs10639946	WDR64;EXO1	1	241843845	0.2697	T	1.13 (1.06, 1.21)	2.80E-04
rs897065	DCDC2C	2	3844459	0.1268	A	0.84 (0.77, 0.92)	2.58E-04
rs536724882	LINC01247;LINC01246	2	6609450	0.0014	T	4.2 (2.22, 7.93)	9.85E-06
rs185722877	RNF144A	2	7031180	0.0029	C	2.31 (1.46, 3.65)	3.48E-04
rs1266813109		2	11986058	0.0019	C	2.94 (1.72, 5.04)	8.69E-05
rs188141736	MIR3681HG	2	12491601	0.0078	C	1.8 (1.34, 2.43)	9.60E-05
rs868035092	MIR3681HG;TRIB2	2	12668296	0.0007	C	5.35 (2.18, 13.08)	2.41E-04
rs191085153	FAM84A;NBAS	2	15008846	0.0037	C	2.16 (1.44, 3.25)	2.20E-04
rs76112867	FAM49A	2	16605096	0.066	G	0.78 (0.69, 0.89)	1.50E-04
rs139141129	FAM49A	2	16663242	0.0007	A	5.52 (2.21, 13.75)	2.47E-04
rs146224775	WDR35	2	19947812	0.0056	C	1.93 (1.38, 2.7)	1.31E-04
rs190018045	LINC01884;KLHL29	2	22978958	0.0006	T	7.17 (2.66, 19.36)	1.01E-04
rs1444883269	KLHL29	2	23384988	0.0006	A	7.03 (2.58, 19.2)	1.41E-04
rs1416279685	KLHL29	2	23565132	0.001	A	4.41 (2.1, 9.28)	9.23E-05
rs183263679	RAB10	2	26103215	0.0009	A	5.68 (2.6, 12.43)	1.35E-05

rs184571661	FOSL2;PLB1	2	28453602	0.0049	G	2.02 (1.4, 2.9)	1.71E-04
rs188404706	ALK	2	29605230	0.0056	C	1.93 (1.37, 2.72)	1.78E-04
rs554307464	SPAST	2	32068424	0.0053	C	1.94 (1.37, 2.75)	2.03E-04
rs1416184405	LTBP1	2	33141079	0.0016	C	3.95 (2.21, 7.08)	3.76E-06
rs952202261	RASGRP3	2	33517608	0.0015	T	3.86 (2.1, 7.1)	1.43E-05
rs12465135	LINC01317	2	33792363	0.0116	C	1.59 (1.24, 2.04)	2.52E-04
rs977165500	LINC01317	2	33802991	0.0014	G	3.91 (2.08, 7.36)	2.36E-05
rs199813503	LINC01317	2	34119041	0.0032	A	2.38 (1.54, 3.68)	9.54E-05
rs1333061114		2	34648677	0.0008	C	4.3 (1.93, 9.56)	3.47E-04
rs553524254	LINC01320;MIR548AD	2	34808318	0.002	C	2.97 (1.74, 5.08)	6.61E-05
rs13407474	LINC01320;MIR548AD	2	34970450	0.1772	A	1.16 (1.07, 1.25)	1.57E-04
rs77484844	SLC8A1-AS1	2	40224838	0.0028	T	2.39 (1.51, 3.8)	2.22E-04
rs79545581	SLC8A1	2	40300069	0.054	T	0.74 (0.64, 0.86)	4.69E-05
rs1242724785	HAAO	2	42794304	0.002	CTA	2.74 (1.6, 4.68)	2.25E-04
rs147248056	THADA	2	43465403	0.0012	TAG	3.69 (1.86, 7.33)	1.85E-04
rs577417028	PRKCE	2	46126786	0.0006	G	7.65 (2.72, 21.56)	1.18E-04
rs192364734	TTC7A	2	47025428	0.0025	C	2.45 (1.51, 3.98)	2.94E-04
rs62138456	LOC101927043;EPCAM	2	47352558	0.3967	A	1.12 (1.06, 1.19)	1.62E-04
rs185740041	FSHR;NRXN1	2	49669651	0.001	C	3.89 (1.88, 8.06)	2.51E-04
rs576923103	FSHR;NRXN1	2	49844293	0.0042	C	2.12 (1.45, 3.1)	1.11E-04
rs117764746	NRXN1	2	50326109	0.0193	C	1.43 (1.18, 1.74)	2.94E-04
rs555896324	NRXN1	2	50585675	0.0044	A	2.06 (1.4, 3.04)	2.47E-04
rs138832492	LOC730100	2	51967122	0.0164	C	1.48 (1.19, 1.83)	3.38E-04
rs192702848	LOC730100	2	52276773	0.0015	A	3.22 (1.77, 5.88)	1.37E-04
rs555713327	MIR4431;ASB3	2	52751542	0.0014	G	3.4 (1.82, 6.35)	1.27E-04
rs559981980	MIR4431;ASB3	2	53093052	0.0013	T	3.44 (1.8, 6.58)	1.89E-04
rs189977943	MIR4431;ASB3	2	53332400	0.0029	T	2.45 (1.56, 3.85)	1.02E-04
rs528382522	MIR4431;ASB3	2	53643767	0.0089	TAAGGCCCCTC	1.67 (1.26, 2.2)	3.15E-04
rs1156525339	EML6	2	54901939	0.0007	A	5.55 (2.2, 14.01)	2.91E-04
rs144606047	FANCL	2	58229587	0.0047	T	2.1 (1.46, 3.03)	6.39E-05
rs17398871	FANCL;LINC01795	2	58254454	0.0535	T	1.27 (1.12, 1.44)	1.46E-04
rs10153919	LINC01122	2	59039036	0.0407	A	1.29 (1.12, 1.48)	3.50E-04
rs12621925	LINC01122	2	59050864	0.0017	G	3 (1.66, 5.39)	2.55E-04
rs181759988	COMMD1	2	61949984	0.0057	A	1.91 (1.36, 2.67)	1.57E-04
rs182786005	B3GNT2;TMEM17	2	62419650	0.0082	C	0.44 (0.29, 0.68)	2.27E-04
rs6546024	MDH1;UGP2	2	63694641	0.1385	A	1.2 (1.1, 1.3)	1.75E-05
rs147844684	LOC100507006;MIR4433B	2	64273182	0.034	TCTCTCA	1.34 (1.15, 1.56)	1.77E-04

rs142837218	FBXO48	2	68465360	0.007	A	1.85 (1.35, 2.53)	1.14E-04
rs138993544	LOC105374820;REG3G	2	78941173	0.0006	C	6.24 (2.41, 16.17)	1.63E-04
rs975042209	SH2D6;PARTICL	2	85469159	0.0007	G	6.24 (2.54, 15.31)	6.54E-05
rs933047167		2	89790826	0.0011	G	4.03 (1.99, 8.17)	1.13E-04
rs1253929310		2	91571649	0.0008	C	4.58 (2.02, 10.41)	2.77E-04
rs554880152	LOC442028	2	94879855	0.0009	C	4.67 (2.16, 10.1)	9.32E-05
rs146965327	ZNF2;PROM2	2	95188593	0.0012	A	3.93 (1.97, 7.85)	1.05E-04
rs191977333	TRIM43;LINC00342	2	95685930	0.0012	A	3.64 (1.84, 7.2)	1.98E-04
rs535060119	GPAT2;ADRA2B	2	96086141	0.0014	G	4.28 (2.27, 8.05)	6.56E-06
rs10630400	KIAA1211L;TSGA10	2	98958825	0.3136	C	0.89 (0.83, 0.95)	2.56E-04
rs994452892	LONRF2	2	100300942	0.0048	T	1.99 (1.39, 2.84)	1.56E-04
rs35451970	NCK2;C2orf40	2	106043267	0.0637	C	1.25 (1.11, 1.41)	2.81E-04
rs10191974	UXS1;PLGLA	2	106364301	0.4247	G	1.12 (1.05, 1.19)	2.27E-04
rs144966496	IL1B;IL37	2	112859244	0.0628	C	0.78 (0.68, 0.89)	1.39E-04
rs141996375	DPP10	2	115267906	0.0077	C	1.75 (1.31, 2.35)	1.86E-04
rs749076220	CCDC93	2	117945527	0.0011	A	3.85 (1.9, 7.8)	1.87E-04
rs12615912	LOC100506797;LOC101927709	2	118332517	0.017	A	1.47 (1.19, 1.8)	3.20E-04
rs182305045	MARCO;C1QL2	2	119054987	0.0105	A	1.72 (1.33, 2.22)	3.27E-05
rs118030760	GLI2	2	120974220	0.0053	C	1.89 (1.33, 2.68)	3.45E-04
rs13400176	TFCP2L1;CLASP1	2	121300486	0.1704	A	1.16 (1.07, 1.25)	1.90E-04
rs113804966	CLASP1	2	121590936	0.0376	C	1.31 (1.14, 1.51)	1.90E-04
rs75728942	TSN;LINC01826	2	121844944	0.0562	C	1.26 (1.12, 1.42)	1.26E-04
rs559099680	TSN;LINC01826	2	121884081	0.0068	C	1.84 (1.35, 2.49)	9.94E-05
rs951757432	TSN;LINC01826	2	122540351	0.0005	C	9.81 (3.08, 31.25)	1.13E-04
rs137950356	CNTNAP5;LINC01941	2	125812307	0.0427	C	0.74 (0.63, 0.86)	1.53E-04
rs555396752	LINC01854;PLAC9P1	2	129541013	0.001	C	5 (2.3, 10.89)	4.89E-05
rs376830223	NXPH2	2	138686438	0.0022	C	2.59 (1.55, 4.33)	3.00E-04
rs10192367	YY1P2;LRP1B	2	139557147	0.0062	C	1.82 (1.32, 2.52)	2.90E-04
rs1464849936		2	140060991	0.0009	A	4.71 (2.2, 10.07)	6.62E-05
rs75704924	GTDC1	2	144301194	0.0254	T	1.42 (1.2, 1.69)	6.17E-05
rs182060408	LINC01412;TEX41	2	144611993	0.0034	G	2.24 (1.46, 3.44)	2.16E-04
rs576463804	PABPC1P2;ACVR2A	2	146905290	0.0008	C	4.56 (1.99, 10.46)	3.44E-04
rs72985321	LYPD6B;LYPD6	2	149233512	0.0021	A	2.67 (1.57, 4.55)	3.04E-04
rs186118920	ARL6IP6;RPRM	2	152791569	0.0007	T	6.03 (2.45, 14.83)	9.07E-05
rs372822740	DPP4	2	162010628	0.0015	G	3.14 (1.69, 5.84)	2.99E-04
rs1431298411	KCNH7	2	162642703	0.0008	A	6.39 (2.79, 14.64)	1.14E-05
rs1467510309	SCN2A	2	165325992	0.0005	GA	9.04 (2.74, 29.76)	2.95E-04

rs2288704	LOC100506124	2	165870717	0.1972	G	0.85 (0.79, 0.92)	5.24E-05
rs182701995	SCN7A;XIRP2	2	166797497	0.002	A	2.74 (1.6, 4.7)	2.41E-04
rs1421894825	XIRP2	2	166893553	0.0011	T	3.73 (1.86, 7.48)	2.09E-04
rs145463848	PPIG	2	169600006	0.0038	G	2.31 (1.55, 3.44)	3.62E-05
rs3843330	MYO3B	2	170269320	0.0337	C	1.37 (1.17, 1.6)	6.64E-05
rs181885532	METAP1D	2	172079692	0.0043	G	2.29 (1.56, 3.36)	2.23E-05
rs559777439	CDCA7;SP3	2	173822370	0.0008	T	4.86 (2.17, 10.89)	1.25E-04
rs981333289	LOC100130691	2	177318432	0.0038	C	2.12 (1.41, 3.18)	3.18E-04
rs377028457	CWC22;SCHLAP1	2	180292048	0.004	T	2.04 (1.39, 3.01)	3.14E-04
rs146533744	NUP35;MIR548AE1	2	183599585	0.0037	G	2.32 (1.55, 3.47)	3.87E-05
rs541752862	NUP35;MIR548AE1	2	183604587	0.0024	G	2.57 (1.57, 4.2)	1.75E-04
rs1233974660		2	183744742	0.0008	A	4.66 (2.07, 10.53)	2.10E-04
rs188139709	NUP35;MIR548AE1	2	183942882	0.0033	G	2.59 (1.7, 3.93)	9.02E-06
rs1257628084		2	184127109	0.0005	A	9.74 (2.94, 32.21)	1.93E-04
rs1015649799	NUP35;MIR548AE1	2	184291064	0.0033	G	2.66 (1.76, 4.04)	4.03E-06
rs187836995	MIR548AE1;ZNF804A	2	184545325	0.0028	A	2.45 (1.55, 3.88)	1.22E-04
rs192232822	ZNF804A	2	184717089	0.0037	C	2.15 (1.43, 3.24)	2.39E-04
rs191989741	ZNF804A;LOC105373782	2	185051948	0.0037	T	2.14 (1.42, 3.21)	2.51E-04
rs186205402	LOC105373782;LOC101927196	2	185230888	0.0026	T	2.42 (1.51, 3.87)	2.38E-04
rs11685570	NAB1;GLS	2	190864660	0.2586	C	0.87 (0.81, 0.93)	5.60E-05
rs1167246262		2	192346017	0.0045	T	2.03 (1.4, 2.94)	1.94E-04
rs72921507	TMEFF2;PCGEM1	2	192516605	0.0347	A	1.34 (1.15, 1.56)	1.98E-04
chr2:192703864_A_AAT		2	192703864	0.0064	A	2.09 (1.53, 2.85)	3.26E-06
rs181344336	PCGEM1;LINC01821	2	192988612	0.0052	C	2.18 (1.55, 3.05)	6.15E-06
rs201200558	PCGEM1;LINC01821	2	193578527	0.0088	GC	1.66 (1.27, 2.18)	2.64E-04
rs186274444	PCGEM1;LINC01821	2	194321175	0.0031	T	2.32 (1.5, 3.58)	1.55E-04
rs186616550	STK17B	2	196164321	0.0015	A	3.38 (1.84, 6.2)	8.81E-05
rs78070458	LINC01923;SATB2	2	198602850	0.0034	T	2.66 (1.74, 4.06)	5.99E-06
rs939171235	LINC01923;SATB2	2	198945310	0.0008	G	4.69 (2.01, 10.94)	3.41E-04
rs16839555	NBEAL1	2	203134759	0.0414	T	0.73 (0.62, 0.86)	1.34E-04
rs200933848	RAPH1;CD28	2	203552769	0.04	GTA	0.72 (0.61, 0.85)	1.28E-04
rs201894162	ICOS;PARD3B	2	204317259	0.0007	A	7.78 (2.99, 20.23)	2.57E-05
rs976973010	PARD3B	2	205169535	0.0005	A	9.08 (2.93, 28.2)	1.35E-04
rs925005162	LOC101927865	2	207249826	0.0042	A	2.11 (1.44, 3.08)	1.18E-04
rs1021312902	PTH2R	2	208392095	0.0021	G	2.71 (1.61, 4.55)	1.78E-04
rs182005735	ERBB4	2	212091630	0.0017	G	3.09 (1.74, 5.51)	1.25E-04

rs568957693	SPAG16	2	214166507	0.0009	A	4.31 (2, 9.3)	2.00E-04
rs554289614	LINC01921;DIRC3-AS1	2	217119925	0.0008	C	4.93 (2.11, 11.55)	2.36E-04
rs1430742354	CT75	2	222323236	0.0012	T	3.68 (1.87, 7.25)	1.68E-04
rs181835546	NYAP2;LOC646736	2	225676626	0.0026	A	2.73 (1.7, 4.39)	3.44E-05
rs200081671	NYAP2;LOC646736	2	225912638	0.0005	GAA	10.1 (3.17, 32.15)	9.14E-05
rs1013587807	NYAP2;LOC646736	2	226076875	0.0018	C	2.84 (1.61, 5.01)	3.00E-04
rs12104555	LOC646736;MIR5702	2	226511789	0.0871	G	1.23 (1.11, 1.36)	6.64E-05
chr2:231734685_T_TA		2	231734685	0.0536	T	1.29 (1.14, 1.47)	7.90E-05
rs11384415	LINC01107;TWIST2	2	238629479	0.2422	G	1.14 (1.07, 1.23)	1.92E-04
rs979227143	TWIST2	2	238896106	0.0005	C	7.93 (2.59, 24.24)	2.83E-04
rs1458523377	HDAC4	2	239064293	0.004	AT	2.29 (1.55, 3.38)	3.03E-05
rs4675852	CROCC2	2	240915765	0.2091	C	0.86 (0.8, 0.93)	1.71E-04
rs116918647	CNTN4	3	2853145	0.0016	T	3.06 (1.7, 5.51)	2.02E-04
rs1414663329	ITPR1	3	4728476	0.0009	G	4.19 (1.94, 9.08)	2.73E-04
rs568606393	BHLHE40;ARL8B	3	5080635	0.0016	A	3.12 (1.73, 5.62)	1.55E-04
rs928465142	MIR4790;GRM7-AS3	3	5674168	0.001	G	4.25 (2.01, 9.01)	1.58E-04
rs13069276	MIR4790;GRM7-AS3	3	6165377	0.0646	T	0.78 (0.69, 0.89)	1.72E-04
rs200452192	GRM7	3	7698035	0.0011	G	3.85 (1.88, 7.85)	2.14E-04
rs1181312955		3	7931745	0.0011	TA	3.7 (1.82, 7.52)	3.06E-04
rs190704756	RPUSD3;CIDEC	3	9861595	0.0023	C	2.81 (1.72, 4.6)	3.73E-05
rs573749665	CIDEC	3	9876264	0.0006	G	6.77 (2.47, 18.59)	2.05E-04
rs146881934	ATP2B2	3	10390670	0.0066	C	1.94 (1.43, 2.64)	2.18E-05
rs1346007444	ATP2B2	3	10402472	0.0023	T	2.57 (1.57, 4.23)	1.92E-04
rs181316065	HRH1	3	11220305	0.0021	C	2.65 (1.56, 4.5)	3.14E-04
rs572643236	RAF1	3	12645743	0.0081	G	1.74 (1.31, 2.31)	1.32E-04
rs189434319	CAND2	3	12833369	0.0009	A	5.1 (2.39, 10.89)	2.60E-05
rs559649121	LINC02022;IQSEC1	3	12891796	0.0018	C	2.8 (1.6, 4.91)	3.24E-04
rs62241794	FGD5	3	14924716	0.0025	C	2.62 (1.61, 4.27)	1.04E-04
rs74827146	LOC339862	3	18022053	0.191	T	0.85 (0.78, 0.92)	7.55E-05
chr3:18582952_G_GTA		3	18582952	0.0015	G	3.1 (1.71, 5.63)	2.07E-04
rs1042871864	LOC105376988	3	21220289	0.0008	G	4.75 (2.05, 11.02)	2.80E-04
rs371721587	ZNF385D	3	21462596	0.0034	CAAATT	2.37 (1.55, 3.63)	7.27E-05
rs79008507	ZNF385D;ZNF385D-AS2	3	21856999	0.0904	C	1.2 (1.09, 1.33)	3.49E-04
rs185458857	RARB	3	25297719	0.017	C	1.48 (1.2, 1.83)	2.50E-04
rs1465370019		3	26251856	0.0009	A	4.16 (1.91, 9.04)	3.25E-04
rs1357036066		3	28199200	0.0022	C	2.77 (1.67, 4.59)	7.64E-05
rs1169459560		3	29117552	0.0024	A	2.98 (1.84, 4.83)	9.57E-06

rs1215000210	RBMS3	3	29477580	0.0017	TTATCAC	3.16 (1.78, 5.59)	8.03E-05
rs1415391123	RBMS3	3	29754606	0.0015	C	3.71 (2.01, 6.84)	2.63E-05
rs5848583	ZNF621;CTNNB1	3	40972208	0.2671	AG	0.88 (0.82, 0.94)	1.68E-04
rs185962487	ABHD5;MIR138-1	3	44104330	0.0103	C	1.8 (1.4, 2.33)	6.05E-06
rs141434004	LIMD1	3	45674741	0.0016	C	3.13 (1.74, 5.64)	1.43E-04
rs9874534	CACNA2D3	3	54298343	0.0834	G	0.8 (0.71, 0.9)	1.17E-04
rs9843369	C3orf67;FHIT	3	59426263	0.2113	C	1.15 (1.07, 1.23)	9.39E-05
rs186133451	CADPS;LINC00698	3	62917130	0.0008	A	4.7 (2.03, 10.84)	2.91E-04
rs570346181	SNTN	3	63654631	0.0018	T	3.14 (1.79, 5.5)	6.34E-05
rs62260081	LRIG1;LOC105377143	3	66650453	0.0141	G	1.53 (1.21, 1.92)	3.31E-04
rs181138260	FAM19A1;FAM19A4	3	68725607	0.0063	G	1.87 (1.36, 2.58)	1.19E-04
rs9873880	LINC00870;RYBP	3	72272764	0.0178	G	1.47 (1.2, 1.8)	1.92E-04
rs534010314	GXYLT2	3	72970179	0.002	C	2.85 (1.67, 4.87)	1.29E-04
rs185613296	ZNF717;ROBO2	3	76177760	0.0034	C	2.54 (1.69, 3.82)	7.52E-06
rs932231173	ROBO2	3	77051809	0.0018	A	3.1 (1.78, 5.42)	7.03E-05
rs148166712	LINC02077;ROBO1	3	78132024	0.0037	C	2.18 (1.45, 3.29)	1.98E-04
rs575194326	CADM2	3	85085816	0.0007	C	5.54 (2.23, 13.77)	2.29E-04
rs1046513791	CADM2	3	85635911	0.0005	G	9.99 (3.01, 33.1)	1.67E-04
rs373758597	CADM2;LINC02070	3	86276090	0.0014	T	3.61 (1.9, 6.87)	8.92E-05
rs532266623	POU1F1;HTR1F	3	87578726	0.0044	C	2.13 (1.46, 3.1)	8.96E-05
rs9826048	EPHA3;NONE	3	90342352	0.0977	C	1.21 (1.1, 1.33)	6.72E-05
rs1457456670		3	90635346	0.0073	C	1.76 (1.29, 2.39)	3.23E-04
rs4241462	NSUN3;MIR6730	3	94248362	0.4641	A	0.88 (0.83, 0.94)	6.59E-05
chr3:94758124_A_AT		3	94758124	0.075	A	1.24 (1.11, 1.39)	1.34E-04
chr3:94782247_T_G		3	94782247	0.001	T	3.89 (1.87, 8.09)	2.71E-04
rs548109807	LINC00879;MTHFD2P1	3	95189411	0.001	T	4.14 (1.97, 8.72)	1.79E-04
rs1401041243		3	95842337	0.0011	T	3.82 (1.88, 7.74)	2.02E-04
rs1479800541		3	96243575	0.0012	T	3.87 (1.95, 7.7)	1.15E-04
rs549598748	EPHA6	3	97176619	0.0033	T	2.26 (1.47, 3.46)	2.03E-04
rs148230175	EPHA6	3	97506342	0.0174	C	1.47 (1.19, 1.81)	2.87E-04
rs576470232	EPHA6	3	97550707	0.0023	A	2.61 (1.57, 4.32)	2.05E-04
rs146462314	OR5H1;OR5H14	3	98145900	0.0222	C	0.64 (0.51, 0.81)	1.32E-04
rs1460198539	ABI3BP	3	100962583	0.0017	C	3.17 (1.78, 5.62)	8.39E-05
rs190868678	CBLB;LINC00882	3	106105464	0.0011	C	3.85 (1.86, 7.98)	2.92E-04
rs139434587	LINC00636;CD47	3	107948429	0.0008	G	5.74 (2.47, 13.31)	4.76E-05
rs62281714	MIR4445;NECTIN3-AS1	3	109738791	0.469	A	0.89 (0.84, 0.94)	1.04E-04
rs576785664	MIR4445;NECTIN3-AS1	3	110002388	0.0019	T	3.46 (2, 5.98)	8.33E-06
rs147178107	BTLA	3	112483796	0.0569	C	1.28 (1.14, 1.45)	4.41E-05

rs148135620	LINC00901	3	116929433	0.0034	T	2.35 (1.53, 3.6)	8.58E-05
rs186258146	LOC105374060	3	118586651	0.0018	T	2.95 (1.69, 5.13)	1.32E-04
rs77848254	PDIA5	3	123120393	0.005	G	1.94 (1.36, 2.77)	2.53E-04
rs1042959982	SEC22A	3	123221676	0.0007	C	6.01 (2.41, 14.99)	1.20E-04
rs80177467	KALRN	3	124615214	0.0035	G	2.2 (1.45, 3.34)	2.28E-04
rs370276689	ALDH1L1-AS2;KLF15	3	126303638	0.0032	G	2.38 (1.53, 3.71)	1.29E-04
rs139689605	NUP210P1	3	126671600	0.0035	GGAGA	2.4 (1.58, 3.63)	3.82E-05
rs911067503	PLXNA1	3	126991323	0.0035	A	2.3 (1.51, 3.48)	9.13E-05
rs73860728	MGLL;KBTBD12	3	127918378	0.3644	G	0.88 (0.83, 0.94)	1.39E-04
rs7622508	COL6A6	3	130597020	0.002	A	2.95 (1.72, 5.07)	8.97E-05
rs535391275	NEK11	3	131037015	0.0017	G	3.16 (1.75, 5.71)	1.38E-04
rs371439882	TMEM108	3	133112185	0.0012	TAAG	4.19 (2.1, 8.34)	4.61E-05
rs578195550	STAG1;SLC35G2	3	136812494	0.0019	C	2.91 (1.67, 5.07)	1.54E-04
rs192174742	NMNAT3;CLSTN2	3	139786172	0.0035	G	2.57 (1.71, 3.87)	5.65E-06
rs1092540	NMNAT3;CLSTN2	3	139883178	0.2494	C	1.15 (1.07, 1.23)	5.49E-05
rs1895857	CLSTN2	3	140020534	0.1715	C	0.85 (0.78, 0.92)	6.88E-05
rs1486397930	CLSTN2	3	140168620	0.0018	A	3.06 (1.75, 5.37)	9.18E-05
rs1195941171	SLC25A36	3	140966560	0.0014	AG	3.41 (1.83, 6.34)	1.05E-04
rs77217684	LINC02046;AGTR1	3	148489531	0.0281	A	0.66 (0.54, 0.81)	5.63E-05
rs140296896	LOC105374313;LINC01213	3	150178567	0.0597	G	0.78 (0.68, 0.89)	2.06E-04
rs534481542	CLRN1	3	150951906	0.0025	C	2.49 (1.53, 4.04)	2.26E-04
rs117766209	MED12L;P2RY12	3	151379595	0.0833	T	0.79 (0.71, 0.89)	9.55E-05
rs78333570	ARHGEF26	3	154153794	0.0381	T	1.35 (1.17, 1.56)	5.25E-05
rs1223423413	KCNAB1	3	156298970	0.0015	G	3.03 (1.66, 5.55)	3.12E-04
rs6777084	KCNAB1	3	156441143	0.0601	A	1.25 (1.11, 1.41)	2.77E-04
rs74871741	LINC01192;MIR1263	3	163959197	0.0233	G	0.65 (0.52, 0.82)	1.72E-04
chr3:164415454_G_GAT		3	164415454	0.2966	G	0.88 (0.82, 0.94)	3.01E-04
rs140917332	MECOM	3	169588726	0.0017	C	3.33 (1.88, 5.89)	3.70E-05
rs577491699	LINC00578	3	177459650	0.0042	C	2.04 (1.39, 2.98)	2.41E-04
rs1197784012		3	180139822	0.0011	G	3.77 (1.87, 7.59)	1.98E-04
rs1271411875		3	180406033	0.0011	C	3.7 (1.84, 7.42)	2.34E-04
rs145734693	CCDC39	3	180636853	0.0041	G	2.04 (1.38, 2.99)	3.05E-04
rs564343831	LINC01206;LINC01994	3	182367579	0.0015	C	3.09 (1.68, 5.68)	2.93E-04
rs116961268	KLHL24	3	183650946	0.0155	A	0.6 (0.45, 0.79)	3.11E-04
rs541818653	VPS8;C3orf70	3	185059645	0.002	C	2.71 (1.57, 4.67)	3.45E-04
rs149334470	LIPH	3	185526552	0.0029	G	2.48 (1.59, 3.87)	6.10E-05

rs1437726692	LIPH	3	185531571	0.0007	AAAG	5.72 (2.37, 13.8)	1.03E-04
rs181680901	LPP	3	188329623	0.0039	T	2.17 (1.44, 3.25)	1.86E-04
rs58700021	TP63	3	189746822	0.4238	C	0.89 (0.84, 0.95)	1.39E-04
rs13071108	IL1RAP	3	190637862	0.0155	T	1.48 (1.19, 1.83)	3.17E-04
rs138061058	IL1RAP;GMNC	3	190767388	0.0007	G	7.74 (2.96, 20.24)	3.05E-05
rs144853607	SENP5	3	196886330	0.0032	TACC	2.34 (1.51, 3.63)	1.53E-04
rs570304016	CPLX1	4	818551	0.0015	G	3.39 (1.84, 6.25)	8.86E-05
chr4:1249244_T_TGCGGGGCGGG		4	1249244	0.0124	T	0.5 (0.35, 0.7)	6.96E-05
rs28848271	RNF4;FAM193A	4	2552430	0.3917	C	1.13 (1.07, 1.2)	3.71E-05
rs550235472	WDR1	4	10080228	0.0009	C	4.95 (2.23, 10.97)	8.39E-05
rs182676930	NCAPG	4	17819442	0.0047	G	2.07 (1.43, 2.98)	9.77E-05
rs75564127	KCNIP4	4	21039349	0.0014	G	3.2 (1.71, 6)	2.87E-04
rs138798566	KCNIP4	4	21930384	0.0107	C	1.66 (1.29, 2.14)	7.33E-05
rs6824406	SEPSECS	4	25131443	0.0154	T	1.58 (1.27, 1.97)	3.36E-05
rs1340256685		4	27047433	0.0031	T	2.54 (1.65, 3.9)	2.09E-05
rs199760845	LINC02261	4	27225983	0.0079	C	1.73 (1.29, 2.31)	2.20E-04
rs183094472	LINC02497	4	31179083	0.012	A	1.56 (1.22, 1.98)	3.29E-04
rs117656989	LINC02353;LOC101928622	4	33233444	0.042	C	0.71 (0.6, 0.83)	3.18E-05
rs566516742	TBC1D1;LINC01258	4	38256500	0.0047	C	0.28 (0.14, 0.56)	3.26E-04
rs559751068	LINC01258	4	38431252	0.0041	G	2.16 (1.47, 3.18)	9.61E-05
rs935741777	KLF3-AS1	4	38613681	0.0004	T	11.07 (3.04, 40.35)	2.68E-04
rs180884901	FAM114A1	4	38900498	0.0023	T	2.67 (1.62, 4.4)	1.18E-04
rs13117988	KLHL5	4	39092219	0.4634	G	1.11 (1.05, 1.18)	3.03E-04
rs28665603	UBE2K;PDS5A	4	39811056	0.1861	C	1.18 (1.09, 1.27)	3.10E-05
rs368920177	RBM47;NSUN7	4	40721403	0.0022	TCTC	3.12 (1.88, 5.17)	1.13E-05
rs556858167	TMEM33;DCAF4L1	4	41972593	0.0006	A	5.76 (2.22, 14.96)	3.26E-04
rs150285457	SHISA3;ATP8A1	4	42407286	0.0106	AG	0.51 (0.35, 0.73)	2.63E-04
rs117095160	ATP8A1	4	42604383	0.0343	C	1.46 (1.26, 1.69)	6.55E-07
rs180775214	GABRA2;COX7B2	4	46589159	0.0009	A	5.86 (2.68, 12.84)	9.82E-06
rs541736876	SLAIN2	4	48341909	0.0007	C	6.5 (2.61, 16.14)	5.58E-05
rs75416085	LINC02283;LINC02260	4	54380331	0.0421	C	1.29 (1.13, 1.48)	2.04E-04
rs184778463	IGFBP7	4	57062025	0.0017	C	2.92 (1.62, 5.25)	3.44E-04
rs1237327347		4	59950120	0.0019	G	2.97 (1.72, 5.14)	9.66E-05
rs1284723677		4	60385103	0.0007	C	5.55 (2.22, 13.89)	2.53E-04
rs145324996	ADGRL3	4	61296854	0.0245	A	1.39 (1.17, 1.66)	2.36E-04
chr4:68249016_T_TTA		4	68249016	0.01	T	0.49 (0.34, 0.72)	2.47E-04

rs571179630	UGT2A3	4	68936039	0.0014	G	3.76 (2.03, 6.96)	2.49E-05
rs147758783		4	68954307	0.0019	C	3.06 (1.76, 5.3)	7.16E-05
rs567358839	UGT2B28;UGT2B4	4	69424967	0.0015	G	3.56 (1.95, 6.51)	3.58E-05
rs150509396	MUC7	4	70465603	0.0281	A	1.38 (1.17, 1.63)	1.51E-04
rs2332067	AMTN;AMBN	4	70580414	0.1855	C	1.18 (1.1, 1.27)	8.61E-06
rs191228682	MOB1B	4	70926914	0.0043	G	2.03 (1.39, 2.96)	2.30E-04
rs555550574	SLC4A4	4	71535431	0.0016	T	2.99 (1.64, 5.42)	3.26E-04
rs540994912	ADAMTS3	4	72488073	0.0018	C	2.81 (1.61, 4.91)	2.80E-04
rs1431791817		4	73924398	0.0009	T	5.02 (2.28, 11.03)	5.90E-05
rs4359938	ART3	4	76051029	0.0577	G	1.28 (1.13, 1.45)	1.03E-04
rs200409447	ART3	4	76109337	0.0048	A	2.02 (1.41, 2.9)	1.31E-04
rs143673870	FRAS1	4	78430717	0.002	G	3.25 (1.91, 5.53)	1.40E-05
rs189702173	C4orf22	4	80380336	0.0028	G	2.33 (1.47, 3.7)	3.21E-04
rs551005806	LIN54	4	82942850	0.003	G	2.75 (1.77, 4.26)	6.09E-06
rs1419242974		4	83219183	0.0006	C	7.14 (2.55, 20)	1.85E-04
rs992411646	HPSE	4	83306650	0.0026	C	3.21 (2, 5.15)	1.39E-06
rs544702576	GPAT3	4	83581796	0.0012	G	4.29 (2.13, 8.66)	4.72E-05
rs71597304	LOC101928978	4	84250875	0.0042	T	2.36 (1.62, 3.43)	7.15E-06
rs1302702878		4	85202849	0.0004	T	12.57 (3.22, 49.11)	2.72E-04
rs181953760	ARHGAP24	4	85482756	0.0005	G	7.76 (2.52, 23.87)	3.49E-04
rs1489646585		4	96115045	0.001	G	4.06 (1.92, 8.57)	2.40E-04
rs145011820	LINC02267;STPG2-AS1	4	97153912	0.0015	A	3.7 (2.01, 6.83)	2.78E-05
rs191022266	FLJ20021;BANK1	4	101499866	0.0007	G	6.16 (2.57, 14.79)	4.73E-05
rs574757298	LEF1-AS1;RPL34-AS1	4	108444415	0.0011	A	3.77 (1.85, 7.67)	2.53E-04
rs148618084	RPL34-AS1	4	108540396	0.0073	T	1.77 (1.31, 2.4)	1.89E-04
rs140147930	ANK2	4	113165188	0.0088	G	1.76 (1.33, 2.32)	6.01E-05
rs117139259	ARSJ;UGT8	4	114021293	0.0179	C	0.6 (0.46, 0.78)	1.42E-04
rs780978847	LINC02263;LINC01378	4	117413237	0.0012	A	3.59 (1.81, 7.1)	2.53E-04
rs560479060	LINC02502;MAD2L1	4	120031769	0.0036	C	2.34 (1.56, 3.51)	3.89E-05
rs1467808941		4	125252574	0.0019	A	2.82 (1.62, 4.89)	2.33E-04
rs7677873	MIR2054;INTU	4	126386387	0.4372	T	0.88 (0.83, 0.93)	1.71E-05
rs138747835	MIR2054;INTU	4	126497230	0.0123	G	0.55 (0.4, 0.76)	2.76E-04
rs1242221594		4	129482175	0.013	A	0.55 (0.4, 0.75)	2.21E-04
rs796370882	LINC02479;SNHG27	4	130493310	0.0007	G	5.22 (2.11, 12.88)	3.37E-04
chr4:132808070_T_TA		4	132808070	0.0506	T	1.26 (1.11, 1.43)	3.08E-04
rs556713354	LINC02462;LINC02485	4	134609380	0.0041	C	2.08 (1.41, 3.07)	2.42E-04
rs114247487	LINC02462;LINC02485	4	134841541	0.0192	A	1.46 (1.21, 1.78)	1.21E-04
rs78808601	LINC02485;LINC00613	4	135434609	0.0043	T	2.08 (1.42, 3.04)	1.55E-04

rs536091798	LINC00499;LOC105377448	4	138446369	0.0008	T	4.71 (2.06, 10.79)	2.48E-04
rs371444839	LINC00499;LOC105377448	4	138809102	0.0014	T	3.57 (1.9, 6.71)	7.99E-05
rs148798921	ARHGAP10	4	147740027	0.0011	C	4.02 (1.96, 8.24)	1.51E-04
rs1206522078	LRBA	4	150415041	0.0012	A	3.94 (2.02, 7.68)	5.61E-05
rs1443620517	LRBA	4	150992114	0.0012	G	3.86 (1.98, 7.5)	6.89E-05
rs1251803452	FHIP1A	4	151466636	0.0012	T	3.88 (1.96, 7.7)	1.04E-04
rs1201671794	LOC105377488	4	151881515	0.0012	G	3.98 (2.05, 7.74)	4.68E-05
rs890393323	SFRP2;DCHS2	4	154204761	0.0069	G	1.83 (1.35, 2.49)	9.69E-05
rs1172727532		4	156505958	0.0007	C	5.95 (2.39, 14.83)	1.30E-04
rs1166554753		4	160777629	0.0015	CTTAT	3.58 (1.96, 6.53)	3.32E-05
rs373407829		4	162346417	0.0016	C	3.49 (1.93, 6.29)	3.27E-05
rs1330443460	MARCHF1	4	164148303	0.0007	A	5.68 (2.29, 14.1)	1.81E-04
rs187141186	TLL1;SPOCK3	4	166391395	0.0006	T	6.68 (2.47, 18.04)	1.78E-04
rs1415389057		4	166542919	0.0006	T	6.6 (2.47, 17.59)	1.62E-04
rs938996749	LINC01612;LINC02382	4	170716307	0.0005	A	8.92 (2.77, 28.72)	2.45E-04
rs181106829	GALNTL6	4	172623906	0.0184	A	1.71 (1.41, 2.08)	6.00E-08
rs75215134	AGA;LINC01098	4	177611677	0.0563	T	1.26 (1.12, 1.42)	1.55E-04
rs1199683261	WWC2	4	183113434	0.0011	T	3.69 (1.81, 7.51)	3.14E-04
rs118017877	CDKN2AIP;LOC389247	4	183487833	0.0412	T	1.31 (1.14, 1.51)	1.82E-04
rs1038512770	LINC02436	4	185060746	0.0042	C	2.16 (1.47, 3.18)	9.05E-05
rs186378751	SORBS2	4	185858247	0.0127	G	1.57 (1.23, 1.99)	2.30E-04
rs182554256	TLR3;FAM149A	4	186091388	0.0008	C	6.47 (2.77, 15.08)	1.54E-05
rs143829794	ZFP42	4	187999625	0.0006	T	[illegible]E-07 (3.43E-10, 1.08E	1.78E-04
rs138162834	ZFP42	4	188000748	0.0363	G	1.32 (1.13, 1.54)	3.46E-04
rs77560770	TRIML1;LINC01060	4	188322907	0.104	C	1.21 (1.1, 1.33)	4.73E-05
rs2376749	TRIML1;LINC01060	4	188366191	0.496	G	0.89 (0.84, 0.95)	1.52E-04
rs576107776	IRX1;LINC02114	5	3643130	0.0008	C	4.55 (2.03, 10.18)	2.27E-04
rs7708156	IRX1;LINC02114	5	4535608	0.0235	C	1.39 (1.16, 1.65)	3.25E-04
rs543110098	ICE1;LINC02145	5	5990609	0.0011	T	3.71 (1.83, 7.55)	2.94E-04
rs183722912	ADCY2	5	7773426	0.0011	G	4.08 (2, 8.3)	1.09E-04
rs117762086	MYO10	5	16843955	0.042	C	1.35 (1.18, 1.54)	1.58E-05
rs371650489	CDH12	5	22225357	0.0004	T	12.23 (3.2, 46.69)	2.50E-04
rs62361592	PDZD2	5	31863094	0.0412	G	1.29 (1.12, 1.49)	3.43E-04
rs1279972543	LOC124900955	5	32900033	0.0006	GC	7.3 (2.68, 19.88)	9.90E-05
rs1464472367	ADAMTS12	5	33578154	0.001	G	4.28 (2.04, 9)	1.24E-04
rs202166473	SLC1A3;NIPBL-AS1	5	36865763	0.0012	C	3.92 (2.01, 7.64)	6.09E-05

rs141493384	LIFR-AS1	5	38594931	0.0022	C	2.65 (1.58, 4.44)	2.13E-04
rs10076705	OSMR	5	38909626	0.06	C	1.24 (1.1, 1.4)	2.91E-04
rs199946315	MROH2B;C6	5	41083624	0.0048	TA	1.98 (1.38, 2.85)	2.18E-04
rs775870112	ZNF131	5	43122025	0.0012	CTCT	3.51 (1.8, 6.84)	2.36E-04
rs538153634	NNT	5	43619275	0.0014	C	3.57 (1.9, 6.71)	8.01E-05
rs1025402158	NNT;FGF10	5	44132813	0.0014	A	3.28 (1.77, 6.1)	1.68E-04
rs1329640571		5	50914290	0.0017	A	3.06 (1.74, 5.39)	1.10E-04
rs575042560	PARP8;LINC02106	5	50949564	0.0021	C	2.64 (1.55, 4.49)	3.35E-04
rs1243154186		5	51298918	0.0013	A	4.75 (2.44, 9.24)	4.46E-06
rs150646326	IL31RA;IL6ST	5	55925675	0.0088	G	1.68 (1.27, 2.22)	2.57E-04
rs144271395	ACTBL2;LINCR-0003	5	57505881	0.0029	A	2.34 (1.49, 3.7)	2.48E-04
rs201014707	LINC02225;LINC02101	5	58012546	0.0012	G	3.86 (1.93, 7.74)	1.41E-04
rs1182277496	PDE4D	5	59032853	0.0006	C	6.87 (2.46, 19.19)	2.33E-04
rs1219584028	ZSWIM6	5	61348632	0.0017	G	2.95 (1.65, 5.27)	2.62E-04
rs116436091	C5orf64-AS1;KIF2A	5	61743118	0.0154	C	1.53 (1.23, 1.89)	1.06E-04
rs146490773	CD180;LINC02242	5	67286598	0.0013	A	3.35 (1.76, 6.39)	2.29E-04
rs191101779	PIK3R1;LINC02198	5	68623137	0.0257	A	1.41 (1.19, 1.68)	9.61E-05
rs144695277	CARTPT;MAP1B	5	72001359	0.0013	T	3.37 (1.77, 6.43)	2.28E-04
rs140327845	PTCD2;ZNF366	5	72438658	0.0009	C	4.29 (1.97, 9.38)	2.56E-04
rs140727619	POC5;SV2C	5	75853285	0.1382	G	1.17 (1.07, 1.27)	2.69E-04
rs572088031	AP3B1	5	78218100	0.0008	TAAAG	4.78 (2.07, 11.07)	2.58E-04
rs376971306	XRCC4;VCAN	5	83386478	0.006	G	1.89 (1.36, 2.62)	1.45E-04
rs1190102926		5	83767429	0.0007	A	5.84 (2.35, 14.51)	1.47E-04
rs1352177196		5	86496720	0.0009	T	4.59 (2.14, 9.84)	8.95E-05
rs189396551	LINC02059;MIR4280	5	86840986	0.0061	T	1.88 (1.36, 2.6)	1.47E-04
rs760581453	MEF2C-AS1	5	88928048	0.0007	C	5.47 (2.18, 13.7)	2.89E-04
rs76858200	ADGRV1	5	90636052	0.0029	G	2.44 (1.55, 3.83)	1.17E-04
rs1319785289		5	99087191	0.0016	G	3.18 (1.77, 5.73)	1.13E-04
rs1226823348		5	99598269	0.0017	C	3.13 (1.77, 5.54)	8.93E-05
rs145925000	NUDT12;RAB9BP1	5	104687147	0.0009	T	4.37 (2.04, 9.34)	1.42E-04
rs1260595896	LOC105379110	5	105291624	0.0009	T	5.09 (2.31, 11.21)	5.49E-05
rs570675553	EFNA5	5	107524117	0.0012	T	3.94 (1.98, 7.83)	9.16E-05
rs149376014	FBXL17;LINC01023	5	108718671	0.0106	A	1.66 (1.28, 2.16)	1.51E-04
rs186713900	FER	5	109133903	0.0038	A	2.15 (1.43, 3.23)	2.17E-04
rs532272525	LINC01848;TMEM232	5	110312182	0.0007	T	5.8 (2.32, 14.5)	1.71E-04
rs190639193	EPB41L4A-AS2;LINC02200	5	112578974	0.0022	A	2.7 (1.61, 4.5)	1.49E-04
chr5:113974680_G_A		5	113974680	0.0005	G	10.42 (3.23, 33.6)	8.78E-05

rs149689014	TRIM36;PGGT1B	5	115201008	0.0072	T	1.77 (1.31, 2.4)	2.13E-04
rs779139	LINC02214;LINC00992	5	116924417	0.0878	G	1.23 (1.12, 1.36)	2.63E-05
rs79699383	LINC02214;LINC00992	5	117014042	0.0148	C	1.55 (1.24, 1.93)	1.11E-04
rs1360222654		5	117053181	0.0004	C	12.76 (3.21, 50.81)	3.03E-04
rs191964340	DTWD2	5	118873292	0.0027	G	2.6 (1.63, 4.14)	5.75E-05
rs1327203272	ADAMTS19	5	129506395	0.001	CTTTTTAAAAGTAG CTTTCTTG	3.84 (1.85, 7.96)	3.02E-04
rs182820055	P4HA2;PDLIM4	5	132230747	0.0036	G	2.25 (1.48, 3.41)	1.43E-04
rs540795778	IL4;KIF3A	5	132686178	0.0007	C	6.49 (2.54, 16.6)	9.61E-05
rs911074495	CAMLG;DDX46	5	134757423	0.0022	G	2.61 (1.55, 4.38)	2.89E-04
rs372399027	SIL1	5	138951178	0.0005	G	7.69 (2.57, 23.02)	2.65E-04
rs17100697	MIR5197;HMHB1	5	143713812	0.1676	C	1.17 (1.08, 1.27)	6.21E-05
rs577679420	STK32A	5	147366742	0.0027	C	2.68 (1.68, 4.26)	3.16E-05
rs549811989	JAKMIP2-AS1	5	147652343	0.0023	C	2.91 (1.77, 4.78)	2.61E-05
rs147792841	SPINK1;SCGB3A2	5	147843044	0.0249	C	0.67 (0.54, 0.84)	3.40E-04
rs34960718	AFAP1L1	5	149309520	0.1831	T	0.85 (0.78, 0.92)	1.16E-04
rs74719472	SYNPO	5	150607277	0.131	C	1.18 (1.09, 1.29)	9.38E-05
rs1220612480		5	155558629	0.0009	G	4.05 (1.9, 8.67)	3.07E-04
rs138371355	PWWP2A;FABP6	5	160120781	0.002	A	2.74 (1.61, 4.66)	2.15E-04
rs140157217	MAT2B;LINC02143	5	163964282	0.0287	A	1.37 (1.16, 1.61)	1.47E-04
rs146881621	MAT2B;LINC02143	5	164089003	0.0369	T	1.34 (1.16, 1.56)	9.90E-05
rs78100725	LINC01947;LOC10192790 8	5	166944087	0.0222	A	1.46 (1.22, 1.76)	5.42E-05
rs202160674	RARS	5	168516761	0.0073	T	1.78 (1.31, 2.41)	1.92E-04
rs140993960	DOCK2;FAM196B	5	169867610	0.0075	T	1.74 (1.28, 2.36)	3.49E-04
rs562390424	LCP2;LINC01366	5	170324913	0.001	C	4.49 (2.11, 9.55)	9.43E-05
rs970303522	HMP19;LINC01411	5	174282104	0.0028	T	2.47 (1.56, 3.9)	1.12E-04
rs192673108	LINC01411	5	174523530	0.0012	A	3.54 (1.81, 6.93)	2.29E-04
rs80350015	CPLX2;THOC3	5	175891984	0.0353	T	1.32 (1.14, 1.53)	2.24E-04
rs973087255	NSD1	5	177241248	0.0018	G	2.98 (1.65, 5.38)	2.95E-04
rs554615099	COL23A1	5	178491986	0.0009	C	5.3 (2.42, 11.58)	2.91E-05
rs903983	GRM6	5	178995825	0.1148	A	1.2 (1.1, 1.31)	6.09E-05
chr5:180554444_TCTT A_T		5	180554444	0.0009	TCTTA	4.4 (2, 9.67)	2.23E-04
rs917036796	FARS2;NRN1	6	5852651	0.0003	G	[illegible]4E-08 (1.05E-11, 5.85E[illegible]	3.23E-04
rs9502508	LY86;RREB1	6	6807140	0.0382	C	1.32 (1.13, 1.53)	3.24E-04
rs184908653	LY86;RREB1	6	6888389	0.0058	G	1.86 (1.33, 2.6)	2.58E-04
rs1443027551		6	10367740	0.0012	TC	4.48 (2.26, 8.88)	1.78E-05

rs532394949	LOC100506207;TFAP2A	6	10389668	0.0012	G	3.58 (1.8, 7.12)	2.84E-04
rs540579903	MIR5689HG;GCNT2	6	10481882	0.0024	A	2.56 (1.56, 4.19)	1.94E-04
rs76054173	GFOD1;SIRT5	6	13566910	0.0186	G	1.46 (1.2, 1.78)	1.80E-04
rs144824472	JARID2	6	15250029	0.0163	A	1.5 (1.21, 1.86)	2.01E-04
rs978482517	RBM24;CAP2	6	17302508	0.0027	A	2.43 (1.51, 3.9)	2.37E-04
rs62393843	CAP2	6	17525045	0.0018	C	3.14 (1.79, 5.51)	6.20E-05
rs553426217	MIR548A1;LOC101928519	6	18710971	0.0087	A	1.67 (1.27, 2.21)	2.91E-04
rs558757333	LOC105374960;LOC100506885	6	19481250	0.0088	T	1.73 (1.31, 2.29)	1.02E-04
rs9295565	LOC105374972;NRSN1	6	22742202	0.1238	A	1.18 (1.08, 1.29)	1.74E-04
rs117710282	C6orf62;GMNN	6	24743858	0.0443	T	0.71 (0.6, 0.84)	7.21E-05
rs185848342		6	31290825	0.0737	A	0.79 (0.69, 0.89)	2.38E-04
rs9264944	LOC112267902	6	31306842	0.379	A	1.17 (1.08, 1.26)	4.34E-05
rs3130475		6	31457001	0.0938	G	1.21 (1.09, 1.33)	2.44E-04
rs545902574	C2	6	31914826	0.0213	T	1.45 (1.19, 1.76)	1.60E-04
rs1298611974	ILRUN	6	34660367	0.0007	A	5.17 (2.14, 12.49)	2.62E-04
rs1157948307	ANKS1A	6	34946504	0.0007	C	5.15 (2.13, 12.43)	2.72E-04
rs1432083556	PPARD	6	35352845	0.0007	A	5.27 (2.17, 12.82)	2.46E-04
chr6:35611237_CTCT_C		6	35611237	0.0024	CTCT	2.84 (1.74, 4.62)	2.68E-05
rs55987213	FKBP5	6	35694255	0.0616	C	0.75 (0.66, 0.86)	5.36E-05
rs570956586	NCR2;LINC01276	6	41362515	0.0021	T	2.61 (1.55, 4.39)	3.07E-04
rs12527949	TFEB	6	41704581	0.1998	G	1.15 (1.07, 1.24)	8.63E-05
rs6910726	VEGFA;LINC01512	6	43822176	0.1979	A	1.17 (1.09, 1.25)	2.43E-05
rs191720745	LOC101926915	6	46279187	0.0037	C	2.25 (1.5, 3.37)	8.40E-05
rs1168344440	IL17F	6	52241025	0.0037	A	2.21 (1.47, 3.32)	1.40E-04
rs3823112	GCM1	6	53128827	0.0098	G	1.64 (1.26, 2.12)	1.96E-04
rs1333661192	COL21A1	6	56232773	0.0014	C	3.31 (1.75, 6.28)	2.45E-04
rs140197073	EYS	6	65237164	0.0123	C	1.57 (1.24, 1.99)	1.92E-04
rs534183056	EYS;SLC25A51P1	6	65752884	0.0131	G	1.54 (1.22, 1.94)	2.56E-04
rs1938055	SLC25A51P1;NONE	6	65820449	0.1242	T	1.17 (1.08, 1.28)	2.98E-04
rs145009062	SLC25A51P1;LOC102723883	6	67783697	0.0326	G	1.33 (1.14, 1.55)	2.19E-04
rs191539519	LOC101928516	6	74327837	0.0024	A	2.61 (1.62, 4.2)	7.71E-05
rs181412018	MEI4;IRAK1BP1	6	78704433	0.0075	T	0.39 (0.24, 0.63)	9.22E-05
rs146434843	RNY4;LINC01621	6	79748177	0.0215	C	0.64 (0.5, 0.81)	2.00E-04
rs6911929	MAP3K7;MIR4643	6	91410726	0.0004	G	11.74 (3.15, 43.67)	2.39E-04

rs57434066		6	98335448	0.0072	CTT	1.84 (1.36, 2.51)	9.60E-05
rs149893831	MIR2113;POU3F2	6	98615650	0.0112	T	0.49 (0.34, 0.7)	1.01E-04
rs202221033	MIR2113;POU3F2	6	98698472	0.0011	G	3.62 (1.8, 7.28)	3.03E-04
rs78936830	FBXL4;MIR548AI	6	99084705	0.0229	G	0.66 (0.53, 0.83)	2.66E-04
rs73759376	GRIK2;NONE	6	102145017	0.0095	G	1.65 (1.26, 2.15)	2.33E-04
rs775020161	MICAL1	6	109449982	0.0007	A	4.93 (2.08, 11.67)	2.89E-04
rs187923671	FIG4;GPR6	6	109936851	0.0036	T	2.37 (1.57, 3.57)	4.00E-05
rs1379501685	REV3L	6	111474237	0.0009	G	4.58 (2.13, 9.83)	9.69E-05
rs74831568	ROS1	6	117306438	0.0546	G	1.27 (1.13, 1.44)	9.92E-05
rs188784238	NUS1;SLC35F1	6	117879350	0.0059	T	1.87 (1.35, 2.59)	1.75E-04
rs184263010	SLC35F1	6	118268049	0.0044	G	2.28 (1.58, 3.31)	1.22E-05
rs182683319	CEP85L	6	118598626	0.0046	G	2.25 (1.56, 3.24)	1.34E-05
rs1331056765		6	121374750	0.0008	AT	4.87 (2.09, 11.35)	2.51E-04
rs188279116	TBC1D32;GJA1	6	121382614	0.0006	G	6.06 (2.3, 15.99)	2.69E-04
rs562356820	GJA1;HSF2	6	121831149	0.0015	G	3.18 (1.75, 5.78)	1.53E-04
rs183038971	HDDC2;LINC02523	6	125486225	0.0028	A	2.34 (1.47, 3.73)	3.33E-04
rs1260692276	LINC02523	6	125684705	0.002	A	2.9 (1.69, 4.97)	1.07E-04
rs190585672	RSPO3;RNF146	6	127233043	0.0009	G	4.51 (2.04, 10.01)	2.09E-04
rs1179113152	PTPRK	6	128160368	0.0007	T	5.33 (2.2, 12.88)	2.05E-04
rs117185735	ARHGAP18	6	129662223	0.0412	A	1.29 (1.13, 1.49)	2.72E-04
rs4895398	SGK1	6	134217886	0.0154	T	1.5 (1.21, 1.87)	2.46E-04
rs142534694	LINC00271;PDE7B	6	135839924	0.0098	G	1.66 (1.28, 2.17)	1.66E-04
rs142610554	BCLAF1;MAP7	6	136320425	0.0447	T	1.29 (1.13, 1.47)	2.06E-04
rs75779481	NHEG1	6	136983881	0.0255	G	0.66 (0.53, 0.82)	2.05E-04
rs144841611	GVQW2	6	138732859	0.0013	C	3.37 (1.75, 6.5)	2.90E-04
rs144431572	LINC01625;LOC100132735	6	139590039	0.0007	T	6.98 (2.82, 17.24)	2.57E-05
rs560044890	LINC01625;LOC100132735	6	139696913	0.0012	C	3.52 (1.78, 6.96)	3.04E-04
rs191552608	LINC01277	6	143001721	0.0035	C	2.17 (1.43, 3.3)	2.79E-04
rs1162818666	PHACTR2	6	143704076	0.001	G	4 (1.93, 8.32)	2.04E-04
rs369710186	GRM1	6	146261395	0.003	G	2.3 (1.48, 3.58)	2.13E-04
rs76472646	STXBP5-AS1	6	147064749	0.0081	C	1.71 (1.28, 2.29)	2.90E-04
rs374445803	SAMD5;SASH1	6	148098643	0.0012	G	3.68 (1.83, 7.41)	2.63E-04
rs1414541693	SASH1	6	148194771	0.0019	G	2.89 (1.66, 5.01)	1.70E-04
rs577157386		6	148561687	0.0046	G	2.11 (1.46, 3.06)	7.17E-05
rs146707202	UST-AS1	6	148960352	0.0013	G	4.36 (2.28, 8.36)	9.01E-06
rs1346430616	GINM1	6	149564773	0.0014	G	3.24 (1.71, 6.12)	3.07E-04

rs185784115	PPP1R14C;IYD	6	150273110	0.0013	G	3.97 (2.06, 7.67)	3.88E-05
rs191854949	MIR1273C;TIAM2	6	154862172	0.003	A	2.36 (1.51, 3.7)	1.79E-04
rs563210555	TFB1M;NOX3	6	155318937	0.0035	A	2.42 (1.61, 3.65)	2.32E-05
rs558621137	LOC105378068;MIR1202	6	155642116	0.004	G	2.08 (1.41, 3.07)	2.00E-04
rs532199177	LOC105378068;MIR1202	6	155944548	0.0041	C	2.49 (1.71, 3.63)	2.27E-06
rs186401201	MIR1202;SNORD28B	6	156050244	0.0031	T	2.45 (1.55, 3.87)	1.15E-04
rs146479289	MIR1202;SNORD28B	6	156220910	0.0123	C	1.57 (1.23, 1.99)	2.81E-04
rs190018955	SNORD28B;ARID1B	6	156562509	0.0017	C	2.98 (1.65, 5.39)	3.01E-04
rs139198064	MEAT6;C6orf118	6	164992254	0.1018	TG	0.82 (0.74, 0.91)	1.13E-04
rs1464218151	PDE10A	6	165696274	0.0004	C	11.39 (3, 43.19)	3.47E-04
rs139305991	RPS6KA2;RPS6KA2-AS1	6	166870866	0.0034	G	2.21 (1.45, 3.39)	2.42E-04
rs66822994	TCP10L2;UNC93A	6	167257619	0.1863	G	0.86 (0.79, 0.93)	2.16E-04
rs377675972	DACT2;SMOC2	6	168361571	0.0013	C	3.41 (1.78, 6.52)	2.12E-04
rs186363943	SMOC2	6	168518145	0.0057	A	0.33 (0.18, 0.59)	1.81E-04
rs111656694	WDR27	6	169636370	0.001	C	4.14 (1.97, 8.7)	1.83E-04
rs557146153	FAM120B;PSMB1	6	170529170	0.0007	T	5.49 (2.22, 13.57)	2.30E-04
rs537707540	PRKAR1B	7	664656	0.001	C	4.35 (2.09, 9.05)	8.72E-05
rs369507757	MAD1L1	7	2146280	0.0013	T	3.86 (1.99, 7.47)	6.40E-05
chr7:3574123_C_CT		7	3574123	0	C	E+07 (3.89E+03, 6.75E	2.44E-04
rs140442049	SDK1;FOXK1	7	4673004	0.0207	G	1.41 (1.17, 1.7)	3.28E-04
rs191604312	FAM220A	7	6333062	0.0012	C	3.44 (1.76, 6.73)	3.08E-04
rs535767313	CCZ1B;MIR3683	7	6876862	0.0011	C	4.08 (2.01, 8.29)	9.76E-05
rs866405722	UMAD1	7	7802379	0.0009	A	4.63 (2.1, 10.21)	1.45E-04
rs939888605	ARL4A;ETV1	7	12776341	0.0005	C	8.5 (2.9, 24.86)	9.41E-05
rs141304236	ARL4A;ETV1	7	13867235	0.0035	C	2.16 (1.42, 3.28)	2.89E-04
rs745983585	ETV1	7	13981518	0.0174	TAC	1.55 (1.27, 1.9)	2.05E-05
rs111898693	DGKB	7	14170003	0.0066	A	0.34 (0.2, 0.58)	8.06E-05
rs184488875	DGKB	7	14275808	0.0129	G	1.55 (1.22, 1.97)	2.75E-04
rs909333686	PRPS1L1;HDAC9	7	18055511	0.0031	C	2.27 (1.46, 3.52)	2.67E-04
rs952891253	HDAC9	7	18576504	0.0026	A	2.59 (1.62, 4.17)	8.07E-05
rs117504787	FERD3L	7	19144247	0.0199	C	1.49 (1.23, 1.81)	5.63E-05
rs139577363	FERD3L;TWISTNB	7	19601696	0.0441	T	1.29 (1.13, 1.48)	1.44E-04
rs1347071216		7	20611235	0.0013	T	3.78 (1.97, 7.25)	6.52E-05
rs1280403258		7	23856647	0.0017	CTCTT	2.97 (1.66, 5.31)	2.34E-04
rs1393525768		7	25513003	0.0014	G	3.32 (1.77, 6.25)	1.89E-04
rs1360279465		7	25927535	0.0012	C	3.74 (1.88, 7.45)	1.76E-04
rs372033392	CREB5	7	28499260	0.0012	A	4.37 (2.24, 8.54)	1.54E-05
rs56192163	LOC646762	7	29668978	0.071	CTCTT	1.24 (1.11, 1.38)	1.24E-04

rs775195892	DPY19L2P3;WIPF3	7	29732130	0.0011	C	3.73 (1.86, 7.48)	2.17E-04
rs1478845417	PDE1C	7	31715336	0.0008	T	4.75 (2.09, 10.8)	1.95E-04
rs140205715	BBS9	7	33637514	0.0051	A	2.05 (1.44, 2.91)	6.34E-05
rs569644346	SEPT7;LOC101928618	7	35998913	0.0014	G	3.39 (1.8, 6.39)	1.55E-04
rs35756248	PURB;MYO1G	7	44946247	0.3946	A	0.89 (0.84, 0.95)	2.83E-04
rs566367314	IGFBP3;LOC730338	7	46264845	0.0042	G	2.21 (1.51, 3.24)	4.17E-05
rs189840784	IGFBP3;LOC730338	7	46650717	0.0028	C	2.48 (1.58, 3.91)	8.31E-05
rs538215701	LOC730338;TNS3	7	46946107	0.0012	G	3.93 (2.01, 7.66)	5.94E-05
rs1480140628		7	53334056	0.0013	T	3.58 (1.88, 6.81)	1.06E-04
rs536034766	LINC01446	7	53706204	0.0007	T	6.71 (2.76, 16.32)	2.65E-05
rs147116650	LINC01446	7	53806322	0.0157	C	1.57 (1.27, 1.94)	2.87E-05
rs1389311474		7	61921937	0.0007	C	5.06 (2.11, 12.12)	2.77E-04
rs1452803451		7	62426178	0.0007	TA	5.14 (2.14, 12.34)	2.53E-04
rs1332109537	ZNF273	7	64898478	0.0006	C	5.75 (2.26, 14.6)	2.35E-04
rs148989345	ZNF92;LOC441242	7	65609007	0.0292	G	1.34 (1.14, 1.57)	3.21E-04
rs1453778314	LINC03006	7	65749921	0.0006	T	6.85 (2.59, 18.16)	1.08E-04
rs1420597796		7	66366969	0.0006	A	7.17 (2.69, 19.11)	8.22E-05
rs1195477313	GTF2IRD1P1	7	66812393	0.0006	T	7.07 (2.66, 18.82)	8.90E-05
rs186073204	AUTS2	7	70596703	0.0012	C	3.84 (1.92, 7.69)	1.43E-04
rs1351163262		7	71098636	0.0006	G	6.38 (2.4, 16.93)	2.00E-04
chr7:81003117_T_TA		7	81003117	0.4301	T	0.89 (0.83, 0.94)	1.24E-04
rs191963875	SEMA3C;LOC105369146	7	81493123	0.0089	T	1.7 (1.29, 2.23)	1.62E-04
rs76698833	LOC100128317	7	81681625	0.0235	A	1.41 (1.17, 1.68)	2.08E-04
rs370821991	PCLO;SEMA3E	7	83322039	0.0017	CTG	2.94 (1.66, 5.21)	2.28E-04
rs75504046	SEMA3E;SEMA3A	7	83704762	0.2918	G	0.88 (0.82, 0.94)	1.07E-04
rs76796674	LINC00972;GRM3	7	86626016	0.1237	G	0.82 (0.75, 0.9)	4.22E-05
chr7:88304702_C_CA		7	88304702	0.0101	C	0.49 (0.34, 0.72)	2.49E-04
rs142722832	VPS50	7	93359545	0.0052	T	1.95 (1.38, 2.77)	1.83E-04
rs371119895	SGCE	7	94623856	0.0013	A	3.87 (2.05, 7.31)	3.15E-05
rs1050417765	PPP1R9A	7	94999002	0.001	C	3.74 (1.84, 7.62)	2.75E-04
rs188555667	ASNS;MIR5692A1	7	97887017	0.0069	T	1.84 (1.34, 2.53)	1.80E-04
rs560721567	BAIAP2L1;NPTX2	7	98508285	0.0029	G	2.6 (1.66, 4.07)	3.10E-05
rs5015756	ZCWPW1	7	100415834	0.2697	T	1.14 (1.07, 1.22)	1.30E-04
chr7:106257144_C_CA		7	106257144	0.0012	C	4.37 (2.13, 8.95)	5.60E-05
rs188311578	EIF3IP1;IMMP2L	7	110060424	0.0117	C	1.66 (1.31, 2.12)	3.81E-05
rs12670030	EIF3IP1;IMMP2L	7	110124779	0.2547	C	1.14 (1.07, 1.22)	1.04E-04
rs572418796	EIF3IP1;IMMP2L	7	110301468	0.0016	C	3.22 (1.79, 5.8)	9.26E-05
rs189595323	EIF3IP1;IMMP2L	7	110597486	0.002	T	3.14 (1.84, 5.34)	2.62E-05

rs377512161	IMMP2L	7	110848267	0.0008	C	5.37 (2.34, 12.34)	7.46E-05
rs191965352	IMMP2L	7	111417807	0.0017	A	2.98 (1.66, 5.34)	2.38E-04
rs190224826	IMMP2L;DOCK4	7	111707575	0.0016	A	3.38 (1.86, 6.14)	6.45E-05
rs568274411	DOCK4	7	111937323	0.0018	ACT	3.41 (1.96, 5.92)	1.40E-05
rs1444035190		7	116353994	0.0012	C	3.63 (1.85, 7.11)	1.75E-04
rs745937304	ST7;WNT2	7	117236795	0.0019	C	2.74 (1.58, 4.76)	3.46E-04
rs2041528	CTTNBP2;LSM8	7	118146309	0.4806	A	1.13 (1.07, 1.2)	4.47E-05
rs1523811	ANKRD7;LINC02476	7	118592886	0.2879	T	0.88 (0.83, 0.94)	2.81E-04
rs79475236	TSPAN12;ING3	7	120891610	0.0101	C	1.65 (1.28, 2.14)	1.40E-04
rs75789086	LOC101928211;GPR37	7	124427668	0.0207	T	1.45 (1.2, 1.76)	9.57E-05
rs17394006	LOC101928211;GPR37	7	124533735	0.1404	T	1.17 (1.07, 1.27)	3.42E-04
rs184709266	POT1	7	124853623	0.001	C	4.28 (2.01, 9.08)	1.55E-04
rs547356815	LOC101928283	7	125219731	0.001	A	3.87 (1.85, 8.13)	3.46E-04
rs368518397	TPI1P2;LOC407835	7	129084543	0.0024	G	2.75 (1.67, 4.53)	7.40E-05
rs1375049099		7	130054850	0.0015	TC	3.15 (1.74, 5.71)	1.55E-04
rs1025573742	LINC-PINT	7	130879306	0.0009	G	4.94 (2.22, 11)	9.03E-05
rs79491224	PLXNA4	7	132223128	0.0884	G	1.22 (1.1, 1.35)	1.93E-04
rs118048709	LOC105375512;EXOC4	7	133134220	0.0125	G	1.56 (1.23, 1.97)	2.60E-04
rs1330151742	LOC107984123	7	135260030	0.0016	A	2.98 (1.66, 5.32)	2.36E-04
rs2709987	MIR4468;TRIM24	7	138180087	0.319	G	0.88 (0.83, 0.94)	1.23E-04
rs149611499	HIPK2	7	139657269	0.0025	C	2.61 (1.62, 4.21)	8.61E-05
rs934275988	TMEM178B;AGK	7	141491510	0.0008	T	4.8 (2.07, 11.14)	2.52E-04
rs181358543	WEE2-AS1	7	141703462	0.005	A	1.93 (1.36, 2.75)	2.54E-04
rs779999204	TAS2R4	7	141778986	0.0008	A	4.7 (2.1, 10.51)	1.61E-04
rs370359601	CNTNAP2	7	146768996	0.0115	A	1.65 (1.28, 2.11)	9.23E-05
rs183860837	CNTNAP2	7	147697991	0.0017	C	3.14 (1.77, 5.57)	9.23E-05
rs538023647	CNTNAP2	7	147703104	0.0016	C	3.25 (1.79, 5.9)	1.01E-04
rs140399527	PRKAG2	7	151569679	0.0083	T	1.76 (1.32, 2.35)	1.20E-04
rs969252864	PRKAG2	7	151866599	0.0038	C	2.13 (1.42, 3.19)	2.58E-04
rs190789798	LINC01287;DPP6	7	153505072	0.0045	G	1.98 (1.37, 2.86)	2.79E-04
rs556280744	LINC01287;DPP6	7	153830556	0.0017	GAAGAAT	2.98 (1.67, 5.34)	2.32E-04
rs532492093	PTPRN2	7	157585503	0.0017	C	2.88 (1.62, 5.11)	3.02E-04
rs75318724	MYOM2	8	2085314	0.0313	G	1.38 (1.18, 1.61)	6.25E-05
rs570375004	CSMD1	8	3507041	0.0018	C	2.94 (1.67, 5.16)	1.85E-04
rs139731757	CSMD1	8	3547587	0.0036	T	2.16 (1.44, 3.25)	2.15E-04
rs543656548	CSMD1	8	3738464	0.008	T	2.03 (1.53, 2.68)	7.31E-07
rs911915720	CSMD1	8	4271172	0.001	A	4.17 (2.03, 8.56)	9.72E-05
rs147175476	CSMD1	8	4944984	0.0167	C	1.51 (1.21, 1.88)	2.17E-04

rs1194200857		8	5170206	0.0008	G	4.66 (2.02, 10.71)	2.94E-04
rs143152837	CSMD1;LOC100287015	8	5614932	0.0107	C	1.65 (1.28, 2.13)	1.13E-04
rs10098198	CSMD1;LOC100287015	8	5728664	0.0488	C	1.3 (1.15, 1.48)	4.77E-05
rs2243755	LOC100287015	8	6406418	0.4472	C	1.14 (1.08, 1.21)	1.03E-05
rs556657962	MCPH1	8	6429220	0.0065	CTT	2.09 (1.53, 2.85)	3.07E-06
rs1273067396	ANGPT2	8	6537867	0.001	T	4.38 (2.12, 9.09)	7.07E-05
rs7825703	MCPH1	8	6589625	0.041	C	0.74 (0.63, 0.87)	3.49E-04
rs10089682	AGPAT5;XKR5	8	6773572	0.0529	A	1.27 (1.12, 1.43)	1.83E-04
rs537278257	FAM86B3P;PRAG1	8	8300289	0.0017	G	3.04 (1.73, 5.36)	1.18E-04
rs187123147	ERI1	8	9010366	0.001	A	3.95 (1.9, 8.21)	2.40E-04
rs145738179	MSRA	8	10147580	0.0038	G	2.42 (1.63, 3.59)	1.27E-05
rs1363820124	SGCZ	8	14793799	0.0019	G	2.86 (1.67, 4.93)	1.42E-04
rs544931033	SGCZ	8	15134323	0.0027	T	2.4 (1.5, 3.84)	2.66E-04
rs1010759445	MTMR7;SLC7A2	8	17478478	0.0017	G	3.12 (1.76, 5.55)	1.07E-04
rs17575974	SNORD3F;LOC102467222	8	20703586	0.0048	A	2.23 (1.55, 3.2)	1.39E-05
rs143401439	CCAR2	8	22615258	0.0078	T	1.77 (1.33, 2.37)	1.15E-04
rs556927940	LOC101929294;NEFM	8	24659908	0.0009	G	4.4 (1.98, 9.76)	2.76E-04
rs1157755723		8	29403633	0.0013	T	4.1 (2.11, 7.96)	3.02E-05
rs28473264	MIR3148;SARAF	8	30021354	0.3529	T	0.89 (0.84, 0.95)	2.93E-04
rs540006541	DCTN6;RBPMS-AS1	8	30320109	0.0007	C	6.06 (2.39, 15.4)	1.51E-04
rs146337942	UNC5D;KCNU1	8	36761748	0.0054	G	1.9 (1.34, 2.69)	2.84E-04
rs182041160	TACC1;PLEKHA2	8	38854541	0.0023	T	3.04 (1.85, 5)	1.14E-05
rs190329945	ADAM18;ADAM2	8	39731830	0.0019	A	3.11 (1.81, 5.33)	3.80E-05
rs10092753	UBE2V2;LOC101929268	8	48213826	0.0207	A	0.63 (0.5, 0.8)	1.82E-04
rs551298539	LOC101929268	8	48600889	0.0024	G	2.49 (1.53, 4.08)	2.67E-04
rs147388862	LOC101929268;EFCAB1	8	48706222	0.0127	A	1.55 (1.23, 1.96)	2.47E-04
rs375486567	LOC100507464;SNTG1	8	49688534	0.0051	G	0.3 (0.16, 0.57)	2.35E-04
rs1346558801	SNTG1	8	50381613	0.0011	ATTAG	3.81 (1.86, 7.83)	2.67E-04
rs184307507	RB1CC1;NPBWR1	8	52820627	0.0116	A	1.59 (1.24, 2.04)	2.56E-04
chr8:54557859_A_AAATG		8	54557859	0.0027	A	2.38 (1.49, 3.8)	3.05E-04
rs145731921	TOX;CA8	8	59523797	0.0008	T	4.71 (2.1, 10.61)	1.77E-04
rs536643854	MIR4470;NKAIN3	8	62187945	0.0015	G	3.26 (1.79, 5.94)	1.12E-04
rs1323828171	C8orf34	8	68568593	0.0005	A	9.66 (3.04, 30.66)	1.19E-04
rs146539350	LINC01592;LINC01603	8	69134198	0.0225	C	1.39 (1.16, 1.67)	3.04E-04
rs1428054129	EYA1	8	71261802	0.0007	C	6.73 (2.65, 17.08)	6.04E-05
rs140928471	MIR2052HG	8	74634429	0.0425	G	1.31 (1.15, 1.5)	8.42E-05
rs60573028	ZFHX4-AS1	8	76639641	0.0206	C	0.65 (0.51, 0.82)	3.48E-04

rs151076392	LOC102724874;PKIA	8	77863834	0.039	G	1.38 (1.19, 1.59)	1.23E-05
rs1164821650	WWP1	8	86432754	0.0029	C	2.34 (1.48, 3.7)	2.97E-04
rs965719409	SLC26A7;RUNX1T1	8	91540752	0.0013	A	3.8 (1.98, 7.28)	5.99E-05
rs550832462	MIR7641-2;LOC102724710	8	92264842	0.0016	G	3.19 (1.8, 5.68)	7.76E-05
rs1342940436		8	95892614	0	A	E+07 (3.48E+03, 8.42E	1.23E-04
rs556605860	KCNV1;LINC01608	8	110527184	0.0013	G	3.38 (1.74, 6.54)	3.14E-04
rs909011353	CSMD3;TRPS1	8	115070449	0.0013	A	3.57 (1.87, 6.82)	1.20E-04
rs149343170	RAD21-AS1;AARD	8	116890626	0.0008	A	6.16 (2.68, 14.15)	1.80E-05
rs562849360	MED30;EXT1	8	117541710	0.0042	C	2.1 (1.44, 3.08)	1.34E-04
rs1469670296		8	121303930	0.0008	G	7.88 (3.33, 18.64)	2.58E-06
rs75897038	LINC01151;ZHX2	8	122754853	0.0021	C	2.84 (1.69, 4.79)	8.90E-05
rs1412441056	FER1L6-AS1	8	124021271	0.0001	T	LE-15 (6.09E-24, 1.66E	3.48E-04
rs556283334	FER1L6	8	124044092	0.0029	C	2.84 (1.83, 4.42)	3.73E-06
rs371385510	FER1L6-AS2	8	124064046	0.0017	C	2.89 (1.62, 5.14)	3.06E-04
rs145553201	LOC101927588	8	124209817	0.0073	C	1.79 (1.32, 2.41)	1.46E-04
rs11779830	TRIB1;LINC00861	8	125784156	0.098	T	0.82 (0.74, 0.91)	2.49E-04
rs560324610	FAM49B;MIR5194	8	129954521	0.0013	C	3.95 (2.04, 7.63)	4.44E-05
rs1481360064	ASAP1	8	130338573	0.0014	T	3.87 (2.06, 7.29)	2.69E-05
rs1160450818		8	131836915	0.0017	GT	2.94 (1.66, 5.2)	2.12E-04
rs144351106	ADCY8;EFR3A	8	131843367	0.0098	G	1.68 (1.29, 2.19)	1.06E-04
rs60142793	LRRC6	8	132587757	0.1216	G	1.17 (1.08, 1.28)	2.92E-04
rs137859262	LOC105375773	8	133677924	0.022	T	0.65 (0.52, 0.82)	3.29E-04
rs143728291	LOC101927845;LINC01591	8	135042509	0.0011	G	4.09 (1.99, 8.38)	1.24E-04
rs560953386	KHDRBS3;LINC02055	8	135713553	0.0028	G	2.31 (1.46, 3.66)	3.47E-04
rs956533903	KHDRBS3;LINC02055	8	136091937	0.0031	G	2.29 (1.48, 3.54)	2.03E-04
rs11996402	LINC02055;LOC101927915	8	137382753	0.0112	C	1.6 (1.25, 2.06)	1.98E-04
chr8:137907845_CTTGTTT_C		8	137907845	0.0012	CTTGTTT	3.98 (1.97, 8.03)	1.20E-04
rs1017282637	FAM135B	8	138129601	0.0013	C	3.18 (1.69, 5.98)	3.30E-04
rs73434810	FAM135B;COL22A1	8	138536536	0.1392	T	0.84 (0.77, 0.92)	1.41E-04
rs533830109	TRAPPC9	8	139923615	0.0008	G	5.23 (2.26, 12.09)	1.08E-04
rs567022304	MROH5;MIR1302-7	8	141577819	0.0018	C	2.82 (1.61, 4.93)	2.83E-04
rs1401856384		8	142086493	0.0026	A	2.44 (1.51, 3.94)	2.60E-04
rs1254683752	LOC105375951	9	1962697	0.0006	G	6.28 (2.43, 16.23)	1.51E-04
rs925637993	KDM4C	9	6840265	0.0017	G	3.17 (1.79, 5.64)	8.15E-05

rs7867126	TMEM261;PTPRD	9	8098188	0.1238	A	1.17 (1.08, 1.28)	3.17E-04
rs980641920	PTPRD-AS2;TYRP1	9	12236148	0.0004	G	10.75 (2.97, 38.92)	2.99E-04
rs545805788	ZDHHC21	9	14673527	0.0014	G	3.52 (1.87, 6.62)	9.28E-05
rs921491503	TTC39B;SNAPC3	9	15339695	0.0012	G	4.07 (2.08, 7.98)	4.26E-05
rs1025018588	CCDC171	9	15882460	0.0013	C	4.22 (2.18, 8.15)	1.81E-05
rs903428775	C9orf92	9	16249734	0.0011	G	3.88 (1.89, 7.95)	2.13E-04
rs376590698	IZUMO3;TUSC1	9	24561214	0.0019	C	2.77 (1.6, 4.79)	2.87E-04
rs778748033		9	24673659	0.0018	C	3.19 (1.82, 5.61)	5.40E-05
rs371839101	TUSC1;LINC01241	9	25733217	0.0018	G	2.9 (1.64, 5.14)	2.67E-04
rs185876644	SPATA31A5;LINC01410	9	43030668	0.0008	G	5.2 (2.24, 12.03)	1.19E-04
rs1418359371		9	61717903	0.0007	C	5.17 (2.22, 12.06)	1.43E-04
rs140281546	PGM5;TMEM252	9	68532309	0.0014	G	3.54 (1.86, 6.71)	1.09E-04
rs117201928	PIP5K1B	9	68805411	0.0323	A	1.33 (1.14, 1.56)	3.02E-04
rs143186779	APBA1;PTAR1	9	69687493	0.0012	A	3.93 (1.91, 8.06)	1.93E-04
rs1402118430	LOC101927281	9	73338023	0.0011	A	3.78 (1.85, 7.7)	2.56E-04
rs189516390	GNAQ	9	77718620	0.0053	T	1.96 (1.38, 2.78)	1.72E-04
rs953372919	LOC101927450;TLE4	9	79344288	0.0008	C	4.96 (2.1, 11.7)	2.54E-04
rs77866420	LINC01507;TLE1	9	80384470	0.0849	C	1.23 (1.11, 1.36)	6.46E-05
rs879537771	SPATA31D1;RASEF	9	82326770	0.0012	A	3.57 (1.82, 6.98)	2.04E-04
rs146265782	SLC28A3;NTRK2	9	84372935	0.0068	T	1.8 (1.32, 2.45)	2.10E-04
rs148380748	NAA35	9	85963364	0.0039	G	2.14 (1.43, 3.21)	2.35E-04
rs187167280	GOLM1	9	86056547	0.0014	C	3.64 (1.93, 6.89)	6.87E-05
rs557521003	ZCCHC6;GAS1	9	86699009	0.0021	G	2.6 (1.56, 4.35)	2.60E-04
rs34326837	SHC3;CKS2	9	89215263	0.009	C	1.64 (1.26, 2.16)	3.07E-04
rs148270806	LOC101927847;MIR4290HG	9	89991602	0.0257	C	0.64 (0.52, 0.79)	3.57E-05
rs138649015	LINC01501	9	90561051	0.0013	C	4.15 (2.12, 8.14)	3.42E-05
rs117728472	NFIL3;MIR3910-1	9	91494984	0.0376	T	0.72 (0.61, 0.86)	3.25E-04
chr9:91666097_A_AT		9	91666097	0.0019	A	2.93 (1.69, 5.06)	1.21E-04
rs574844830	FANCC	9	95207058	0.0022	T	2.9 (1.74, 4.85)	4.61E-05
rs57861943	PTCH1;LINC00476	9	95567069	0.1486	G	1.16 (1.07, 1.26)	3.41E-04
rs1341217287	ANKS6	9	98747372	0.0052	C	0.32 (0.17, 0.59)	2.81E-04
rs185982737	SEC61B;NAMA	9	99275914	0.0045	T	2.03 (1.38, 2.97)	3.02E-04
rs78023639	CAVIN4;PLPPR1	9	100650729	0.0119	T	1.62 (1.27, 2.07)	1.07E-04
rs535507376	ABCA1	9	104793117	0.002	G	2.72 (1.6, 4.61)	2.02E-04
rs7042562	TLR4;BRINP1	9	118703621	0.2947	A	1.16 (1.08, 1.23)	8.27E-06
rs185397685	BRINP1	9	119169300	0.0014	G	3.55 (1.88, 6.7)	9.06E-05
rs7849478	DAB2IP;TTLL11	9	121814542	0.3566	G	0.89 (0.83, 0.95)	3.05E-04

rs4837891	DAB2IP;TTLL11	9	121817761	0.2264	G	1.15 (1.07, 1.24)	1.00E-04
rs10819522	LINC01503;LINC00963	9	129436489	0.3307	T	1.13 (1.06, 1.21)	9.17E-05
rs79760079	ABL1	9	130753896	0.0272	T	1.36 (1.15, 1.6)	2.69E-04
rs200483322	GTF3C4;AK8	9	132704297	0.0064	T	1.85 (1.34, 2.55)	1.68E-04
rs183608002	AK8	9	132824130	0.0046	C	0.26 (0.13, 0.53)	2.24E-04
rs117405640	GFI1B;SNORD141A	9	133015880	0.0192	A	0.62 (0.48, 0.8)	2.08E-04
rs602060	RALGDS	9	133125058	0.325	A	0.87 (0.81, 0.93)	2.28E-05
rs183977740	RNU6ATAC;LINC02247	9	134212208	0.02	G	0.62 (0.49, 0.79)	1.35E-04
rs35084901	COL5A1	9	134787346	0.1474	T	0.85 (0.78, 0.93)	2.71E-04
rs143210859	FCN1;OLFM1	9	135027523	0.0082	AT	1.9 (1.44, 2.52)	7.86E-06
rs772604717	OLFM1	9	135118830	0.0624	GCCGGGTCTTTGG AGTGCTCA	0.77 (0.68, 0.89)	1.79E-04
rs867260809	ABCA2	9	137007436	0.0008	C	5.19 (2.18, 12.34)	1.94E-04
chr10:631561_G_GT		10	631561	0.0006	G	7.54 (2.78, 20.47)	7.34E-05
rs148112766	MIR6078;LOC101927964	10	4027973	0.0013	C	3.46 (1.8, 6.64)	1.89E-04
rs375810989	LINC00703;LINC00704	10	4465106	0.0016	A	3.04 (1.69, 5.46)	2.03E-04
rs1481871609	SFMBT2	10	7361038	0.0009	T	4.09 (1.89, 8.85)	3.46E-04
rs115752334	CCDC3	10	12948570	0.0063	G	1.92 (1.39, 2.64)	7.38E-05
rs1003740796	FRMD4A	10	14016869	0.0016	G	3.17 (1.76, 5.7)	1.19E-04
rs1400424561	FAM171A1	10	15224001	0.0006	T	6.65 (2.58, 17.17)	9.03E-05
rs965411901	ITGA8	10	15535357	0.0006	C	6 (2.26, 15.93)	3.25E-04
rs185069204	ITGA8	10	15556115	0.0056	C	1.91 (1.36, 2.69)	1.92E-04
rs955684955	MALRD1	10	19311714	0.0017	A	3 (1.68, 5.34)	1.98E-04
rs141105355	MALRD1	10	19587330	0.0039	T	2.16 (1.45, 3.19)	1.29E-04
rs973812768	PLXDC2;MIR4675	10	20477351	0.001	G	4.21 (2, 8.88)	1.57E-04
rs1528182	NEBL	10	20828578	0.001	A	4.18 (1.97, 8.87)	1.94E-04
rs139216372	ARMC3;MSRB2	10	23044107	0.0173	A	0.59 (0.45, 0.77)	1.11E-04
rs16924739	KIAA1217	10	24423935	0.0665	T	1.26 (1.13, 1.41)	4.47E-05
rs548339028	MPP7;LOC105376468	10	28359964	0.0051	C	1.93 (1.36, 2.76)	2.69E-04
rs1318136316	ZEB1	10	31435622	0.0006	A	7.43 (2.69, 20.57)	1.12E-04
rs1401157072	KIF5B	10	32048333	0.0007	AAAC	7.59 (3.03, 19.01)	1.52E-05
rs149170073	ITGB1;NRP1	10	33032592	0.001	C	4.02 (1.91, 8.46)	2.47E-04
rs956966875	PARD3	10	34207215	0.0063	TA	1.81 (1.31, 2.49)	3.18E-04
rs184010048	BMS1;LOC101929445	10	42836501	0.0191	C	0.61 (0.47, 0.79)	1.45E-04
rs183839907	ZNF487	10	43476582	0.0007	G	5.19 (2.14, 12.54)	2.59E-04
rs563279186	LINC01468;MBL2	10	52600044	0.0007	G	5.23 (2.15, 12.71)	2.59E-04
rs909071577	MBL2;PCDH15	10	53504683	0.0003	G	17.42 (3.67, 82.8)	3.27E-04
rs930738318	MIR3924;IPMK	10	57907794	0.0015	G	3.23 (1.75, 5.99)	1.88E-04

rs1223859931		10	58181200	0.0017	G	3.22 (1.81, 5.71)	6.46E-05
rs1045027593	TFAM;BICC1	10	58468791	0.0027	A	2.5 (1.57, 3.98)	1.16E-04
rs1432692493	ANK3	10	60444826	0.0016	C	3.05 (1.7, 5.45)	1.73E-04
rs2619401	REEP3;ANXA2P3	10	63810214	0.184	G	0.86 (0.79, 0.93)	3.13E-04
rs1490916630	TET1	10	68591001	0.0014	A	3.36 (1.78, 6.38)	2.00E-04
rs34895643		10	70659300	0.0012	AT	3.47 (1.77, 6.78)	2.76E-04
rs763064557	PCBD1;LOC105378349	10	70911239	0.0012	G	3.69 (1.84, 7.4)	2.27E-04
rs184388166	PCBD1;LOC105378349	10	70924116	0.002	G	2.85 (1.67, 4.87)	1.23E-04
rs191585688	SLC29A3	10	71358352	0.0015	C	3.19 (1.72, 5.93)	2.32E-04
rs189602821	CDH23	10	71698648	0.0004	C	11.45 (3.01, 43.55)	3.49E-04
rs1009347982		10	72238063	0.0013	C	3.33 (1.75, 6.34)	2.58E-04
rs147415995	MICU1	10	72378321	0.0008	G	5.79 (2.48, 13.52)	4.95E-05
rs369643328	MICU1;MCU	10	72631771	0.0008	G	4.93 (2.1, 11.61)	2.59E-04
rs1295370236	MCU	10	72726420	0.0014	G	3.22 (1.7, 6.09)	3.26E-04
rs201775756	P4HA1	10	73095083	0.0007	T	5.33 (2.25, 12.63)	1.47E-04
rs536450904	PPP3CB	10	73439597	0.0007	T	5.05 (2.09, 12.19)	3.19E-04
rs3812626	VCL	10	74047683	0.0014	T	3.29 (1.75, 6.16)	2.05E-04
rs181644593	LINC01520;GRID1-AS1	10	85557460	0.003	T	2.61 (1.69, 4.04)	1.67E-05
rs1392654293	RNLS	10	88353167	0.0005	A	9.03 (2.8, 29.08)	2.26E-04
rs537702039	LOC101926942	10	90530638	0.0005	C	9.21 (2.76, 30.75)	3.09E-04
rs149811183	MYOF	10	93417287	0.0018	C	2.85 (1.61, 5.03)	3.04E-04
rs367661610	C10orf76	10	102020618	0.0024	G	2.49 (1.54, 4.03)	2.10E-04
rs149835841	CFAP58	10	104386599	0.005	C	1.94 (1.36, 2.76)	2.61E-04
rs191678686	LOC101927523;SORCS3	10	104591939	0.0011	C	3.71 (1.83, 7.53)	2.81E-04
rs188372676	LOC101927523;SORCS3	10	104631589	0.001	A	4.18 (2.02, 8.66)	1.15E-04
rs146503201	LOC105378470;SORCS1	10	106561221	0.0096	A	1.7 (1.3, 2.22)	1.02E-04
rs2152676	SORCS1	10	106881621	0.0559	C	1.25 (1.11, 1.41)	2.27E-04
rs1185602672	LINC01435	10	107971282	0.0008	T	5.44 (2.38, 12.42)	5.94E-05
rs772118689	LINC01435;XPNPEP1	10	108103203	0.004	T	2.28 (1.54, 3.37)	3.81E-05
rs11195397	SHOC2	10	110990871	0.4752	C	0.9 (0.85, 0.95)	2.94E-04
rs192257220	ADRA2A;GPAM	10	111981129	0.0042	G	2.11 (1.45, 3.09)	1.10E-04
rs369408491	ADRA2A;GPAM	10	112011026	0.0005	G	9.35 (2.86, 30.55)	2.14E-04
rs146613681	PLEKHS1	10	113770946	0.0047	G	1.96 (1.36, 2.82)	2.84E-04
rs12571587	ABLIM1	10	114655839	0.367	T	1.15 (1.08, 1.22)	7.21E-06
rs188497266	GRK5	10	119435675	0.0057	A	1.89 (1.34, 2.65)	2.42E-04
rs553537339	MIR4682;PLPP4	10	120143290	0.002	G	2.71 (1.59, 4.61)	2.43E-04
rs375423059	TACC2	10	122113178	0.0071	T	1.86 (1.36, 2.55)	1.17E-04
rs7905027	BTBD16;PLEKHA1	10	122354916	0.3139	G	1.15 (1.08, 1.22)	1.26E-05

rs192246188	PLEKHA1	10	122408972	0.0031	A	2.4 (1.55, 3.7)	7.80E-05
rs59616332	PLEKHA1;ARMS2;LOC105378525	10	122449046	0.4273	GATAAAC	1.26 (1.19, 1.34)	1.46E-14
rs146683256	DMBT1;C10orf120	10	122692241	0.0024	C	2.69 (1.64, 4.39)	8.19E-05
rs192951850	FAM24A;C10orf88	10	122920488	0.0012	A	3.9 (1.96, 7.75)	1.01E-04
rs74633135	CPXM2;CHST15	10	123969394	0.0009	G	4.4 (2.05, 9.46)	1.47E-04
rs1241643022	FAM53B	10	124701194	0.0006	G	6.87 (2.5, 18.89)	1.87E-04
rs11244975	ADAM12	10	126386550	0.1578	A	1.16 (1.07, 1.25)	2.70E-04
rs148561054	C10orf90;DOCK1	10	126713112	0.0065	G	1.87 (1.36, 2.59)	1.36E-04
rs192622178	GLRX3	10	130162466	0.0007	A	4.97 (2.07, 11.92)	3.24E-04
rs1318875097	STK32C	10	132239658	0	T	E+08 (1.31E+04, 1.94E	1.99E-04
rs138908017	IFITM5;IFITM2	11	302191	0.0047	G	0.25 (0.12, 0.52)	2.30E-04
rs1475278890	RPLP2	11	808756	0.0009	G	4.34 (1.97, 9.57)	2.77E-04
rs578073662	LOC105376554;MICAL2	11	12072980	0.0007	C	5.23 (2.15, 12.7)	2.59E-04
rs141745114	SPON1	11	14015817	0.002	G	2.9 (1.71, 4.91)	7.64E-05
rs56899596	INSC	11	15243902	0.0507	TC	1.29 (1.13, 1.47)	1.39E-04
rs79498181	INSC;LOC102724957	11	15406101	0.0159	G	0.6 (0.45, 0.79)	3.08E-04
rs7110213	LOC102724957;SOX6	11	15719929	0.3743	A	1.14 (1.07, 1.22)	3.05E-05
rs1457461717		11	19104850	0.0006	A	6.36 (2.45, 16.5)	1.44E-04
rs61876764	CSRP3;E2F8	11	19220418	0.2446	T	0.87 (0.81, 0.94)	1.82E-04
rs4757862	NAV2	11	19931118	0.3596	G	1.12 (1.05, 1.19)	3.19E-04
rs188712137	ANO5	11	22278418	0.0031	C	2.33 (1.5, 3.63)	1.76E-04
rs1217086993		11	23783670	0.0018	C	3.03 (1.72, 5.33)	1.20E-04
rs923606173	LGR4	11	27463205	0.0034	T	2.23 (1.46, 3.42)	2.06E-04
rs569899557	MIR8068;LINC01616	11	28717506	0.0014	A	3.69 (1.93, 7.03)	7.38E-05
rs2711825	MIR8068;LINC01616	11	29415208	0.4013	T	0.89 (0.84, 0.95)	2.81E-04
rs78034647	MIR8068;LINC01616	11	29817314	0.0155	C	1.66 (1.34, 2.05)	2.59E-06
rs371459655	MPPED2	11	30453581	0.0014	T	3.43 (1.8, 6.54)	1.88E-04
rs143059563	PAX6-AS1;LOC100506675	11	32016420	0.0005	G	12.68 (3.59, 44.82)	8.07E-05
rs12275889	SNORA88;WT1	11	32206437	0.0027	G	2.46 (1.53, 3.95)	1.89E-04
rs181578329	SNORA88;WT1	11	32370496	0.0007	G	7.67 (2.96, 19.88)	2.75E-05
rs961772900	WT1-AS	11	32445125	0.0006	A	5.88 (2.23, 15.5)	3.35E-04
rs574271118	C11orf74	11	36625523	0.0016	G	2.94 (1.63, 5.31)	3.45E-04
rs979957436	C11orf74;LOC105376633	11	36877626	0.0007	T	5.23 (2.16, 12.69)	2.49E-04
rs914863719	C11orf74;LOC105376633	11	37422948	0.0015	G	3.08 (1.69, 5.62)	2.50E-04
rs144717688	C11orf74;LOC105376633	11	37589048	0.0012	C	3.54 (1.79, 6.98)	2.68E-04
rs79613004	C11orf74;LOC105376633	11	37627801	0.046	A	1.3 (1.13, 1.48)	1.25E-04
rs553932355	LRRC4C	11	41073547	0.0044	G	2.11 (1.45, 3.08)	9.64E-05

rs957462372	LOC101928591	11	42052907	0.0046	G	2.13 (1.48, 3.07)	4.32E-05
rs180827056	LOC100507205;HNRNPKP3	11	42302096	0.0013	A	3.24 (1.71, 6.16)	3.21E-04
rs1194449714		11	42597875	0.0012	T	3.84 (1.97, 7.48)	7.46E-05
rs147241024	ALX4;CD82	11	44438862	0.0146	C	1.52 (1.21, 1.9)	2.87E-04
rs374014523	LOC399886;CHST1	11	45497026	0.0046	G	1.99 (1.37, 2.87)	2.67E-04
rs755065585	LRP4	11	46875760	0.0011	C	4.58 (2.26, 9.31)	2.55E-05
rs61897791	ARFGAP2	11	47165010	0.0651	T	0.78 (0.68, 0.88)	1.02E-04
rs911019223	CELF1	11	47471855	0.0013	G	4.47 (2.34, 8.55)	5.98E-06
rs201679932	OR4D11;OR4D9	11	59512171	0.0022	G	2.75 (1.65, 4.58)	9.72E-05
rs1307721204	CD6	11	61011998	0	A	E+07 (2.87E+03, 6.64E	1.44E-04
rs1174530061	VPS37C	11	61161422	0.0019	G	2.8 (1.63, 4.83)	2.07E-04
rs1052101693	MYRF	11	61762882	0	C	E+08 (2.33E+04, 3.31E	1.28E-04
rs2269400	LGALS12	11	63510858	0.2866	C	0.87 (0.82, 0.93)	5.95E-05
rs150240569	LGALS12;RARRES3	11	63532362	0.0172	C	0.57 (0.43, 0.75)	8.47E-05
rs185287594	TMEM262;VPS51	11	65090530	0.0198	G	1.56 (1.3, 1.88)	2.57E-06
rs56890194		11	66320187	0.1286	T	1.2 (1.1, 1.31)	3.27E-05
rs113835537	CTSF;CCDC87	11	66572168	0.1721	A	1.16 (1.08, 1.25)	1.25E-04
rs141351988	PC	11	66873349	0.156	T	1.17 (1.08, 1.26)	1.31E-04
rs479763	ALDH3B1	11	68007658	0.0001	T	E+07 (3.15E+03, 4.04E	2.61E-04
rs569297026	CHKA	11	68114292	0.0027	C	2.45 (1.54, 3.89)	1.43E-04
rs1330190783	SPCS2	11	74948101	0.0041	G	2.16 (1.47, 3.18)	9.48E-05
rs750823245	OR2AT4;SLCO2B1	11	75105912	0.0013	G	3.37 (1.76, 6.46)	2.49E-04
rs7940138	UVRAG;WNT11	11	76163993	0.1666	C	0.84 (0.77, 0.91)	6.80E-05
rs919831909	WNT11;THAP12	11	76228845	0.004	G	2.11 (1.42, 3.12)	1.92E-04
rs116970193	GDPD4	11	77234738	0.1092	A	1.22 (1.11, 1.34)	2.39E-05
rs142042401	GDPD4;PAK1	11	77319892	0.0023	C	2.82 (1.71, 4.63)	4.55E-05
rs117934430	PAK1	11	77369960	0.0374	T	1.39 (1.2, 1.61)	1.21E-05
rs186212077	PAK1	11	77448473	0.004	T	2.09 (1.41, 3.09)	2.30E-04
rs145937686	AAMDC	11	77835142	0.0011	G	3.79 (1.84, 7.8)	2.93E-04
rs80300633	TENM4;LOC101928944	11	80635244	0.006	A	1.84 (1.32, 2.57)	3.31E-04
rs75210751	LOC101928944;MIR4300HG	11	80873474	0.0545	C	1.25 (1.11, 1.41)	3.07E-04
rs577096795	LOC101928944;MIR4300HG	11	80970619	0.0013	T	3.38 (1.74, 6.56)	3.16E-04
rs148825112	DLG2	11	84445092	0.0827	G	0.78 (0.69, 0.88)	5.74E-05
rs1029568761	DISC1FP1;FAT3	11	92022471	0.0017	T	2.89 (1.63, 5.13)	2.89E-04
rs571977046	SLC36A4;DEUP1	11	93302838	0.0042	T	2.19 (1.5, 3.21)	5.23E-05

rs540037397	LOC100129203;FAM76B	11	95333163	0.0006	C	8.27 (2.95, 23.19)	5.85E-05
rs138438759	LOC105369443;CNTN5	11	97518739	0.0007	C	5.89 (2.41, 14.41)	1.02E-04
rs78504140	NONE;CNTN5	11	99018417	0.0356	A	0.69 (0.57, 0.83)	7.54E-05
rs778209690	CNTN5;LOC100128386	11	100542235	0.0012	T	4 (2.06, 7.77)	4.15E-05
rs145878599	MMP3;MMP12	11	102854879	0.0336	A	0.71 (0.6, 0.85)	2.44E-04
rs1359700699		11	106469647	0.0012	CAA	3.58 (1.81, 7.08)	2.49E-04
rs2513131	GUCY1A2;CWF19L2	11	107059830	0.1498	A	1.17 (1.07, 1.27)	2.78E-04
rs7926110	GUCY1A2;CWF19L2	11	107215417	0.4917	T	0.89 (0.83, 0.94)	4.70E-05
rs1328944662		11	110072957	0.0014	T	3.45 (1.83, 6.47)	1.21E-04
rs190838608	FDX1;ARHGAP20	11	110533882	0.0033	T	2.21 (1.45, 3.39)	2.52E-04
rs529163168	ARHGAP20;C11orf53	11	111251515	0.0086	T	1.91 (1.45, 2.53)	5.43E-06
rs7107378	C11orf88	11	111531115	0.2255	C	1.13 (1.06, 1.22)	3.11E-04
rs137962870	LOC283140;LOC101928823	11	112397196	0.0273	G	1.4 (1.18, 1.65)	1.16E-04
rs151012650	NCAM1	11	113126271	0.0557	G	1.27 (1.12, 1.44)	1.46E-04
rs181643968	CADM1	11	115493938	0.0049	A	2.02 (1.41, 2.89)	1.24E-04
rs149845525	LINC00900;LOC101929011	11	116437920	0.0237	G	1.46 (1.22, 1.75)	2.65E-05
rs3824882	TRIM29	11	120124579	0.166	T	0.86 (0.79, 0.93)	2.70E-04
rs902530190	TECTA;SC5D	11	121221155	0.0004	G	11.6 (3.2, 42.02)	1.89E-04
rs539497027	CLMP;MIR4493	11	123277071	0.0005	A	7.82 (2.58, 23.66)	2.74E-04
rs560416706	CDON;RPUSD4	11	126198698	0.0013	T	4.22 (2.21, 8.06)	1.27E-05
rs142511572	LOC101929497;LINC02098	11	127495767	0.003	C	2.53 (1.6, 4)	7.86E-05
rs7932045	ETS1;LOC101929538	11	128625211	0.4591	C	1.15 (1.08, 1.22)	7.83E-06
rs531726609	SNX19;NTM	11	131054350	0.0009	A	4.4 (1.98, 9.79)	2.87E-04
rs1456937219	ERC1	12	1162519	0.0019	G	3.14 (1.83, 5.39)	3.32E-05
rs1462371264	ERC1	12	1458969	0.0018	CACGTGATAATCCCAGAACAGTTTTAT	3.46 (2, 6)	9.67E-06
rs192038486	NANOG;SLC2A14	12	7809292	0.0036	C	2.26 (1.51, 3.39)	7.51E-05
rs370041088	CLEC4A	12	8135175	0.0006	C	6.26 (2.42, 16.24)	1.60E-04
rs183751108	CD69	12	9761891	0.003	A	2.29 (1.46, 3.61)	3.37E-04
rs533971307	LRP6	12	12219420	0.0035	G	2.34 (1.55, 3.54)	5.38E-05
rs6488810	DERA	12	16003670	0.4176	T	0.89 (0.84, 0.95)	2.10E-04
rs541944322	LMO3;SKP1P2	12	16857005	0.0005	C	8.12 (2.77, 23.8)	1.36E-04
rs952810519	SKP1P2;LINC02378	12	17130208	0.0005	A	8.62 (2.89, 25.74)	1.14E-04
rs937326504	SLCO1B7	12	21041862	0.0006	G	6 (2.28, 15.76)	2.79E-04

rs183075030	ETNK1;LOC101928441	12	22926561	0.0056	T	0.35 (0.2, 0.62)	3.27E-04
chr12:29495061_GA_G		12	29495061	0.1651	GA	0.86 (0.79, 0.93)	3.29E-04
rs7301855	TMTC1	12	29508540	0.3553	G	1.13 (1.07, 1.2)	4.59E-05
rs543313894	TMTC1	12	29680896	0.0007	G	5.1 (2.11, 12.32)	2.93E-04
rs16935254	TMTC1;IPO8	12	30011755	0.1709	A	0.86 (0.79, 0.93)	3.33E-04
rs181930445	TMTC1;IPO8	12	30174300	0.001	C	4.06 (1.94, 8.54)	2.11E-04
chr12:37820790_C_CA		12	37820790	0.0007	C	7.15 (2.93, 17.46)	1.56E-05
rs1371923603		12	39183266	0.0008	G	4.83 (2.07, 11.24)	2.59E-04
rs150018586	MUC19;CNTN1	12	40595616	0.0125	T	0.55 (0.4, 0.76)	2.72E-04
rs183303256	LINC02450;LINC02461	12	42844070	0.0066	T	1.85 (1.35, 2.53)	1.42E-04
rs78547031	LINC02461;ADAMTS20	12	43278674	0.0161	C	1.49 (1.21, 1.85)	2.44E-04
rs961701010	ADAMTS20;PUS7L	12	43652923	0.0009	A	4.3 (2.01, 9.22)	1.79E-04
rs976664500	TMEM117	12	43904017	0.0009	G	4.62 (2.1, 10.15)	1.38E-04
rs971960087	TMEM117	12	44361111	0.0007	A	6.21 (2.53, 15.23)	6.72E-05
rs936394634	LINC02396	12	49908030	0.0007	C	5.72 (2.29, 14.31)	1.89E-04
rs17124514	LIMA1	12	50186613	0.1608	A	0.83 (0.77, 0.91)	1.86E-05
rs144292402	AAAS	12	53311772	0.0372	G	1.33 (1.14, 1.54)	2.04E-04
chr12:57383717_A_AAAT		12	57383717	0.0044	A	2.13 (1.47, 3.09)	5.89E-05
rs141361562	OS9	12	57722000	0.0085	G	1.81 (1.37, 2.39)	3.48E-05
rs567627695	ATP23;LINC02403	12	57990940	0.0054	C	2.19 (1.57, 3.05)	4.16E-06
rs981903405	TBK1	12	64471714	0.0007	A	5.16 (2.12, 12.53)	2.94E-04
rs532982828	PTPRR	12	70828267	0.0013	T	3.4 (1.78, 6.51)	2.15E-04
rs377567692	TSPAN8;LGR5	12	71287730	0.0008	A	4.48 (1.98, 10.15)	3.27E-04
rs1219852709		12	72084614	0.0016	T	3.02 (1.67, 5.46)	2.51E-04
rs535526429	TRHDE	12	72289395	0.0014	A	3.31 (1.8, 6.11)	1.24E-04
rs1026279094	ATXN7L3B;KCNC2	12	74677930	0.0067	TC	1.77 (1.3, 2.41)	3.07E-04
rs188670151	KRR1;PHLDA1	12	75793287	0.0011	G	3.66 (1.83, 7.35)	2.57E-04
rs189634241	LIN7A;ACSS3	12	80980347	0.0064	G	1.85 (1.34, 2.54)	1.73E-04
rs567908680	TMTC2	12	83035659	0.0011	T	4.44 (2.14, 9.19)	6.06E-05
rs117511182	ALX1;RASSF9	12	85611146	0.0053	A	1.89 (1.34, 2.67)	2.80E-04
rs183752112	TMTC3	12	88182035	0.0032	A	2.33 (1.51, 3.59)	1.23E-04
rs1395669628		12	89270474	0.0018	TAA	2.79 (1.6, 4.87)	3.07E-04
rs960391571	SOCS2;CRADD	12	93593777	0.0021	T	2.9 (1.73, 4.88)	5.67E-05
rs572844034	KRT19P2;NDUFA12	12	94967751	0.0014	G	3.7 (1.96, 6.98)	5.37E-05
rs1448547904		12	95465311	0.0012	C	4.37 (2.19, 8.74)	3.01E-05
rs567668909	LOC643711;MIR4495	12	97929823	0.0019	T	2.73 (1.58, 4.71)	3.06E-04

rs1480069	MIR4303;SLC9A7P1	12	98164711	0.0067	C	0.34 (0.2, 0.58)	8.97E-05
rs180685968	IGF1;LINC00485	12	102583002	0.0022	C	2.78 (1.65, 4.68)	1.15E-04
rs375638450	IGF1;LINC00485	12	102804935	0.0027	T	3.25 (2.07, 5.12)	3.14E-07
rs79081906	STAB2	12	103752052	0.1006	C	0.83 (0.75, 0.92)	3.18E-04
rs574447287	GLT8D2	12	104032734	0.0087	A	0.45 (0.3, 0.69)	2.07E-04
rs191975382	KCCAT198	12	105316021	0.0015	G	3.24 (1.75, 6)	1.93E-04
rs34314798	NUAK1;CKAP4	12	106160663	0.0177	C	1.59 (1.3, 1.94)	4.98E-06
rs142705731	MYO1H	12	109418782	0.0412	G	0.74 (0.62, 0.87)	2.42E-04
rs76310217	RBM19	12	113935221	0.0083	G	0.44 (0.28, 0.67)	1.75E-04
rs191729636	RBM19	12	113939264	0.0018	C	3 (1.72, 5.24)	1.12E-04
rs61453819	TBX5-AS1;TBX3	12	114421679	0.1553	C	0.85 (0.78, 0.93)	2.40E-04
rs746683085	MAP1LC3B2;C12orf49	12	116601115	0.0009	ACT	5.36 (2.41, 11.89)	3.67E-05
rs926597289	MAP1LC3B2;C12orf49	12	116679957	0.0014	A	3.47 (1.83, 6.58)	1.35E-04
rs76845667	HRK;FBXW8	12	116896682	0.2017	C	0.87 (0.81, 0.94)	2.93E-04
rs188593554	NOS1;KSR2	12	117382619	0.0014	G	3.55 (1.87, 6.71)	9.98E-05
rs183854946	LINC02439;SRRM4	12	118915616	0.0038	A	2.11 (1.41, 3.16)	2.67E-04
rs201061093	MLEC	12	120695165	0.0005	C	8.16 (2.78, 23.96)	1.33E-04
rs543257082	XLOC_009911;HNF1A-AS1	12	120959168	0.0006	A	6.38 (2.45, 16.62)	1.46E-04
rs370175973	LINC02347;LOC100996671	12	126569924	0.0032	AG	2.36 (1.53, 3.65)	1.15E-04
rs11611258	LINC02405	12	126943083	0.0392	A	1.31 (1.13, 1.51)	2.07E-04
rs116914987	LINC02405;LINC02376	12	127065452	0.0019	G	2.73 (1.58, 4.72)	3.20E-04
rs1334828736	TMEM132C	12	128599555	0.0006	T	10.71 (3.66, 31.32)	1.47E-05
rs373573371	TMEM132C	12	128600481	0.0014	A	4.17 (2.24, 7.76)	6.54E-06
rs551152033	SLC15A4;GLT1D1	12	128848159	0.0025	G	2.61 (1.6, 4.27)	1.31E-04
rs549799961	TMEM132D;LINC02418	12	130015788	0.0006	C	6.89 (2.42, 19.64)	3.06E-04
rs371429489	LINC00408;LINC00442	13	19000938	0.0017	C	2.97 (1.67, 5.29)	2.13E-04
rs148552975	LINC00442;LOC107984132	13	19030305	0.0028	G	2.58 (1.64, 4.07)	4.38E-05
rs199531845	PARP4	13	24427903	0.0665	TTAA	1.24 (1.11, 1.39)	1.87E-04
rs540896208	NUP58	13	25317915	0.0024	A	2.66 (1.62, 4.38)	1.22E-04
rs117633200	PDS5B	13	32713927	0.0149	T	0.57 (0.43, 0.77)	2.50E-04
rs1198827502	KL	13	33026445	0.001	G	5.23 (2.5, 10.94)	1.10E-05
rs1426945574	SOHLH2	13	36190491	0.0006	T	7.29 (2.71, 19.61)	8.41E-05
rs9548013	TRPC4	13	37678408	0.0395	A	1.3 (1.13, 1.5)	3.45E-04
rs1230276683		13	40066681	0.0007	C	7.38 (2.87, 19)	3.42E-05
rs7336157	LINC00548;LINC00598	13	40345788	0.0387	C	1.31 (1.14, 1.51)	1.65E-04

rs190030205	LINC00598;FOXO1	13	40511551	0.0277	T	1.36 (1.15, 1.61)	3.23E-04
rs142610368	AKAP11;LINC02341	13	42368462	0.002	G	2.97 (1.75, 5.02)	5.32E-05
rs117734179	ENOX1	13	43482179	0.0019	T	2.76 (1.6, 4.75)	2.63E-04
rs1025207465	MED4;ITM2B	13	48149877	0.001	A	4.92 (2.29, 10.56)	4.29E-05
rs477620	MIR5007;PRR20B	13	55683674	0.1527	A	0.85 (0.79, 0.93)	2.26E-04
rs118033004	PRR20E;PCDH17	13	57473567	0.0645	C	1.26 (1.13, 1.41)	4.70E-05
rs183395623	DIAPH3	13	59702762	0.0029	T	2.53 (1.61, 3.99)	6.25E-05
rs541498072	LINC00378	13	60681091	0.0015	C	3.18 (1.7, 5.92)	2.78E-04
rs1242719033	LOC105370245	13	66090117	0.0012	G	3.72 (1.89, 7.33)	1.50E-04
rs61951540	LINC00364;LINC00550	13	68778651	0.177	C	1.15 (1.07, 1.24)	2.25E-04
rs1474747670		13	74805914	0.0009	T	4.9 (2.22, 10.83)	8.44E-05
rs79498682	TBC1D4;COMMD6	13	75498668	0.006	C	1.93 (1.38, 2.69)	1.08E-04
chr13:77526321_C_CGT		13	77526321	0.0007	C	5.56 (2.25, 13.76)	2.04E-04
rs118187339	LINC00377;LINC00564	13	81142581	0.0397	A	1.3 (1.13, 1.49)	2.96E-04
rs79170386	LINC00564;SLITRK1	13	81958629	0.005	A	1.94 (1.35, 2.78)	3.14E-04
rs74379160	LINC00564;SLITRK1	13	82103367	0.0184	T	1.44 (1.18, 1.77)	3.31E-04
rs528825937	LINC00564;SLITRK1	13	82216748	0	G	E+10 (4.01E+04, 2.68E	2.87E-04
rs190262745	LINC00564;SLITRK1	13	82745307	0.0031	C	2.34 (1.51, 3.63)	1.46E-04
rs1170099070		13	86348183	0.0013	C	3.4 (1.76, 6.59)	2.84E-04
rs1376886749		13	86712138	0.0012	AT	3.61 (1.85, 7.05)	1.72E-04
rs533947697	SLITRK6;LINC00430	13	86855117	0.0035	G	2.36 (1.57, 3.55)	3.77E-05
chr13:87555891_C_CTA		13	87555891	0.0012	C	3.89 (1.99, 7.57)	6.70E-05
rs143776983	LINC00353;LINC00559	13	89615222	0.0026	T	2.52 (1.56, 4.06)	1.48E-04
rs551569118	GPC5	13	91794410	0.0015	G	3.14 (1.7, 5.8)	2.51E-04
rs543468958	GPC5	13	92311793	0.0047	A	2.03 (1.4, 2.94)	1.84E-04
rs9524526	DCT	13	94498218	0.383	T	0.89 (0.84, 0.95)	3.31E-04
rs76154541	LOC101927284	13	94823799	0.0096	G	0.48 (0.32, 0.71)	2.69E-04
rs1484974057	ABCC4	13	95259005	0.0009	CAG	4.17 (1.94, 8.97)	2.58E-04
rs73555775	MBNL2	13	97261051	0.0085	T	1.68 (1.27, 2.23)	2.97E-04
rs192026615	RAP2A;IPO5	13	97862700	0.0011	A	4.01 (1.94, 8.29)	1.72E-04
rs142487947	IPO5;FARP1	13	98039787	0.0045	G	2.05 (1.4, 3)	2.02E-04
rs79536795	UBAC2	13	99267567	0.0468	A	1.29 (1.13, 1.47)	1.74E-04
rs186293576	UBAC2	13	99327605	0.0012	G	3.94 (1.98, 7.83)	9.06E-05
rs142395613	NALCN	13	101123442	0.0187	G	1.51 (1.24, 1.83)	3.00E-05
rs151016745	LINC01309;DAOA-AS1	13	105410955	0.0063	C	1.89 (1.36, 2.64)	1.69E-04
rs146103575	TNFSF13B;MYO16	13	108520214	0.0008	G	4.66 (2.04, 10.64)	2.64E-04

rs145333280	MYO16	13	108900208	0.0088	A	1.85 (1.41, 2.42)	9.08E-06
rs142839276	COL4A2	13	110398399	0.0025	T	2.59 (1.59, 4.22)	1.30E-04
rs376822011	LINC00403	13	112013432	0.0017	C	3.49 (1.96, 6.22)	2.25E-05
rs373910365	LINC00403;LOC100506016	13	112112749	0.0012	G	3.66 (1.85, 7.26)	2.00E-04
rs9550050	LINC01044	13	112329661	0.4707	C	1.13 (1.06, 1.2)	1.20E-04
rs17291215	CDC16	13	114245828	0.0327	C	1.36 (1.17, 1.59)	7.50E-05
rs147943349	OR4E1;LINC02332	14	22247887	0.0416	G	1.33 (1.16, 1.52)	3.91E-05
rs1320094190	EFS	14	23366313	0.0011	A	3.73 (1.83, 7.61)	2.83E-04
rs186457931	DCAF11	14	24116565	0.0021	G	2.58 (1.54, 4.33)	3.17E-04
rs181697020	ADCY4	14	24330715	0.0015	C	3.14 (1.71, 5.76)	2.28E-04
rs142617063	KHNYN	14	24430153	0.1401	G	1.17 (1.07, 1.27)	2.86E-04
rs186163869	STXBP6;NOVA1	14	26106166	0.0014	T	3.23 (1.73, 6.03)	2.41E-04
rs191167038	LINC00645;FOXG1-AS1	14	28150563	0.0019	G	3.18 (1.83, 5.52)	3.77E-05
rs200616389		14	28408160	0.0018	GA	3.07 (1.76, 5.36)	7.58E-05
rs375596853	LINC01551;PRKD1	14	29543906	0.0012	G	3.59 (1.81, 7.13)	2.54E-04
rs61754460	HEATR5A	14	31293936	0.0991	T	0.81 (0.73, 0.9)	1.51E-04
rs17101068	NPAS3	14	33420800	0.0393	C	1.29 (1.12, 1.49)	3.10E-04
rs148514263	NFKBIA;INSM2	14	35491709	0.0046	G	2.03 (1.4, 2.94)	1.89E-04
rs547825256	LINC00609	14	36087674	0.0029	T	2.32 (1.46, 3.68)	3.38E-04
rs73298988	LINC02315	14	41120565	0.0043	A	2.2 (1.5, 3.22)	4.97E-05
rs8008330	LINC02315;LRFN5	14	41348366	0.4256	T	1.11 (1.05, 1.18)	3.01E-04
rs371363272	LRFN5;NONE	14	41965937	0.0004	C	14.99 (3.78, 59.42)	1.17E-04
rs149451753	LRFN5;FSCB	14	42636798	0.0006	G	9.61 (3.39, 27.27)	2.09E-05
rs184012229	LRFN5;FSCB	14	43107180	0.0092	A	1.67 (1.28, 2.18)	1.64E-04
rs2208777	NONE;FSCB	14	43958615	0.4957	C	0.88 (0.83, 0.94)	2.92E-05
rs73291673	SOS2	14	50230080	0.4874	A	0.89 (0.83, 0.94)	5.80E-05
rs2993998	FRMD6;GNG2	14	51854386	0.3977	C	1.12 (1.05, 1.19)	3.41E-04
rs376725161	LOC101927620;MIR5580	14	53904831	0.0006	T	6.52 (2.44, 17.4)	1.84E-04
rs751099044	LINC00520;PELI2	14	55975383	0.0008	G	4.88 (2.1, 11.34)	2.27E-04
rs1202521336	LOC124903323	14	57003313	0.0036	C	2.18 (1.45, 3.27)	1.79E-04
rs372492707	KIAA0586;DACT1	14	58577486	0.0009	A	4.73 (2.17, 10.31)	9.21E-05
rs1226470830	SPTB	14	64857463	0.0008	T	4.79 (2.07, 11.11)	2.56E-04
rs1391194710		14	65350980	0.0007	A	6.16 (2.46, 15.45)	1.04E-04
rs1290594056	FUT8	14	65646399	0.0007	A	5.43 (2.23, 13.22)	1.93E-04
rs77783456	TMEM229B	14	67531975	0.0166	G	1.56 (1.26, 1.93)	5.40E-05
chr14:67912708_G_GT		14	67912708	0.0009	G	4.32 (1.94, 9.59)	3.30E-04

rs1301748054	RAD51B	14	68292888	0.0007	G	5.02 (2.08, 12.14)	3.37E-04
rs7145174	MAP3K9;PCNX1	14	70820051	0.0339	G	1.39 (1.19, 1.61)	1.87E-05
rs550425611	SYNDIG1L	14	74405230	0.0009	C	4.35 (1.99, 9.53)	2.39E-04
rs376049211	PGF;EIF2B2	14	74971367	0.0013	G	3.63 (1.88, 6.99)	1.20E-04
rs767866659	JDP2	14	75444629	0.0055	CT	1.86 (1.33, 2.62)	3.40E-04
rs147353867	IFT43	14	76069464	0.0055	A	1.96 (1.39, 2.74)	1.06E-04
rs1483291609	NRXN3	14	78402842	0.0005	C	9.54 (2.94, 30.97)	1.73E-04
rs944593967	NRXN3	14	78816951	0.0023	C	2.56 (1.57, 4.19)	1.71E-04
rs182324369	NRXN3	14	79097669	0.0028	C	2.41 (1.53, 3.79)	1.58E-04
rs1357118033	NRXN3	14	79102814	0.0024	G	2.51 (1.54, 4.08)	2.22E-04
rs150485399	DIO2-AS1	14	80453442	0.0024	T	2.49 (1.53, 4.05)	2.47E-04
rs72703728	STON2	14	81368179	0.2674	G	0.88 (0.82, 0.94)	2.66E-04
rs1240864268		14	81546528	0.005	A	1.95 (1.37, 2.78)	1.91E-04
rs77976400	LINC02301;NONE	14	82766738	0.008	G	1.8 (1.35, 2.4)	7.24E-05
rs1230173568		14	82836006	0.0004	A	17.03 (3.81, 76.19)	2.09E-04
rs1959455	FLRT2;LINC02328	14	85883148	0.3675	G	1.12 (1.05, 1.19)	2.53E-04
rs79651585	FLRT2;LINC02328	14	85924128	0.1325	T	1.17 (1.07, 1.27)	2.93E-04
rs761762826	LINC02328;LINC01148	14	86384286	0.0008	C	4.97 (2.12, 11.65)	2.23E-04
rs1250474233	FOXN3	14	89473396	0.0014	TCC	3.22 (1.71, 6.08)	2.99E-04
rs117820599	DIGIT;DICER1	14	94904852	0.0156	G	1.49 (1.2, 1.85)	3.31E-04
rs150492086	TCL1A;TUNAR	14	95794094	0.0011	C	3.58 (1.79, 7.2)	3.32E-04
rs3783360	MEG3	14	100855838	0.007	G	1.8 (1.32, 2.45)	1.94E-04
rs73358304	ATP10A;LINC02346	15	25886075	0.0268	C	1.36 (1.15, 1.61)	3.04E-04
rs11633775	LINC00929;GABRB3	15	26256085	0.0049	G	1.94 (1.35, 2.78)	3.23E-04
rs529996373	LINC00929;GABRB3	15	26393354	0.0012	G	4.04 (2, 8.15)	9.50E-05
rs67250426	GABRG3	15	27372758	0.0009	A	5.07 (2.31, 11.15)	5.41E-05
chr15:31478348_G_GTA		15	31478348	0.354	G	0.87 (0.82, 0.93)	4.63E-05
rs982809059	CHRM5	15	34053105	0.0031	G	2.37 (1.53, 3.68)	1.15E-04
rs1035512659	C15orf53;C15orf54	15	38755094	0.0013	G	3.59 (1.88, 6.88)	1.12E-04
rs370649131	CDAN1	15	42734465	0.0024	T	2.61 (1.58, 4.31)	1.75E-04
rs45456398	LCMT2	15	43327779	0.0007	A	5.06 (2.09, 12.26)	3.25E-04
rs8039597	SEMA6D	15	47611450	0.4728	G	1.12 (1.06, 1.19)	1.66E-04
rs377424518	WDR72	15	53727412	0.013	G	1.64 (1.31, 2.06)	2.20E-05
rs992235288	BNIP2;FOXB1	15	59928564	0.0006	C	6.87 (2.55, 18.51)	1.40E-04
rs894701924	RORA-AS1	15	60526579	0.0006	C	7.17 (2.54, 20.23)	1.98E-04
rs12593278	LINC02349;VPS13C	15	61837610	0.4764	G	0.89 (0.84, 0.95)	3.15E-04
rs72741190	TLN2;TPM1	15	63018412	0.0117	A	1.58 (1.23, 2.02)	2.74E-04

rs200222914	CLPX	15	65185031	0.001	C	4.01 (1.9, 8.44)	2.62E-04
rs4776811	LINC01169	15	66649815	0.1691	T	1.16 (1.08, 1.25)	1.34E-04
rs560249431	THSD4	15	71642984	0.0032	G	2.24 (1.45, 3.48)	2.88E-04
rs560113488	MYO9A	15	71836382	0.0019	G	4.05 (2.36, 6.97)	4.21E-07
rs561105124	MYO9A	15	72021873	0.0016	C	3.05 (1.66, 5.58)	3.06E-04
rs1454738593	GRAMD2A	15	72171774	0.0016	TTC	4.03 (2.27, 7.15)	2.04E-06
rs181335347	ARIH1	15	72492871	0.0022	A	2.74 (1.64, 4.56)	1.10E-04
rs1421564186	IL16	15	81282489	0.0023	C	2.65 (1.59, 4.39)	1.69E-04
rs79463554	TMC3-AS1;MEX3B	15	81634595	0.0308	G	1.34 (1.14, 1.56)	3.23E-04
rs181812977	SLC28A1	15	84932745	0.0061	G	1.96 (1.42, 2.71)	5.06E-05
rs146970100	PDE8A	15	85112049	0.287	TACTC	0.87 (0.81, 0.93)	1.06E-04
rs1280853510	LOC107983974	15	93570279	0.0014	A	3.24 (1.72, 6.08)	2.62E-04
rs1186764287		15	94514929	0.003	A	2.55 (1.63, 3.98)	3.92E-05
rs979526105	NR2F2-AS1	15	96308746	0.0003	G	19.06 (3.85, 94.24)	3.01E-04
rs77538792	NR2F2;SPATA8-AS1	15	96376503	0.0818	A	0.81 (0.73, 0.91)	2.99E-04
rs182022344	SPATA8;LINC02254	15	96937897	0.0024	G	2.61 (1.59, 4.27)	1.39E-04
rs1199471849	LINC02253	15	97332672	0.0021	C	2.77 (1.65, 4.67)	1.28E-04
rs747400416		15	97567707	0.0004	G	15.36 (3.64, 64.73)	1.98E-04
rs546511986	LINC00923	15	97858025	0.0007	A	5.09 (2.11, 12.29)	2.92E-04
rs1017482632	TTC23	15	99238842	0.0014	G	3.36 (1.79, 6.33)	1.71E-04
rs190266357	ADAMTS17	15	100325830	0.0031	C	2.34 (1.5, 3.66)	1.76E-04
rs140809092	MIR8065;RBFOX1	16	5783026	0.0028	C	2.33 (1.47, 3.71)	3.38E-04
rs185724912	MIR8065;RBFOX1	16	5999662	0.0141	C	1.57 (1.25, 1.97)	1.21E-04
rs537186555	RBFOX1	16	6225174	0.0022	A	2.67 (1.58, 4.52)	2.46E-04
rs575387117	METTL22	16	8631701	0.0005	A	9.09 (2.8, 29.5)	2.36E-04
rs80055513	LOC101927131;LITAF	16	11500833	0.009	G	0.4 (0.26, 0.62)	4.01E-05
rs735809	SNX29	16	12355479	0.0903	T	1.21 (1.09, 1.33)	2.82E-04
rs140218542	C16orf45	16	15511324	0.0028	C	2.34 (1.48, 3.71)	2.87E-04
rs139069602	XYLT1;NPIPA8	16	18158377	0.0037	G	2.27 (1.52, 3.41)	7.02E-05
rs368248553	GPR139	16	20069916	0.0013	G	3.82 (2, 7.27)	4.58E-05
rs913564852	VWA3A;EEF2K	16	22161698	0.0009	G	4.97 (2.29, 10.79)	4.90E-05
rs376294668	LOC653786;HS3ST2	16	22667741	0.0007	T	6.96 (2.81, 17.23)	2.73E-05
rs373030157	HS3ST2	16	22917173	0.0014	A	3.45 (1.86, 6.4)	8.75E-05
rs181945005	PRKCB	16	23853047	0.004	A	2.1 (1.4, 3.14)	3.01E-04
rs545894459	SNX29P2	16	29314465	0.0012	G	3.46 (1.77, 6.74)	2.69E-04
rs1388865714		16	36223522	0.0027	T	2.47 (1.54, 3.97)	1.76E-04
rs143635481	MIR5095	16	48314377	0.0069	C	1.8 (1.33, 2.45)	1.59E-04
rs1000927140	LINC02178;NKD1	16	50446184	0.0033	G	2.33 (1.52, 3.57)	1.07E-04

chr16:50955879_CT_C		16	50955879	0.1671	CT	1.16 (1.07, 1.25)	2.02E-04
rs78312171	SALL1;LINC01571	16	51556012	0.0643	T	1.23 (1.1, 1.38)	2.89E-04
rs79692563	LINC02140;LOC101927480	16	54600835	0.0066	A	1.85 (1.35, 2.54)	1.40E-04
rs188599606	CDH8;CDH11	16	63664437	0.0184	G	1.45 (1.19, 1.77)	2.68E-04
rs575575648	NONE;CDH11	16	64765727	0.0008	C	5.17 (2.17, 12.29)	2.02E-04
rs1005363086	LINC00922	16	65525270	0.0015	G	3.72 (2.01, 6.88)	2.82E-05
rs200360462	PKD1L3	16	71978303	0.0035	G	2.19 (1.44, 3.33)	2.55E-04
rs551044251	HPR;TXNL4B	16	72081708	0.0009	C	4.67 (2.17, 10.08)	8.53E-05
rs200392022	LINC01572	16	72521471	0.0047	A	2 (1.39, 2.87)	2.06E-04
rs1187444327		16	74711633	0.0007	G	6.76 (2.67, 17.14)	5.56E-05
rs183679718	LINC02170	16	80876581	0.0043	T	2.25 (1.55, 3.29)	2.41E-05
rs1297492377		16	81050849	0.0009	A	4.09 (1.89, 8.84)	3.42E-04
rs1184734104	MPHOSPH6	16	82160014	0.0012	C	3.84 (1.94, 7.62)	1.19E-04
rs937665136	MPHOSPH6;CDH13	16	82209717	0.0016	G	3 (1.64, 5.47)	3.43E-04
rs112169563	CDH13	16	83258667	0.001	C	4.18 (2.03, 8.57)	9.77E-05
rs181951948	CDH13	16	83457189	0.0042	G	2.18 (1.49, 3.19)	5.46E-05
rs1448348263	CDH13	16	83720117	0.0013	G	3.36 (1.76, 6.42)	2.49E-04
rs1351163335	FANCA	16	89747139	0.0013	AG	3.4 (1.78, 6.48)	2.05E-04
rs958031418	NXN	17	877447	0.0062	C	1.97 (1.44, 2.7)	2.65E-05
rs35619133	TLCD2	17	1706963	0.011	C	1.63 (1.26, 2.1)	2.22E-04
rs28729707	RAP1GAP2	17	2931461	0.4853	G	1.12 (1.05, 1.19)	2.25E-04
rs555890176	SPNS2	17	4510914	0.0025	C	2.5 (1.54, 4.08)	2.34E-04
rs12450512	WSCD1;AIPL1	17	6393360	0.0618	C	1.27 (1.13, 1.43)	8.23E-05
rs185016316	HS3ST3A1	17	13573958	0.0008	G	4.59 (2.01, 10.46)	2.93E-04
rs10775375	HS3ST3A1;CDRT15P1	17	13641405	0.0117	A	1.69 (1.32, 2.16)	3.13E-05
rs1386826487		17	15121215	0.0007	AT	5.44 (2.23, 13.28)	2.03E-04
rs12936228	TEKT3;TVP23C-CDRT4	17	15422219	0.3342	A	0.88 (0.83, 0.94)	1.94E-04
rs909957209	TBC1D27	17	16925133	0.0004	G	9.61 (2.79, 33.11)	3.38E-04
rs1567674801	MPRIP	17	17042436	0.0157	CCCG	1.5 (1.2, 1.86)	3.00E-04
rs1214129392	TVP23B	17	18787417	0.0011	A	5.51 (2.72, 11.17)	2.15E-06
rs545709519	USP22	17	21016078	0.0023	TTTTCGAGTCCTCATATGAATTTTTTCCTTTCTACCATCTAGTTTTCCACA	3.02 (1.85, 4.96)	1.12E-05
rs962005575	MYO1D	17	32573979	0.0093	C	1.66 (1.26, 2.19)	3.01E-04
chr17:35289619_C_CA		17	35289619	0.0257	C	0.65 (0.53, 0.81)	1.04E-04
rs1231468476	AATF	17	36968830	0.0029	G	2.3 (1.46, 3.61)	3.11E-04

rs191653652	DDX52;HNF1B	17	37682544	0.0029	G	2.35 (1.48, 3.72)	2.81E-04
rs181372896	IKZF3	17	39777148	0.0065	T	1.83 (1.33, 2.53)	2.22E-04
rs1351743930	GSDMB	17	39917014	0.0006	GAGA	7.46 (2.56, 21.73)	2.32E-04
rs12949968	KRT27;KRT28	17	40790746	0.3516	T	0.89 (0.83, 0.95)	2.15E-04
rs771031152	KRT39;KRT40	17	40968014	0.0006	G	6.17 (2.39, 15.89)	1.66E-04
rs568337237	ZNF385C;DHX58	17	42050868	0.0015	A	3.16 (1.72, 5.82)	2.20E-04
rs573141745	SP2-AS1	17	47905862	0.0028	G	2.5 (1.57, 3.97)	1.14E-04
rs1168724087		17	49942370	0.0006	G	7.36 (2.71, 19.94)	8.82E-05
rs192279073	TOB1-AS1;SPAG9	17	50942343	0.0074	C	1.81 (1.34, 2.45)	1.26E-04
rs142191566	CA10	17	51726483	0.0008	C	4.59 (2.02, 10.43)	2.73E-04
rs551716753	ANKFN1;NOG	17	56557952	0.001	G	4.15 (1.96, 8.76)	1.97E-04
rs12949044	CUEDC1	17	57923715	0.0156	C	1.53 (1.24, 1.89)	9.05E-05
rs555504772	C17orf64	17	60429708	0.0015	C	4.14 (2.27, 7.56)	3.61E-06
rs1003439175	AXIN2	17	65546182	0.0025	G	2.59 (1.59, 4.23)	1.44E-04
rs9675105	CEP112	17	65920556	0.0447	C	1.32 (1.16, 1.5)	2.13E-05
rs6504402	CEP112	17	66179105	0.0532	T	1.29 (1.14, 1.45)	5.19E-05
rs3764401	APOH;PRKCA	17	66301711	0.0621	T	1.3 (1.16, 1.46)	6.35E-06
rs1219443865	PRKCA	17	66653540	0.001	A	3.92 (1.89, 8.14)	2.50E-04
rs57802998	PRKCA	17	66809055	0.004	G	2.18 (1.47, 3.22)	1.07E-04
rs1017537462	PRKCA;CACNG5	17	66828174	0.0037	T	2.13 (1.41, 3.22)	3.43E-04
rs368087163	HELZ	17	67227548	0.0027	C	2.43 (1.52, 3.88)	2.10E-04
chr17:67304644_ATTGT_A		17	67304644	0.0703	ATTGT	0.79 (0.7, 0.89)	1.54E-04
rs879242975	NOL11	17	67726158	0.0013	T	3.32 (1.74, 6.33)	2.65E-04
rs59956089	BPTF	17	67964738	0.2642	T	1.16 (1.08, 1.24)	3.80E-05
rs201207157	KPNA2;LINC00674	17	68063325	0.0008	T	5.32 (2.32, 12.2)	8.03E-05
rs7350925	LINC00674;LOC440461	17	68176021	0.2966	T	1.13 (1.06, 1.2)	2.56E-04
rs12950972	RHBDF2;CYGB	17	76523218	0.1428	T	0.85 (0.78, 0.93)	3.13E-04
rs993564603	CYGB	17	76532357	0.0005	C	7.42 (2.48, 22.16)	3.32E-04
rs1222619435		17	77622247	0.0023	C	2.7 (1.62, 4.48)	1.34E-04
rs74564781	CBX4;LINC01979	17	79874333	0.1001	T	0.83 (0.75, 0.92)	3.48E-04
rs1368977478	BAHCC1	17	81428038	0.0008	A	5.56 (2.38, 13.01)	7.60E-05
rs8096755	LINC01895;TGIF1	18	3364088	0.0012	G	4.01 (2.02, 7.96)	7.07E-05
rs9965579	LINC01895;TGIF1	18	3381216	0.0292	C	1.39 (1.18, 1.64)	1.09E-04
rs76905185	DLGAP1;LINC01892	18	5054357	0.0549	G	1.26 (1.11, 1.44)	3.48E-04
rs7244648	TMEM200C;L3MBTL4	18	5920978	0.4579	G	1.13 (1.06, 1.2)	1.25E-04
rs371097028	PTPRM;RAB12	18	8468256	0.0041	G	2.2 (1.5, 3.24)	5.99E-05
rs1214630919		18	8606312	0.0014	GA	3.43 (1.81, 6.49)	1.53E-04

rs534233910	LOC101927410	18	10667515	0.0008	A	4.61 (2.01, 10.59)	3.10E-04
rs185718315	ROCK1	18	20998043	0.0023	G	2.91 (1.75, 4.83)	3.55E-05
rs1030375190	NPC1;ANKRD29	18	23591511	0.0008	T	5.14 (2.22, 11.93)	1.36E-04
rs560969348	LINC01894;ZNF521	18	25016627	0.0035	G	2.17 (1.42, 3.31)	3.13E-04
rs181666282	LOC105372038;CDH2	18	27904613	0.0006	A	6.22 (2.4, 16.13)	1.70E-04
rs567253697	CDH2;MIR302F	18	28998467	0.003	A	2.27 (1.45, 3.55)	3.27E-04
rs72912563	MIR302F;DSC3	18	30370902	0.025	G	1.39 (1.16, 1.65)	2.53E-04
rs72929342	MIR302F;DSC3	18	30639537	0.0227	A	1.4 (1.17, 1.68)	2.52E-04
rs1258066005	TRAPPC8	18	31881708	0.001	G	3.92 (1.88, 8.16)	2.57E-04
rs77009623	ASXL3;NOL4	18	33800201	0.0081	T	0.42 (0.27, 0.65)	1.26E-04
rs147457770	NOL4;DTNA	18	34427532	0.0085	C	1.87 (1.42, 2.47)	9.51E-06
rs199867473	ELP2	18	36153986	0.0025	A	2.49 (1.52, 4.07)	3.03E-04
rs1267196812	FHOD3	18	36703289	0.0022	A	2.53 (1.52, 4.21)	3.47E-04
rs531621833	MIR4318;MIR924HG	18	38714015	0.001	C	4.01 (1.96, 8.22)	1.47E-04
rs10460065	LINC01477;KC6	18	41053711	0.3673	T	0.89 (0.83, 0.95)	2.86E-04
rs79549342	LINC00907	18	42566985	0.0277	A	1.38 (1.16, 1.63)	1.93E-04
rs888516522	RIT2	18	42744573	0.0005	CT	5E-13 (1.51E-20, 1.06E	2.61E-04
rs149177068	LINC01478	18	44418989	0.0242	C	1.39 (1.16, 1.67)	2.81E-04
rs542547630	ZBTB7C	18	48321262	0.0003	A	2E-06 (3.57E-09, 2.24E	1.75E-04
rs929177338		18	48886142	0.001	C	4.06 (1.95, 8.44)	1.74E-04
rs79056023	SMAD7	18	48937853	0.134	A	1.19 (1.09, 1.29)	5.26E-05
rs189750275	SMAD7;DYM	18	49009321	0.0052	G	1.91 (1.35, 2.7)	2.68E-04
rs370428783	DCC	18	52616905	0.0014	G	3.28 (1.76, 6.1)	1.82E-04
rs376524519	DCC	18	52991551	0.0038	T	2.26 (1.52, 3.38)	6.02E-05
rs546303207	DCC	18	53297415	0.0032	T	2.23 (1.45, 3.43)	2.56E-04
rs563616050	CDH20;LINC01544	18	61664462	0.0009	A	5.07 (2.35, 10.94)	3.64E-05
rs139283303	BCL2	18	63296490	0.0024	G	2.7 (1.64, 4.45)	9.17E-05
rs1351214332	LOC107985136	18	69380913	0.0017	A	2.87 (1.61, 5.12)	3.49E-04
rs147745761	DOK6	18	69442490	0.0093	G	1.73 (1.32, 2.28)	7.08E-05
rs74312573	CBLN2;NETO1	18	72591397	0.0071	G	1.77 (1.3, 2.41)	3.01E-04
rs528258559	LOC100505817;FBXO15	18	74017408	0.001	C	4.76 (2.22, 10.16)	5.74E-05
rs185874603	CYB5A	18	74288815	0.005	G	1.96 (1.36, 2.81)	2.61E-04
rs11662262	LINC01029;SALL3	18	78826126	0.0699	C	1.23 (1.1, 1.37)	1.82E-04
rs1467804519	SHC2	19	427455	0.0026	GGGGGAATTGCAC ACGGCACAGGGAA	2.67 (1.64, 4.34)	7.53E-05
rs181758278	PRSS57;PALM	19	701038	0.0222	C	1.43 (1.19, 1.73)	1.52E-04
rs4807423	NCLN;CELF5	19	3212963	0.3234	T	0.88 (0.83, 0.94)	1.14E-04

rs563138794	C3	19	6705305	0.0031	A	2.36 (1.53, 3.66)	1.19E-04
rs1207289723	CARM1	19	10878214	0.0031	A	2.33 (1.5, 3.62)	1.81E-04
rs972273213	KANK2	19	11168101	0.0029	C	2.45 (1.56, 3.84)	9.38E-05
rs554314974	DOCK6	19	11216557	0.0014	G	3.31 (1.75, 6.28)	2.45E-04
rs190013476	PLPPR2	19	11356460	0.0015	G	3.55 (1.93, 6.54)	4.68E-05
rs2311784	ZNF439;ZNF69	19	11884693	0.0511	C	1.28 (1.13, 1.45)	1.38E-04
rs4926281	CACNA1A	19	13371078	0.4946	A	1.12 (1.05, 1.18)	2.58E-04
rs10409463	CACNA1A;CCDC130	19	13521569	0.2956	T	0.88 (0.83, 0.94)	1.26E-04
rs77132734	CACNA1A;CCDC130	19	13521840	0.0727	G	0.79 (0.7, 0.89)	1.49E-04
chr19:14875834_A_AACAAT		19	14875834	0.0044	A	2.02 (1.38, 2.95)	2.84E-04
rs190656539	CYP4F24P	19	15761255	0.0017	C	3.19 (1.79, 5.67)	8.08E-05
rs1410302666	NWD1	19	16725061	0.0014	G	3.17 (1.7, 5.92)	2.87E-04
rs17214879	SLC25A42;TMEM161A	19	19118442	0.065	T	0.79 (0.7, 0.9)	2.34E-04
rs3761081	BORCS8;BORCS8-MEF2B	19	19190377	0.143	G	1.17 (1.08, 1.28)	1.99E-04
rs146594810	ZNF626	19	20642632	0.0196	A	1.47 (1.22, 1.78)	6.74E-05
rs1406044895	ZNF708	19	21297628	0.0028	T	2.5 (1.58, 3.96)	9.13E-05
rs142573397	ZNF208;ZNF257	19	22049663	0.0028	GA	2.37 (1.49, 3.75)	2.46E-04
rs141146165	C19orf12;CCNE1	19	29741535	0.0125	T	1.63 (1.29, 2.05)	3.12E-05
rs10413450	ZNF536;LINC01791	19	31063164	0.0572	G	0.77 (0.68, 0.89)	2.00E-04
rs190512420	ZNF536;LINC01791	19	31153318	0.0009	T	4.24 (1.94, 9.28)	2.99E-04
rs1436725754	TSHZ3-AS1	19	31381672	0.0015	TATTC	3.63 (2, 6.6)	2.34E-05
rs1016558007	EID2;LGALS13	19	39559809	0.0012	C	3.43 (1.75, 6.74)	3.48E-04
rs923525094		19	40434509	0.0007	G	5.37 (2.17, 13.3)	2.85E-04
rs182068754	CEACAM4	19	41626434	0.0059	G	1.85 (1.33, 2.57)	2.89E-04
rs139010509	PSG8;PSG8-AS1	19	42781864	0.0239	T	1.46 (1.22, 1.75)	3.24E-05
rs193073987	PSG2;PSG5	19	43164512	0.0014	T	3.22 (1.7, 6.1)	3.20E-04
rs767242893	PPP1R37	19	45098080	0.0006	TG	7.62 (2.78, 20.84)	7.67E-05
rs116899842	HIF3A	19	46330398	0.0056	A	1.94 (1.38, 2.72)	1.26E-04
rs542101185	CCDC8;PNMA8A	19	46448641	0.0016	C	3.14 (1.75, 5.62)	1.17E-04
rs182835388	MIR519A2;MIR371A	19	53775529	0.0031	A	2.32 (1.49, 3.62)	2.00E-04
rs8106823	LAIR1	19	54363513	0.0505	A	0.75 (0.65, 0.87)	1.63E-04
rs4806764	CDC42EP5;LAIR2	19	54491428	0.3728	T	0.89 (0.84, 0.95)	2.69E-04
rs147144361	FAM110A;ANGPT4	20	863935	0.0027	T	2.41 (1.52, 3.83)	1.98E-04
rs2076651	ZNF343	20	2493239	0.054	G	1.27 (1.13, 1.44)	8.78E-05
rs145786689	ADRA1D;PRNP	20	4508686	0.0032	T	2.26 (1.45, 3.5)	2.80E-04
rs362986	SNAP25	20	10296386	0.0997	T	1.19 (1.08, 1.3)	3.38E-04
rs199583179	MACROD2	20	14000304	0.0012	C	3.62 (1.84, 7.14)	1.96E-04

rs201011289	FLRT3;MACROD2	20	14333659	0.0012	G	3.87 (1.94, 7.72)	1.26E-04
rs149327473	MACROD2	20	15310634	0.0025	C	2.66 (1.63, 4.34)	8.46E-05
rs55892570	KIF16B	20	16505479	0.0011	A	4.16 (2.07, 8.36)	6.47E-05
rs369506594	OTOR;PCSK2	20	16913673	0.0007	G	5.83 (2.31, 14.74)	1.95E-04
rs1164778012	LINC02967	20	24920620	0.0022	T	2.7 (1.61, 4.52)	1.56E-04
rs182864932	NINL	20	25580355	0	G	E+06 (2.99E+03, 6.51E	6.43E-06
rs1444702257		20	25862044	0.0018	C	3.25 (1.85, 5.7)	4.15E-05
rs1386366488	BCL2L1	20	31664483	0.0019	C	2.87 (1.62, 5.06)	2.79E-04
rs1286247392	NOL4L	20	32466917	0.0017	C	3.2 (1.76, 5.82)	1.44E-04
rs149831754	BPIFB3	20	33065910	0.0047	G	2.03 (1.41, 2.92)	1.53E-04
rs536500038	CBFA2T2	20	33586833	0.0051	A	2.05 (1.45, 2.9)	5.31E-05
rs6142008	PXMP4;ZNF341	20	33726588	0.184	G	1.21 (1.12, 1.3)	9.14E-07
rs563003201	EPB41L1	20	36151830	0.0031	G	2.64 (1.71, 4.08)	1.23E-05
rs184842078	DLGAP4-AS1	20	36571417	0.0061	C	0.36 (0.21, 0.62)	2.47E-04
rs191141885	CTNNBL1;VSTM2L	20	37877770	0.0014	C	3.4 (1.81, 6.36)	1.33E-04
rs186044902	ZHX3;LPIN3	20	41328517	0.004	G	2.23 (1.51, 3.28)	5.34E-05
rs7260790	LINC01728;TOX2	20	43898876	0.1745	G	1.15 (1.07, 1.24)	3.47E-04
rs941255971	JPH2	20	44123826	0.0004	C	11.43 (3.02, 43.29)	3.37E-04
rs6073604	STK4	20	45023486	0.021	G	1.44 (1.19, 1.74)	2.04E-04
rs1167068174	WFDC5	20	45117898	0.0008	G	4.79 (2.13, 10.76)	1.48E-04
rs1415149484	WFDC10B	20	45699316	0.0006	AT	9.06 (3.15, 26.07)	4.39E-05
rs140377390	WFDC13;SPINT4	20	45717734	0.0344	C	0.7 (0.58, 0.84)	9.62E-05
rs1171543348	ZNF334	20	46475740	0.0009	AT	5.2 (2.36, 11.46)	4.30E-05
rs576832072	SLC13A3	20	46589588	0.001	T	3.81 (1.84, 7.88)	3.13E-04
rs375579017	ZMYND8	20	47252099	0.0056	G	1.97 (1.4, 2.78)	9.82E-05
rs557194608	LINC01522;LINC00494	20	48091775	0.0009	A	4.2 (1.94, 9.1)	2.81E-04
rs2426306	NFATC2	20	51410071	0.0554	G	0.76 (0.66, 0.88)	1.35E-04
rs117686750	LINC01441;CBLN4	20	55494294	0.0023	G	2.7 (1.62, 4.51)	1.50E-04
rs74653233	EDN3;PHACTR3	20	59453267	0.1215	C	0.82 (0.74, 0.9)	2.19E-05
rs1244253588	PHACTR3	20	59641274	0.0009	C	4.15 (1.94, 8.9)	2.51E-04
rs1439771382		20	60364621	0.0008	A	4.85 (2.09, 11.29)	2.47E-04
rs532112228	LINC02246;NRIP1	21	14935813	0.0032	C	2.23 (1.45, 3.45)	2.74E-04
rs146483804	MIR99AHG	21	16289848	0.0021	A	2.81 (1.63, 4.83)	2.00E-04
rs202110461	MIR99AHG;LINC01549	21	17219701	0.0124	AAAG	0.54 (0.39, 0.75)	2.20E-04
rs1301887243	CXADR	21	17614827	0.0028	C	2.42 (1.52, 3.84)	1.84E-04
rs1199328509		21	23244224	0.0014	G	3.18 (1.7, 5.96)	3.11E-04
rs2409300	LINC01695;LINC00161	21	28350952	0.0073	T	1.77 (1.31, 2.4)	2.25E-04
rs182279350	LINC01695;LINC00161	21	28486021	0.0161	C	1.49 (1.2, 1.84)	2.36E-04

rs147971798	CLDN17;LINC00307	21	30172285	0.0036	A	2.33 (1.55, 3.52)	5.57E-05
rs8131170	CLDN8;KRTAP24-1	21	30237849	0.0618	A	1.28 (1.14, 1.43)	3.81E-05
rs844991	EVA1C;C21orf59-TCP10L	21	32565749	0.3427	T	1.14 (1.07, 1.21)	4.69E-05
rs145939560	MIR802;PPP1R2P2	21	35871913	0.0235	G	1.39 (1.17, 1.66)	2.33E-04
rs553699963	DYRK1A	21	37368079	0.0041	G	2.14 (1.44, 3.18)	1.81E-04
rs914360673	B3GALT5;IGSF5	21	39705361	0.0017	T	3 (1.7, 5.31)	1.50E-04
rs2837830	DSCAM	21	40804305	0.4332	C	0.87 (0.82, 0.93)	7.07E-06
rs1263590752		21	41462406	0.0007	C	5.51 (2.26, 13.43)	1.71E-04
rs1346115182	LINC01671	21	42608472	0.0009	A	4.22 (1.95, 9.13)	2.65E-04
rs371363600		21	43286835	0.0008	G	5.3 (2.24, 12.51)	1.42E-04
rs149249350	TRPM2	21	44365691	0.0012	C	3.4 (1.74, 6.65)	3.30E-04
chr21:44374015_C_CT		21	44374015	0.0054	C	1.94 (1.36, 2.75)	2.22E-04
rs547728079	LINC00895;SEPT5	22	19600858	0.0016	C	3.01 (1.66, 5.45)	2.92E-04
rs987404897	MED15;POM121L4P	22	20623163	0.0006	A	6.59 (2.49, 17.42)	1.46E-04
rs146546335	PI4KA	22	20709832	0.0713	G	1.23 (1.1, 1.37)	2.62E-04
rs1398418158	PIWIL3	22	24754391	0.0011	A	3.95 (1.93, 8.09)	1.68E-04
rs184527621	MYO18B;SEZ6L	22	26130160	0.0016	C	3.2 (1.77, 5.78)	1.23E-04
rs78698788	LOC100507657;MN1	22	27435114	0.0246	G	1.41 (1.19, 1.68)	1.05E-04
rs117540652	RFPL1;NEFH	22	29469858	0.0063	C	1.95 (1.42, 2.67)	3.41E-05
rs1221413018	ASCC2	22	29805548	0.0024	C	2.46 (1.51, 4.01)	2.79E-04
rs144649642	LARGE1;ISX-AS1	22	33942199	0.0012	G	4.79 (2.44, 9.41)	5.19E-06
rs368630022	EIF3D	22	36517225	0.0012	A	3.88 (1.94, 7.73)	1.20E-04
rs148009193	CBX6;APOBEC3A	22	38893153	0.0378	G	1.35 (1.16, 1.57)	1.05E-04
rs758519032	MEI1	22	41716543	0.0006	ATT	6.07 (2.27, 16.2)	3.22E-04
rs12158335	TNFRSF13C;CENPM	22	41928618	0.0988	T	1.21 (1.1, 1.33)	6.68E-05
rs141157608	GTSE1	22	46331160	0.0034	G	2.24 (1.47, 3.42)	1.80E-04
rs543982379	FAM19A5	22	48616788	0.0015	G	3.35 (1.82, 6.19)	1.08E-04
rs6010477	MIR4535;LINC01310	22	48798137	0.2628	C	1.13 (1.06, 1.2)	3.11E-04
rs377334676	LINC01310;NONE	22	49345354	0.0043	G	2.11 (1.43, 3.11)	1.64E-04
